# Supplementary figures and images for: Knowledge-guided data mining on the standardized architecture of NRPS: Subtypes, novel motifs, and sequence entanglements
Source: PLoS Comput Biol. 2023 May 15;19(5):e1011100. doi: 10.1371/journal.pcbi.1011100 (PMC10212144; doi:10.1371/journal.pcbi.1011100)

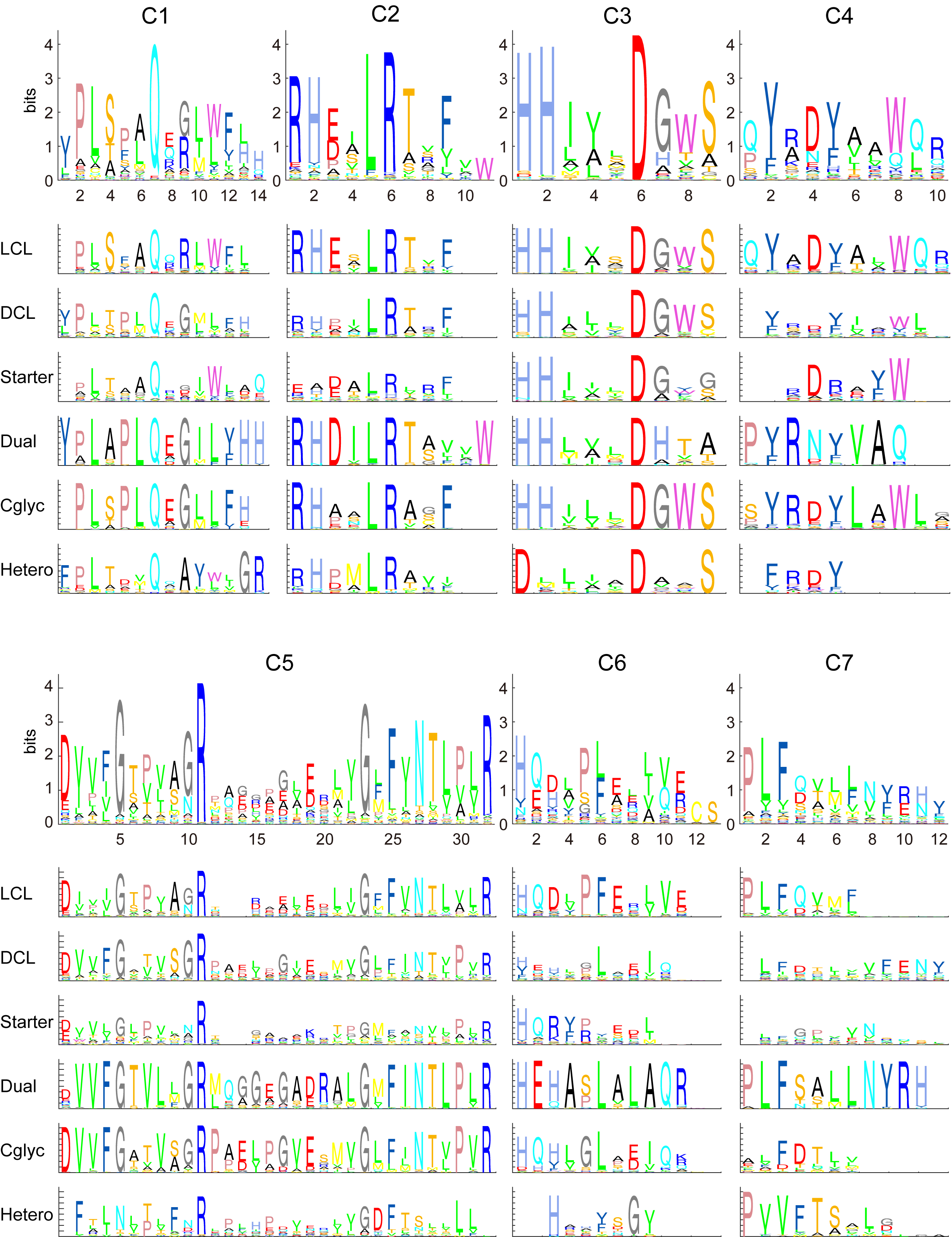

Supplement: S1 Fig — The ranges of y-axis in sequence logo figures all are 0~4.4 bits. The numbers of each C domain subtypes are 809 (LCL), 385 (DCL), 114 (Starter), 300 (Dual), 114 (Cgly), and 36 (Heterocyclization). (PNG) [file pcbi.1011100.s001.png]

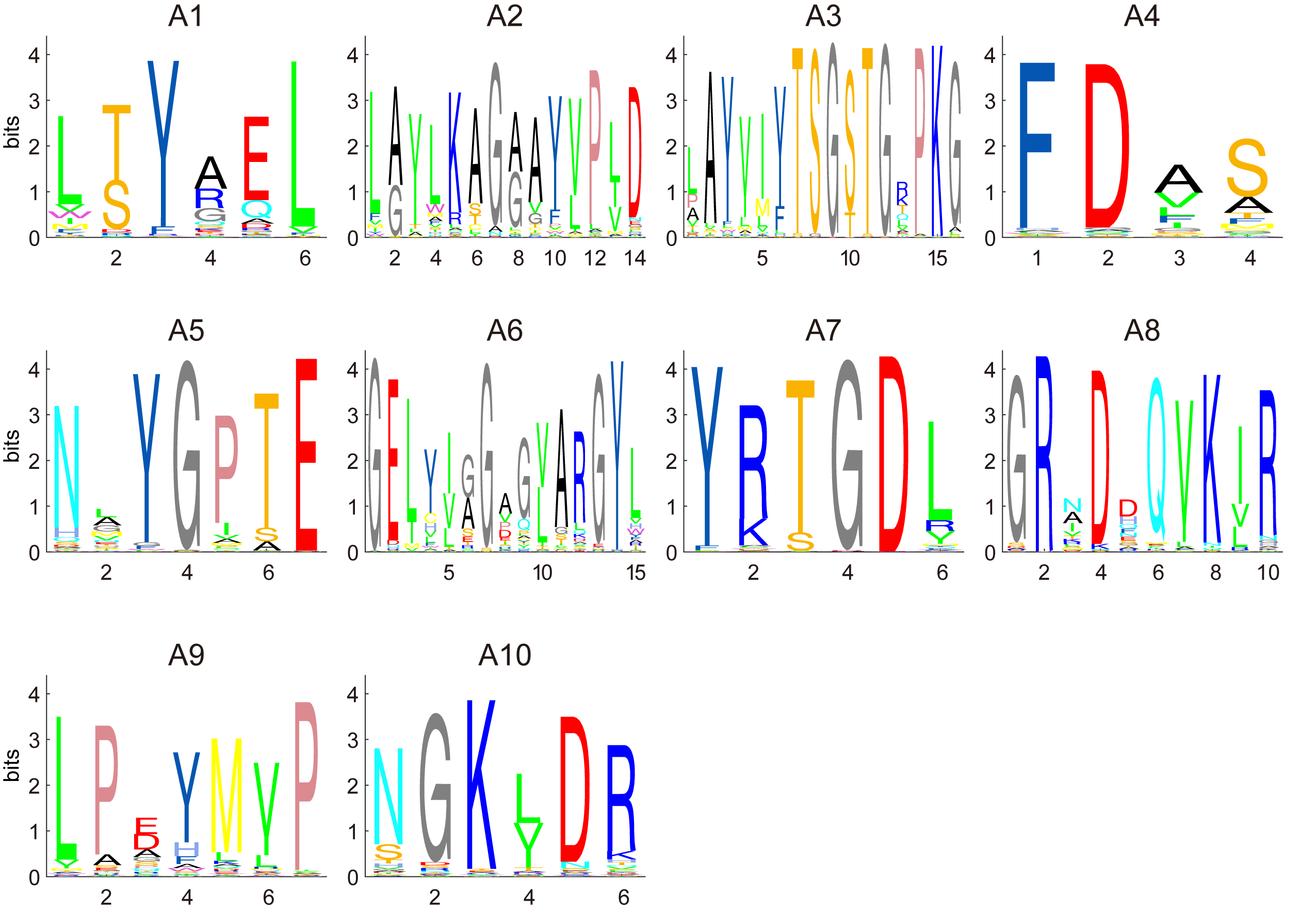

Supplement: S2 Fig — The y-axis ranges in sequence logo figures all are 0~4.4 bits. (PNG) [file pcbi.1011100.s002.png]

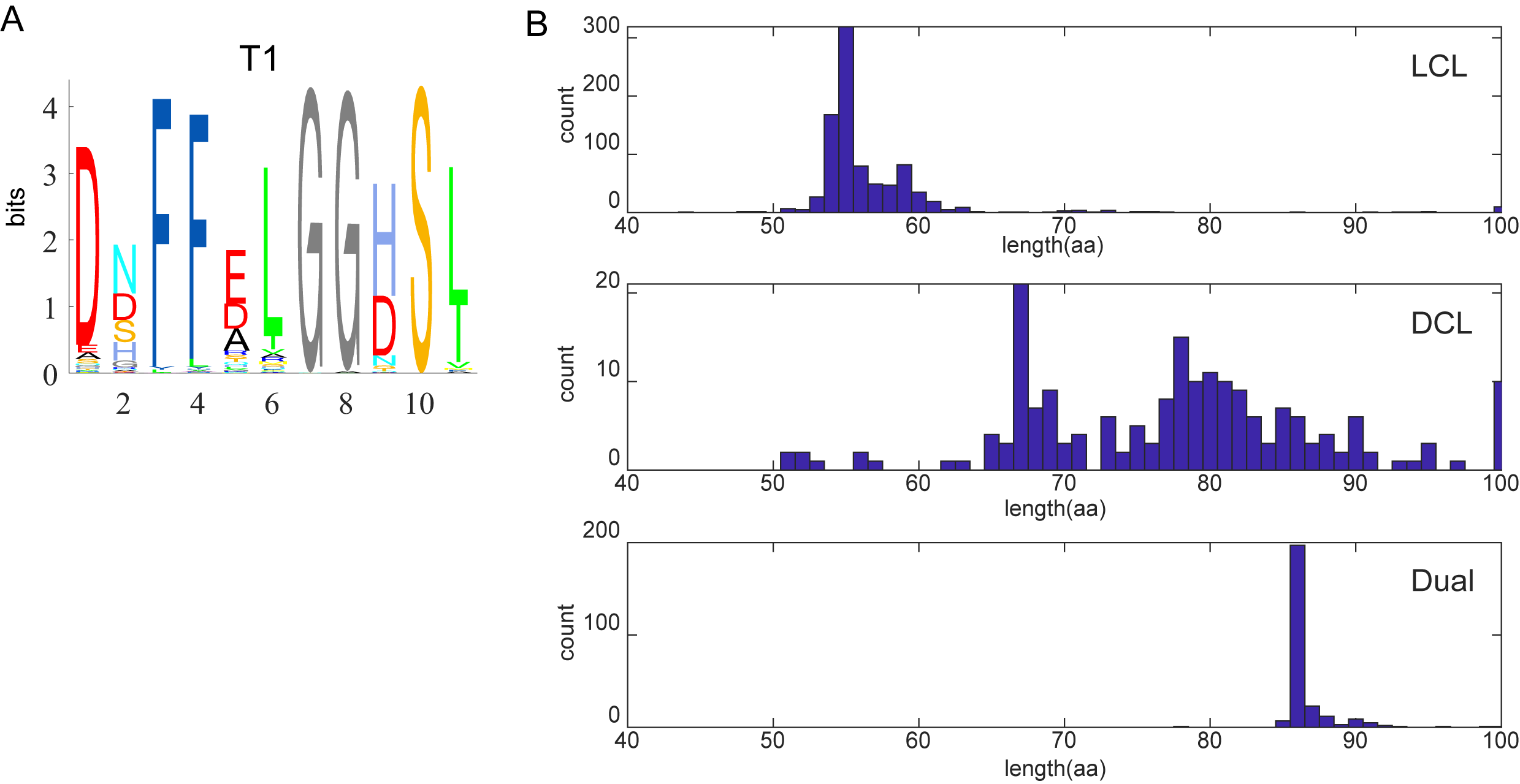

Supplement: S3 Fig — A. Sequence logo of the T1 motif. The y-axis range in sequence logo figure is 0~4.4 bits. B. Length distribution of the T1-C1 region for different subtypes of C. (PNG) [file pcbi.1011100.s003.png]

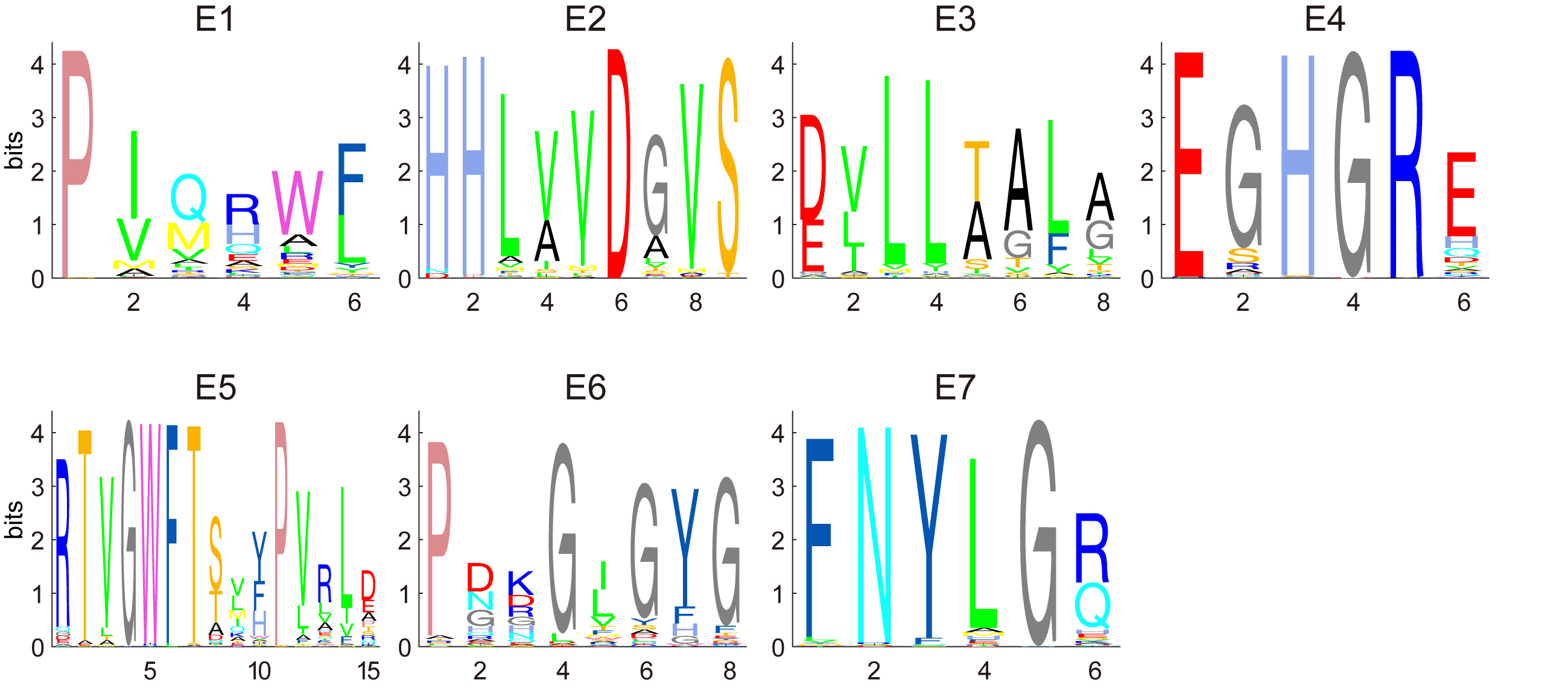

Supplement: S4 Fig — The y-axis ranges in sequence logo figures all are 0~4.4 bits. (PNG) [file pcbi.1011100.s004.png]

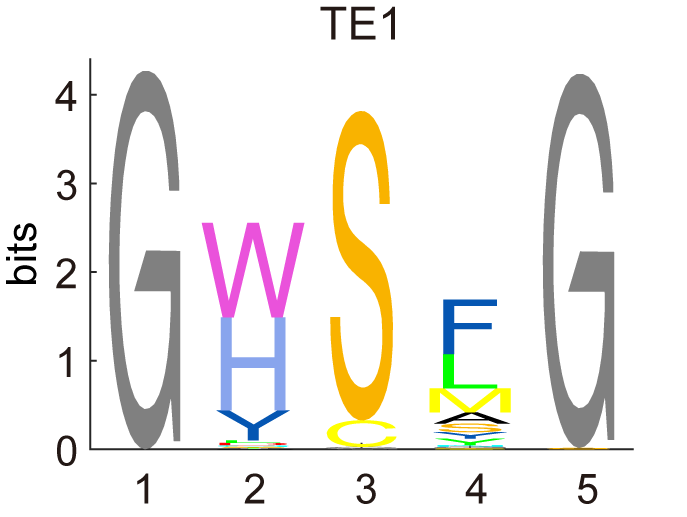

Supplement: S5 Fig — The y-axis range in sequence logo figure is 0~4.4 bits. (PNG) [file pcbi.1011100.s005.png]

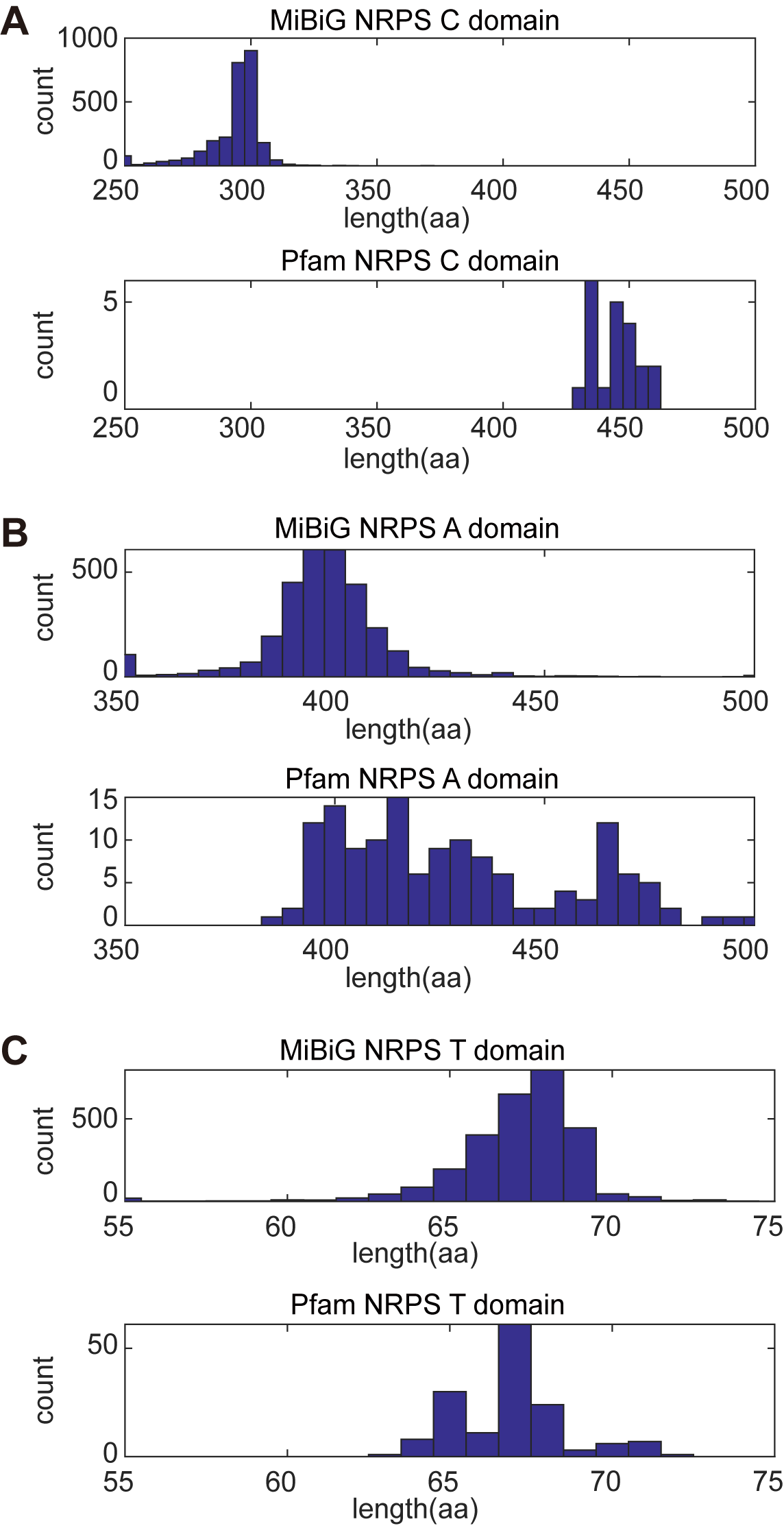

Supplement: S6 Fig — (PNG) [file pcbi.1011100.s006.png]

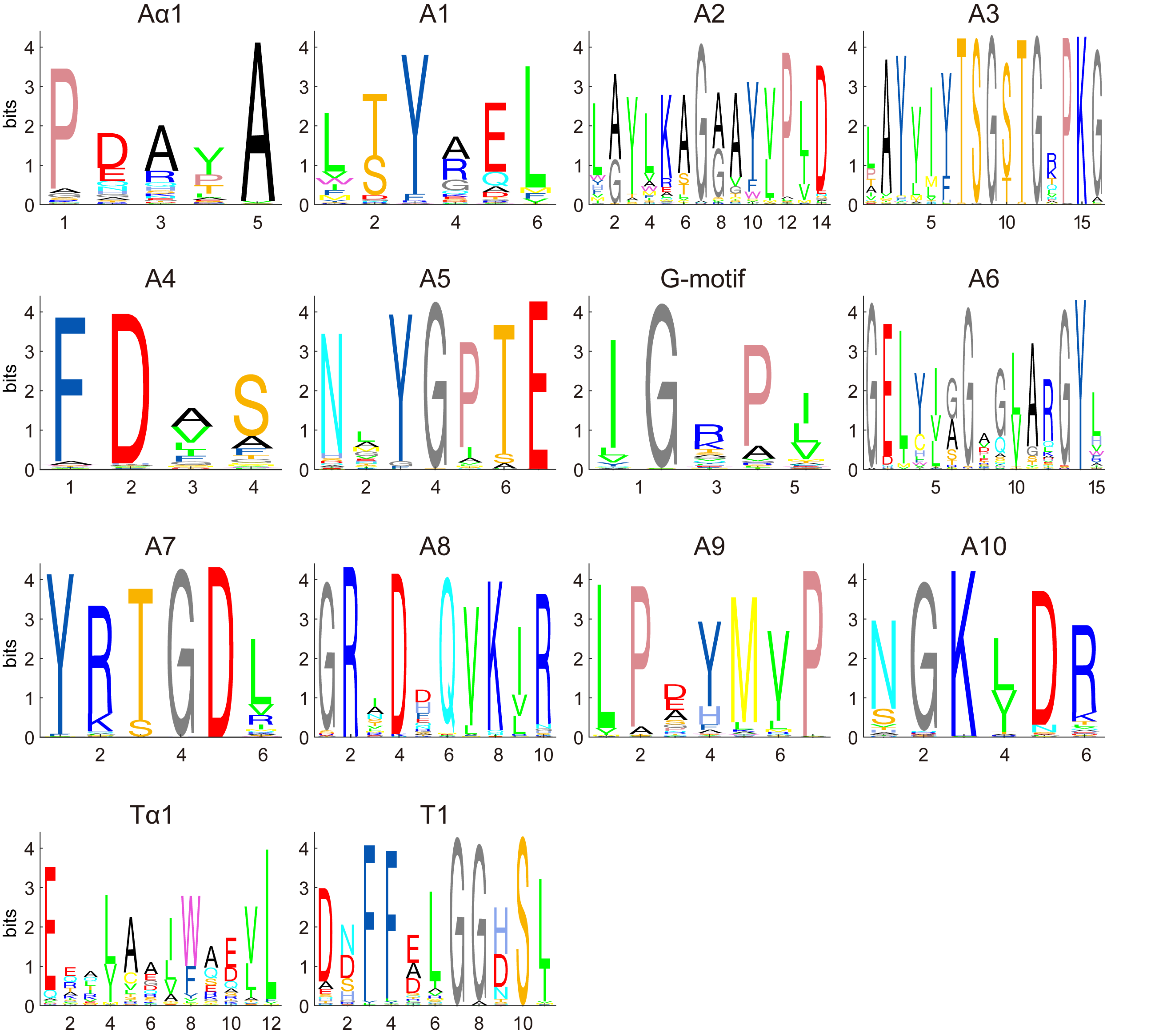

Supplement: S7 Fig — The y-axis ranges in sequence logo figures all are 0~4.4 bits. (PNG) [file pcbi.1011100.s007.png]

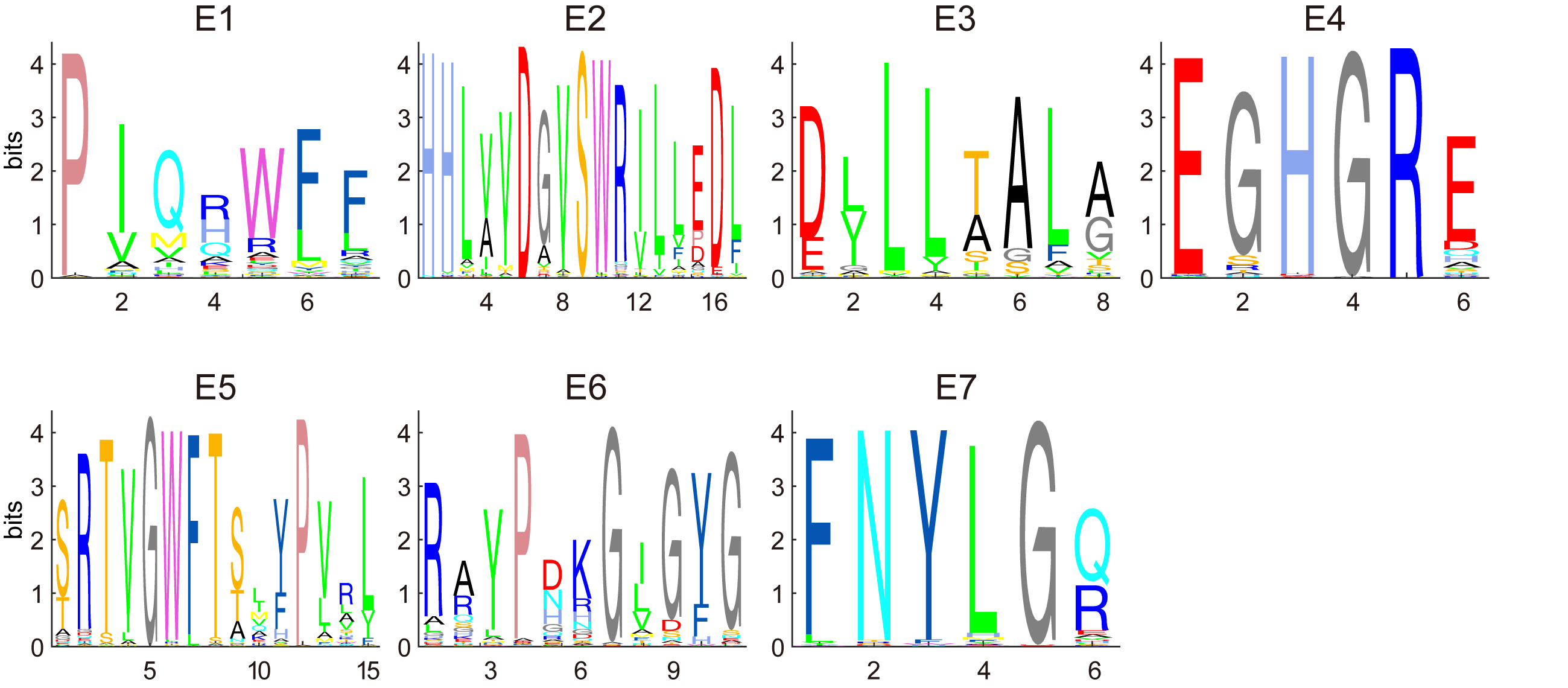

Supplement: S8 Fig — The y-axis ranges in sequence logo figures all are 0~4.4 bits. (PNG) [file pcbi.1011100.s008.png]

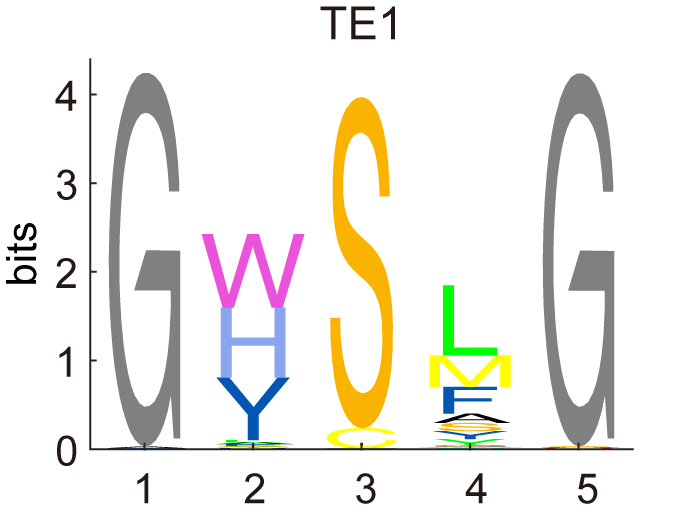

Supplement: S9 Fig — The y-axis range in the sequence logo figure is 0~4.4 bits. (PNG) [file pcbi.1011100.s009.png]

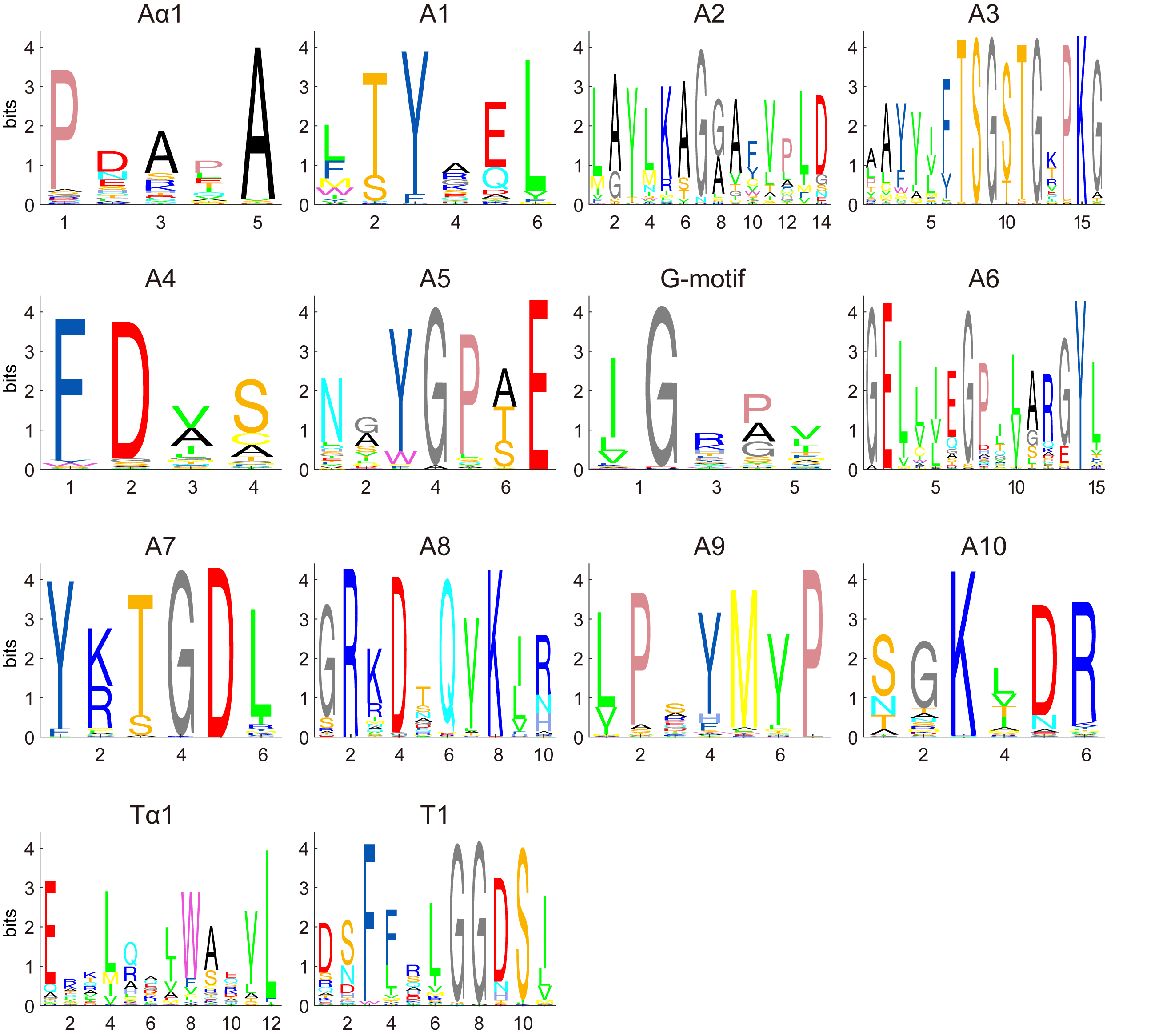

Supplement: S10 Fig — The y-axis ranges in sequence logo figures all are 0~4.4 bits. (PNG) [file pcbi.1011100.s010.png]

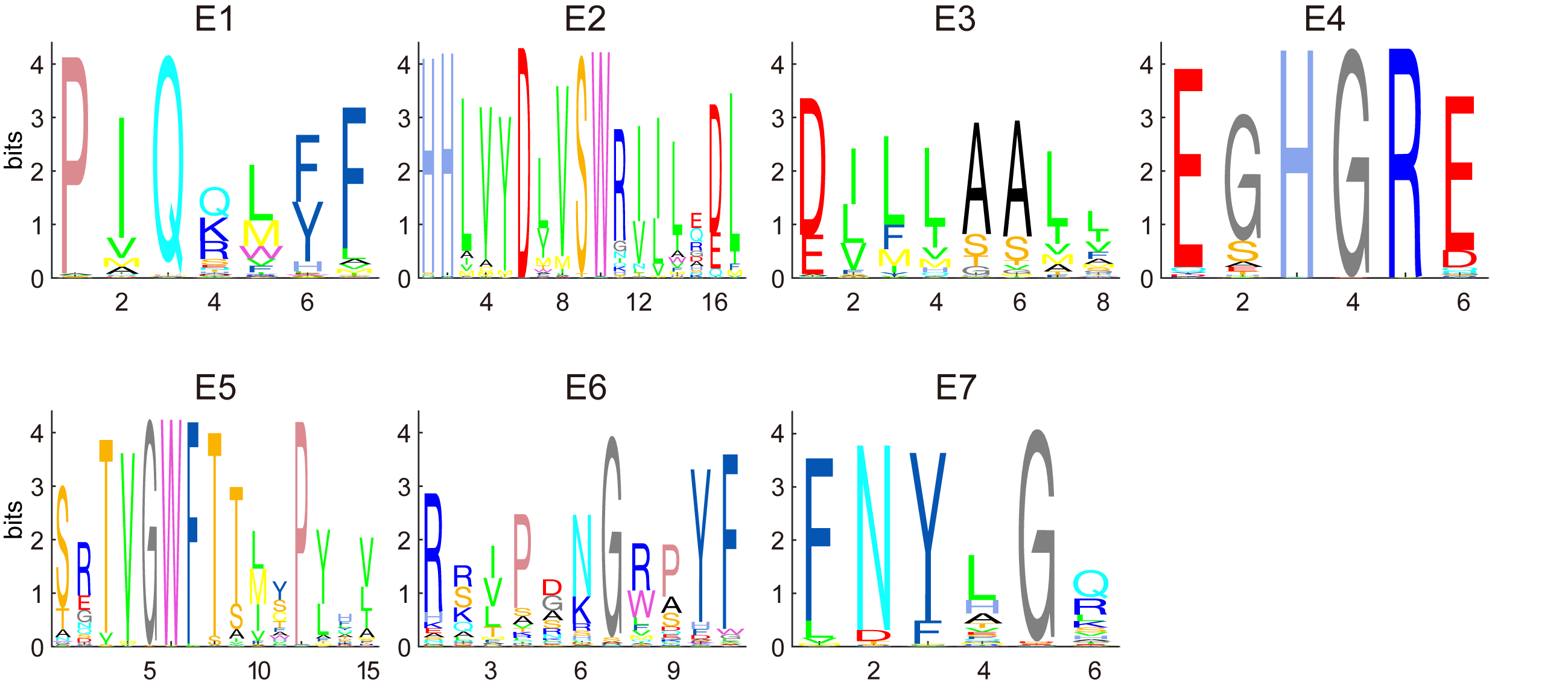

Supplement: S11 Fig — The y-axis ranges in sequence logo figures all are 0~4.4 bits. (PNG) [file pcbi.1011100.s011.png]

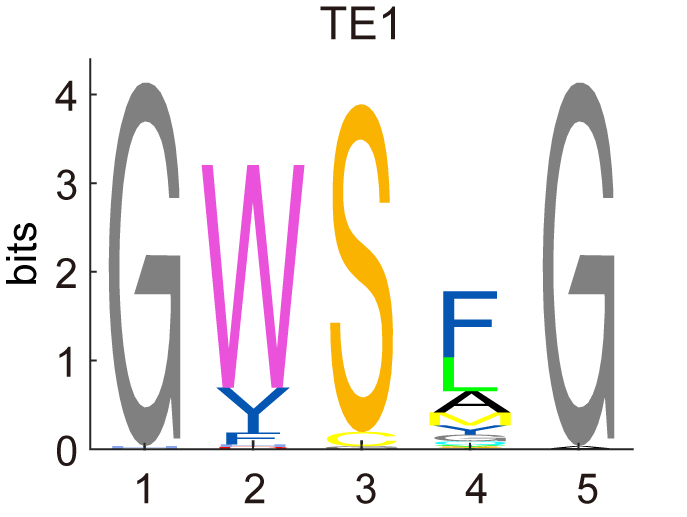

Supplement: S12 Fig — The y-axis range in sequence logo figures is 0~4.4 bits. (PNG) [file pcbi.1011100.s012.png]

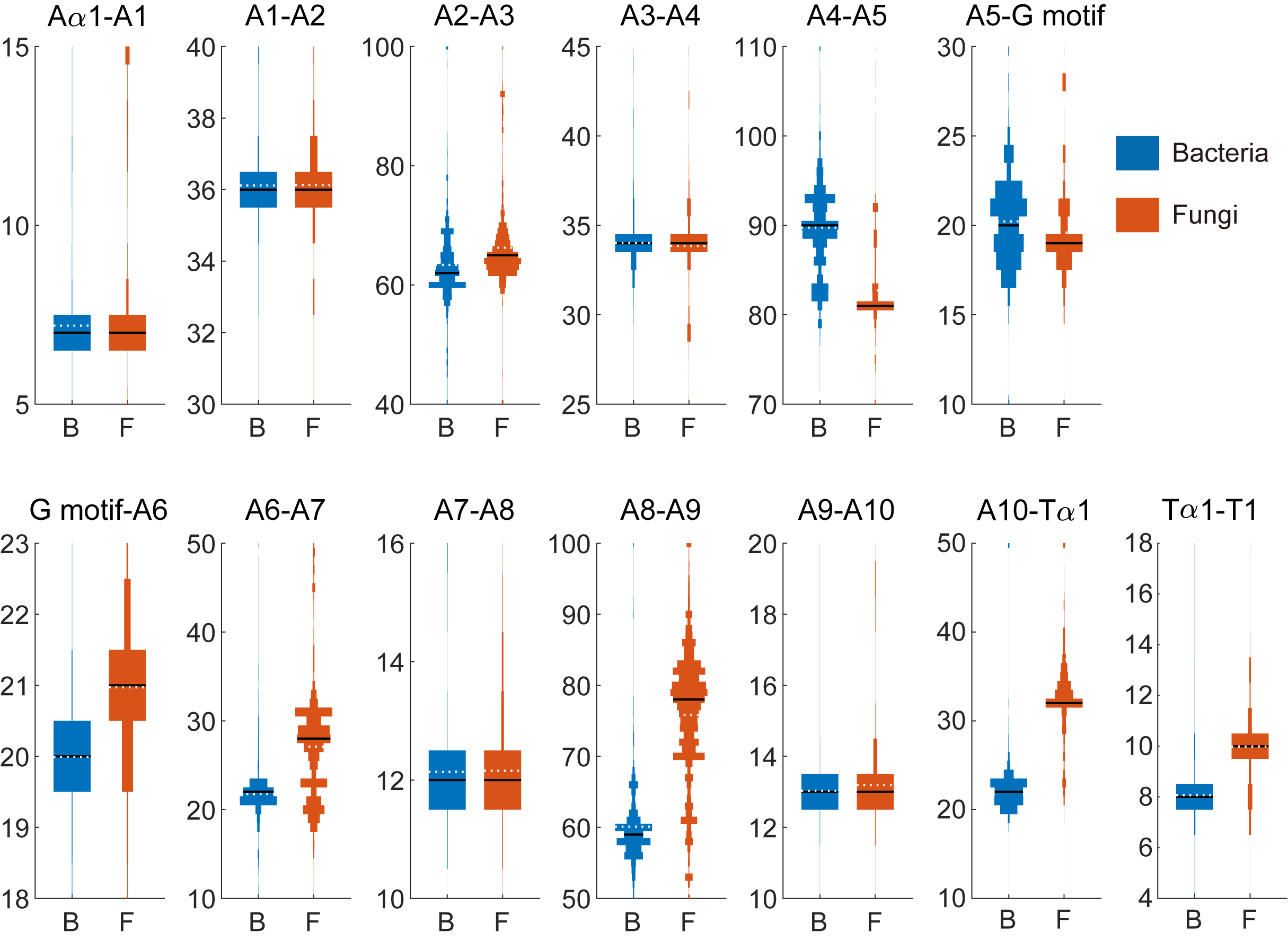

Supplement: S13 Fig — For comparison, only A domains which have the same motif length with reference A domain are used. Sequence numbers of intermotifs (A1-A2, A2-A3, A3-A4, A4-A5, A5-G motif, G motif-A6, A6-A7, A7-A8, A8-A9, A9-A10, Tα1-T1) are 75,407 in bacteria and 17,890 in fungi. Sequence numbers of A1-Tα1 intermotif (actually interdomain) in bacteria source are 69,440 while they in fungi source are 12,194 because only part of A domains are adjacent with the T domain. Sequence numbers of Tα1-T1 intermotif in bacteria are 85,755 while they in fungi are 25,069. (PNG) [file pcbi.1011100.s013.png]

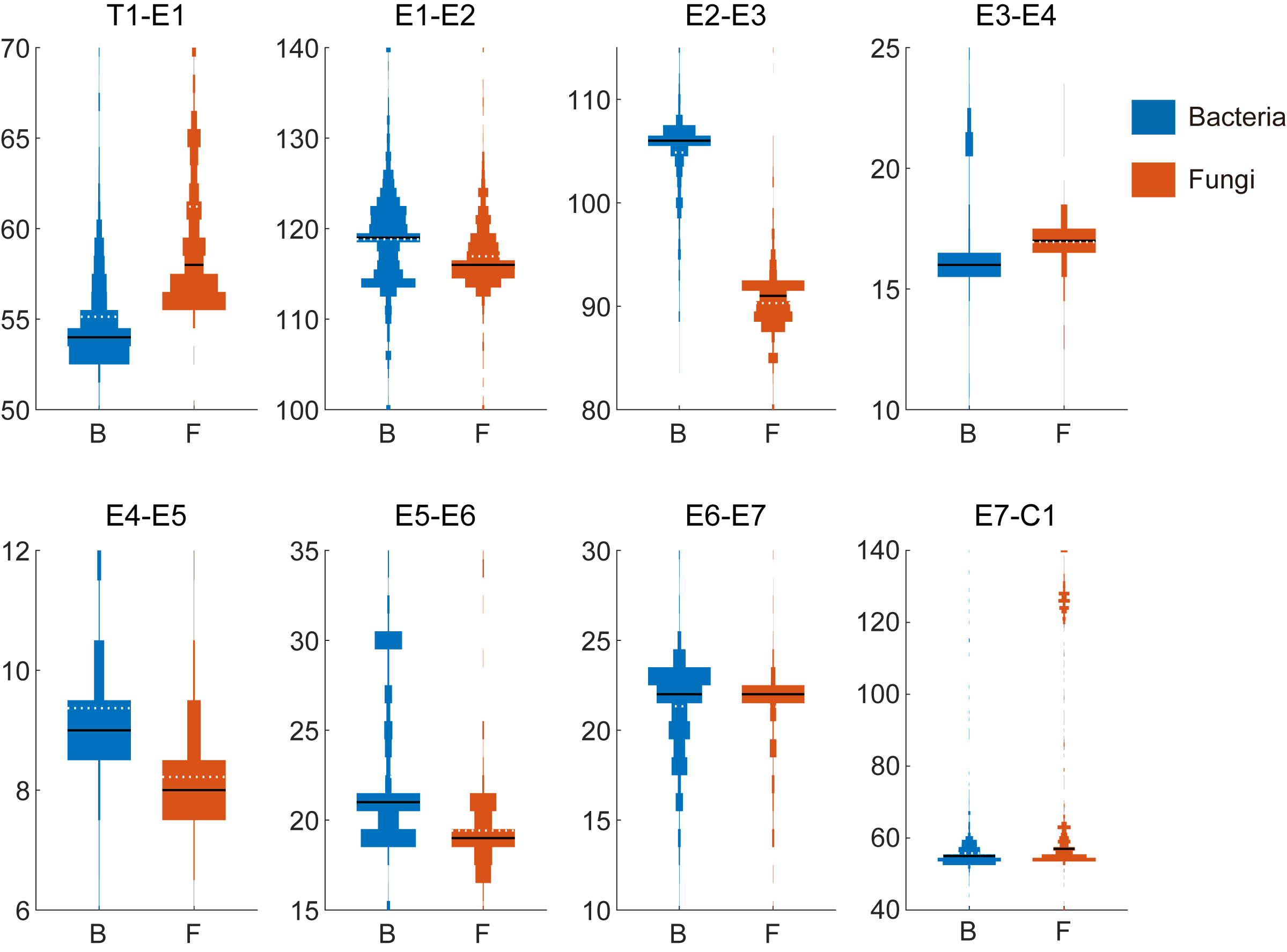

Supplement: S14 Fig — For comparison, only A domains which have same motif length with reference A domain are used. Sequence numbers of intermotifs (E1-E2, E2-E3, E3-E4, E4-E5, E5-E6, E6-E7) are 12,875 in bacteria source and 2,852 in fungi source. Sequence numbers of intermotifs (actually interdomain) in bacteria are 12,618 for T1-E1 and 8,353 for E7-C1 while they in fungi are 2,088 for T1-E1 and 2,530 for E7-C1. (PNG) [file pcbi.1011100.s014.png]

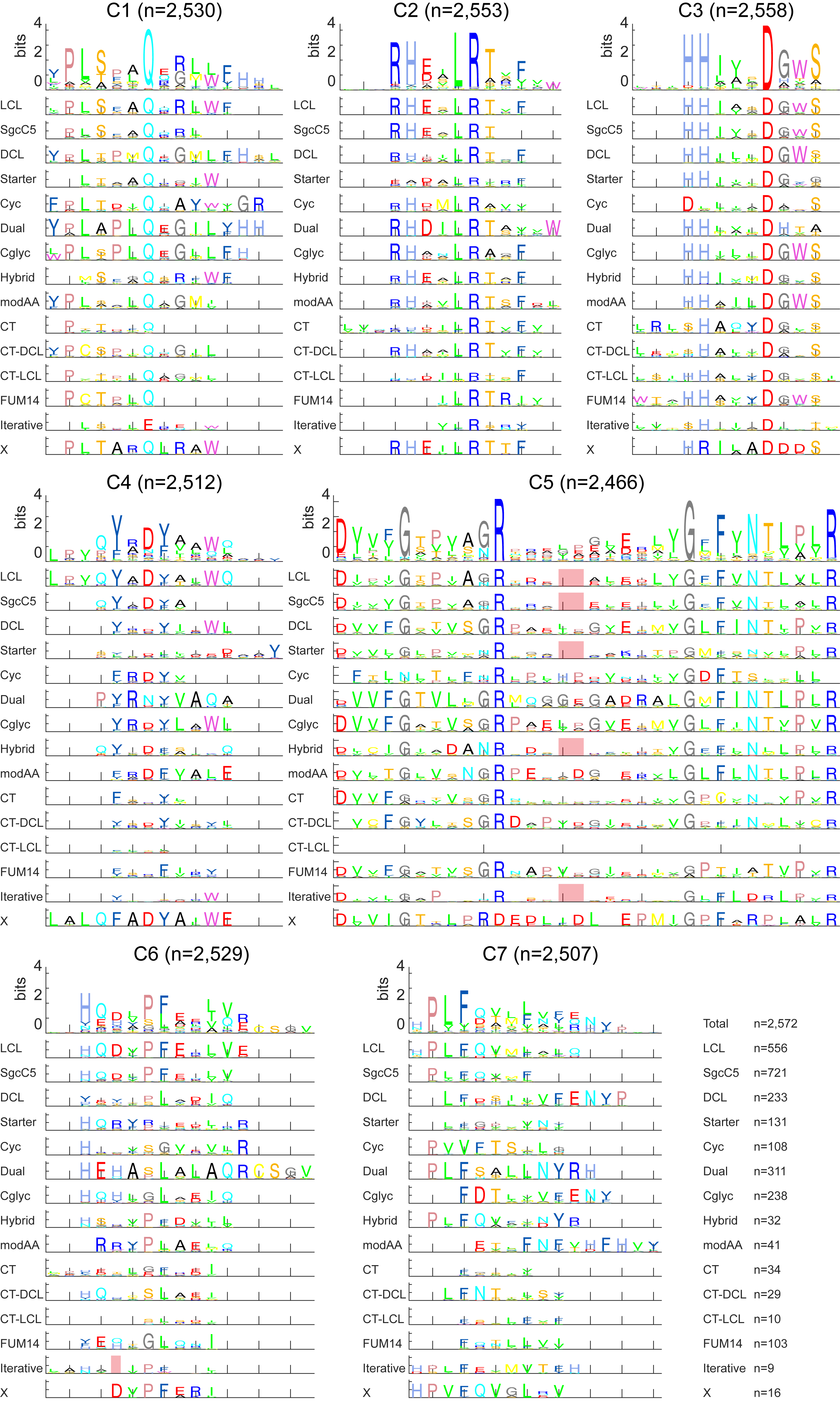

Supplement: S15 Fig — There are 2,572 C domains with subtype prediction scores more than the threshold (200) and a count of domain subtype sequences of more than 3. The y-axis ranges in sequence logo figures all are 0~4.4 bits. The numbers of each C domain subtype are labeled at the end. For clarification, only motifs that have prevalent length are used in plotting. The actual total C domain number used in the figure for the specific motif is shown in the title. There are some gaps at both ends of the sequence for alignment. In motif C5, there are some interior gaps labeled in red to align with motif C5 of other subtypes. (PNG) [file pcbi.1011100.s015.png]

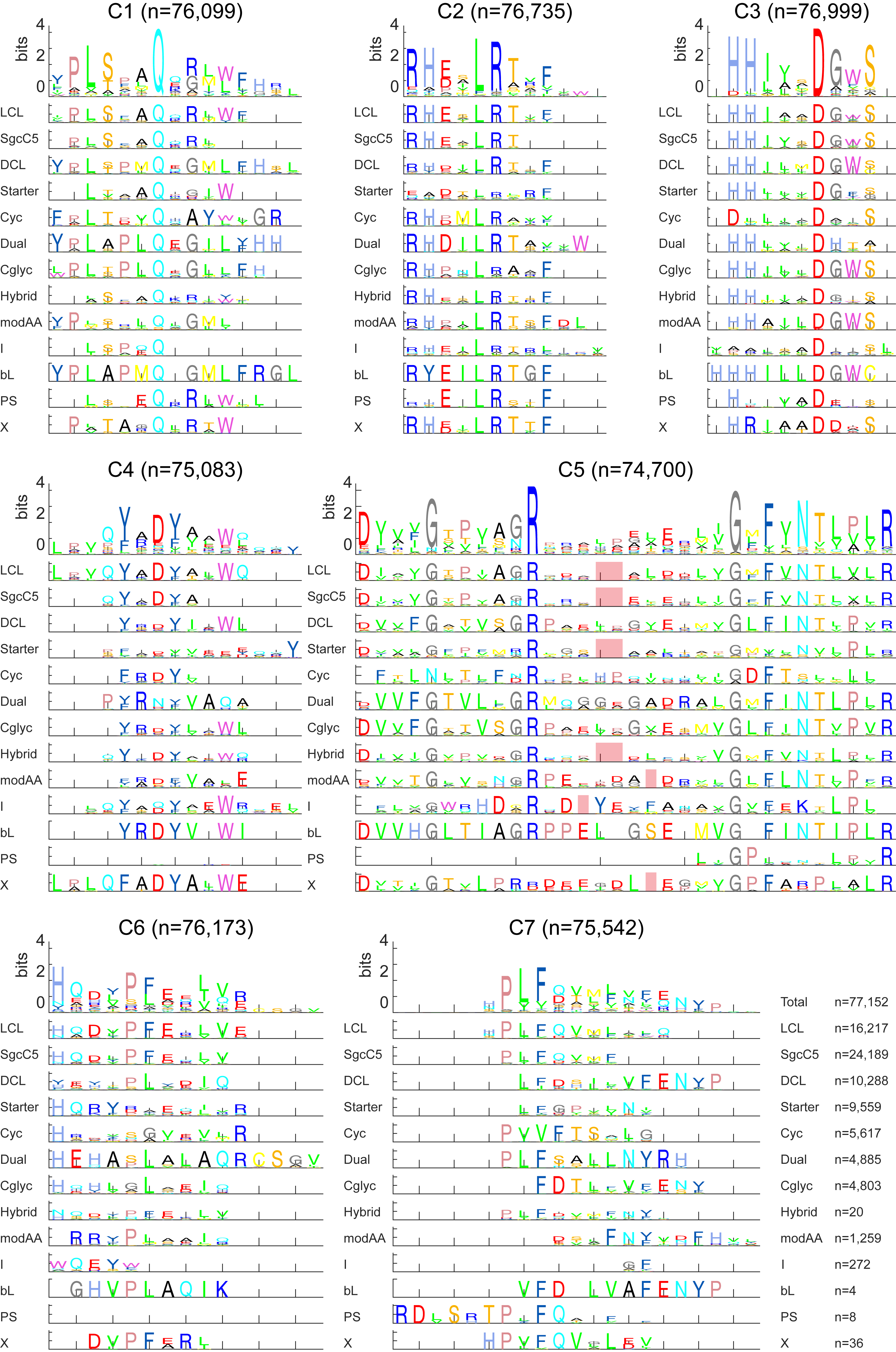

Supplement: S16 Fig — There are 77,152 C domains with subtype prediction scores more than the threshold (200) and a count of domain subtype sequences of more than 3. The y-axis ranges in sequence logo figures all are 0~4.4 bits, except it’s 0~1 for the bL subtype and 0~2.1 for the PS subtype because these subtypes are few in the sequence number. The numbers of each C domain subtype are labeled at the end. For clarification, only motifs that have prevalent length are used in plotting. The actual total C domain number used in the figure for the specific motif is shown in the title. There are some gaps at both ends of the sequence for alignment. In motif C5, there are some interior gaps labeled in red to align with motif C5 of other subtypes. (PNG) [file pcbi.1011100.s016.png]

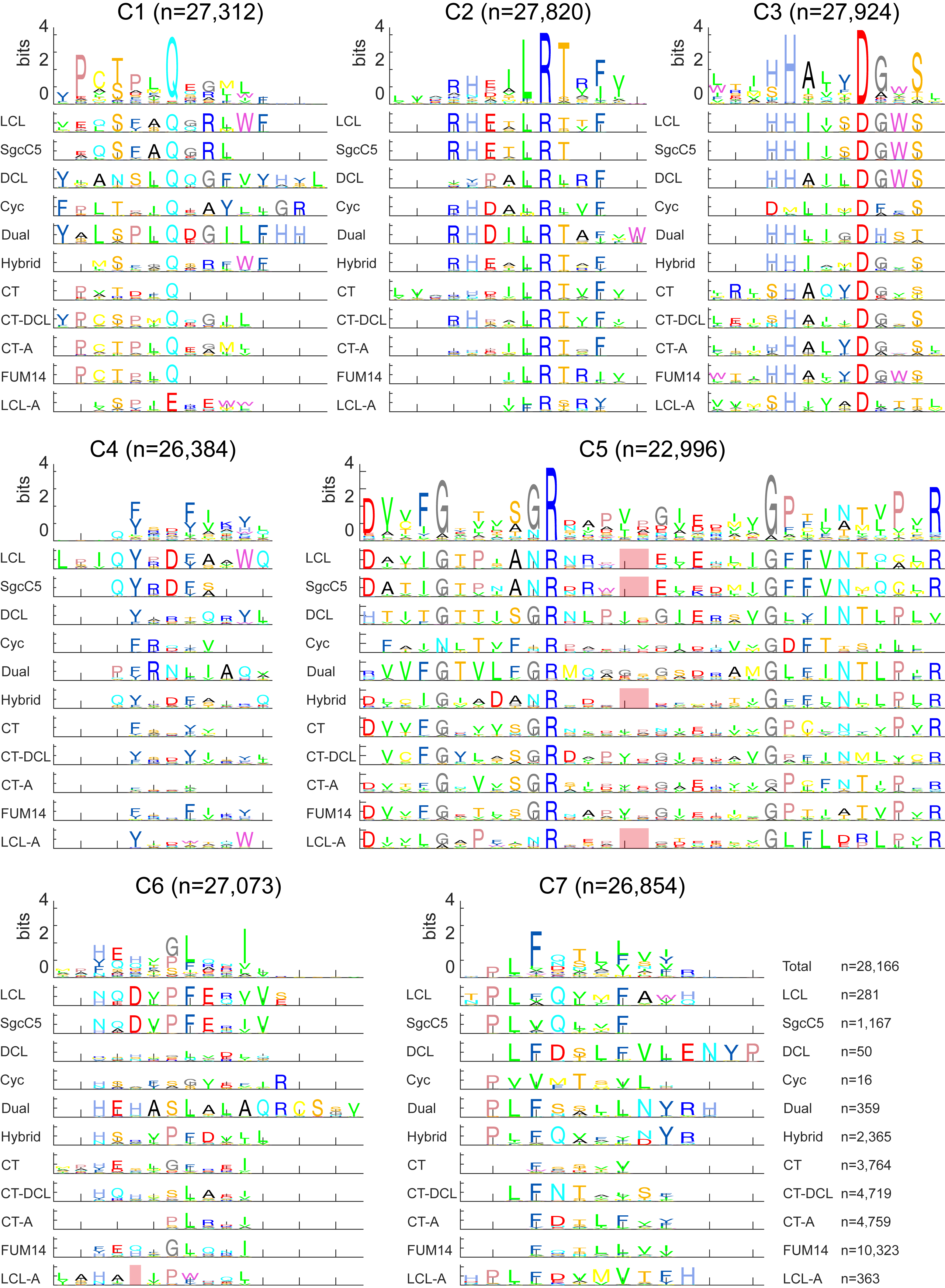

Supplement: S17 Fig — There are 34,269 C domains with subtype prediction scores more than the threshold (200) and a count of domain subtype sequences of more than 3. The y-axis ranges in sequence logo figures all are 0~4.4 bits. The numbers of each C domain subtype are labeled at the end. For clarification, only motifs that have prevalent length are used in plotting. The actual total C domain number used in the figure for the specific motif is shown in the title. There are some gaps at both ends of the sequence for alignment. In motif C5, there are some interior gaps labeled in red to align with motif C5 of other subtypes. (PNG) [file pcbi.1011100.s017.png]

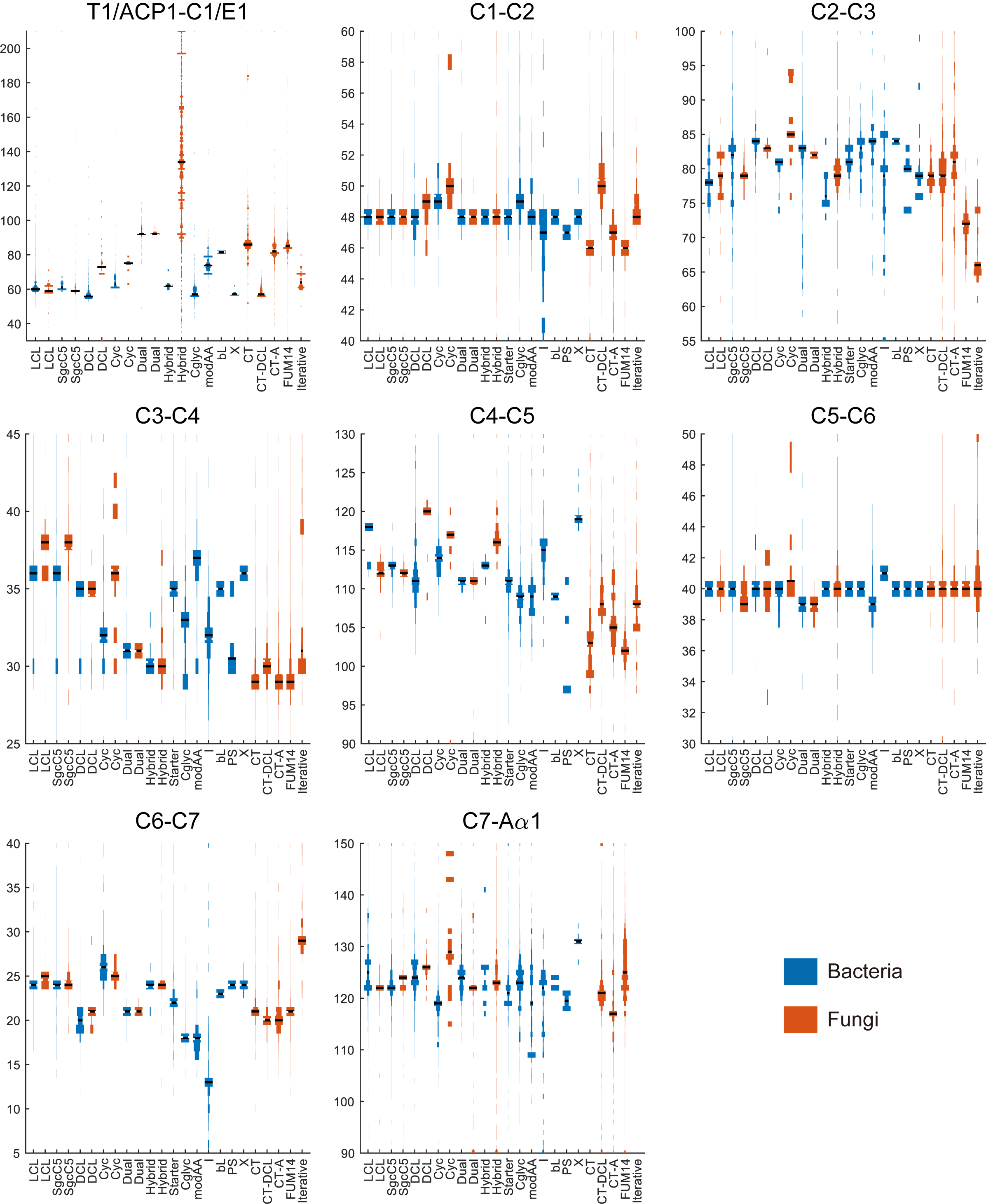

Supplement: S18 Fig — For comparison of intermotif length between different C domain subtypes and E domain, we chose the conserved positions which exist in all C domain and E domain as start and end of intermotif. T1/ACP1-C1/E1 ends before the conserved “Q” in C1 and E1 (the conserved “E” in LCL-A subtype). C1-C2 starts with the conserved “Q” in C1 (the conserved “E” in LCL-A subtype), and ends before the second conserved “R” in C2. C2-C3 starts before the second conserved “R” in C2 and ends before the conserved “D” in C3. C3-C4 starts before the conserved “D” in C3 and ends before the second conserved “Y” in C4. C4-C5 starts before the second conserved “Y” in C4 and ends before the conserved “G” in C5. C5-C6 starts before the conserved “G” in C5 and ends before the conserved “P” in C6. C6-C7 starts before the conserved “P” in C6 and ends before the conserved “F” in C7 (the conserved “F” in LCL). C7-Aα1 starts before the conserved “F” in C7 (the conserved “F” in LCL) and ends before Aα1. Sequence numbers of intermotifs (C1-C2, C2-C3, C3-C4, C4-C5, C5-C6, C6-C7) are 77,152 in bacteria and 34,269 in fungi. Sequence numbers of intermotifs (actually interdomain) in bacteria are 28,185 for T1-C1 and 33,176 for C7-Aα1 in LCL subtype C domain, 6,967 for E7-C1 and 6,752 for C7-Aα1 in DCL subtype C domain, 3,860 for T1-C1 and 4,495 for C7-Aα1 in Dual subtype C domain, 6,967 for T1-C1 and 6,752 for C7-Aα1 in starter subtype C domain and while they in fungi are 434 for T1-C1 and 616 for C7-Aα1 in LCL subtype C domain, 808 for E7-C1 and 1,931 for C7-Aα1 in DCL subtype C domain and 24 for T1-C1 and 21 for C7-Aα1 in Dual subtype C domain.S19 Fig. (PNG) [file pcbi.1011100.s018.png]

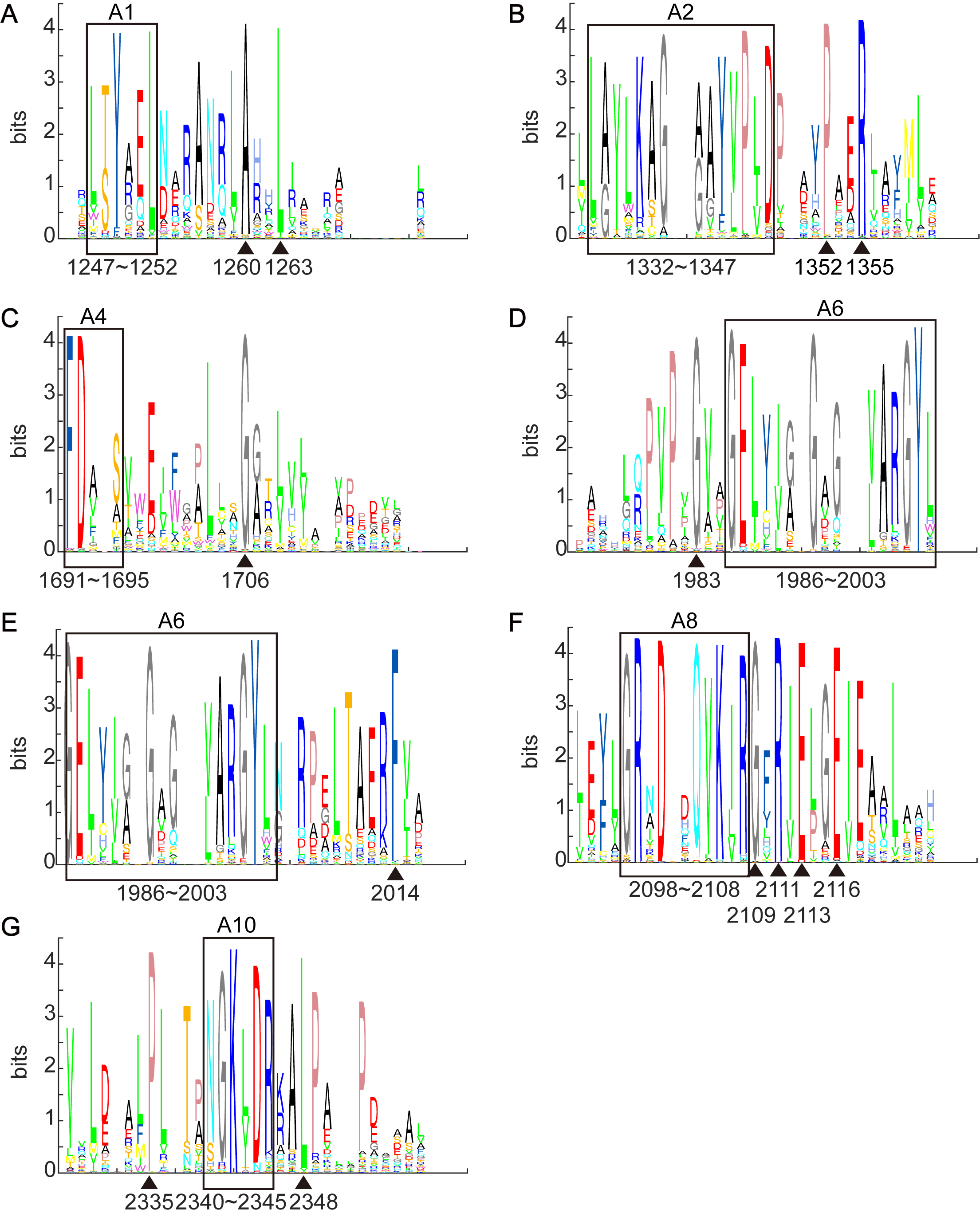

Supplement: S19 Fig — The black box shows known core motifs in the A domain. The black triangle shows highly conserved positions in multialignment from the 1,161 C+A+T NRPS sequences from MiBiG database. (PNG) [file pcbi.1011100.s019.png]

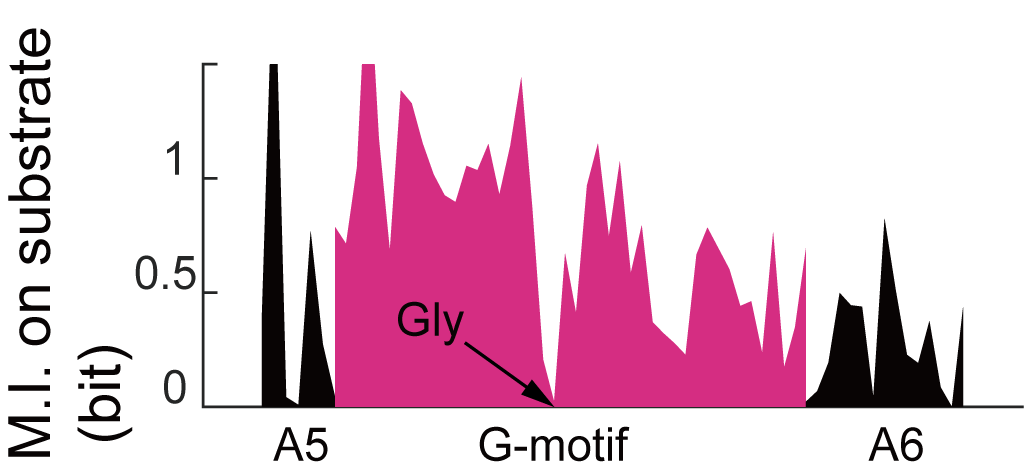

Supplement: S20 Fig — Same as that in the fourth panel of Fig 1B, but this plot focuses on regions between motif A5 and motif A6. The position of conserved Gly is indicated by the black arrow. (PNG) [file pcbi.1011100.s020.png]

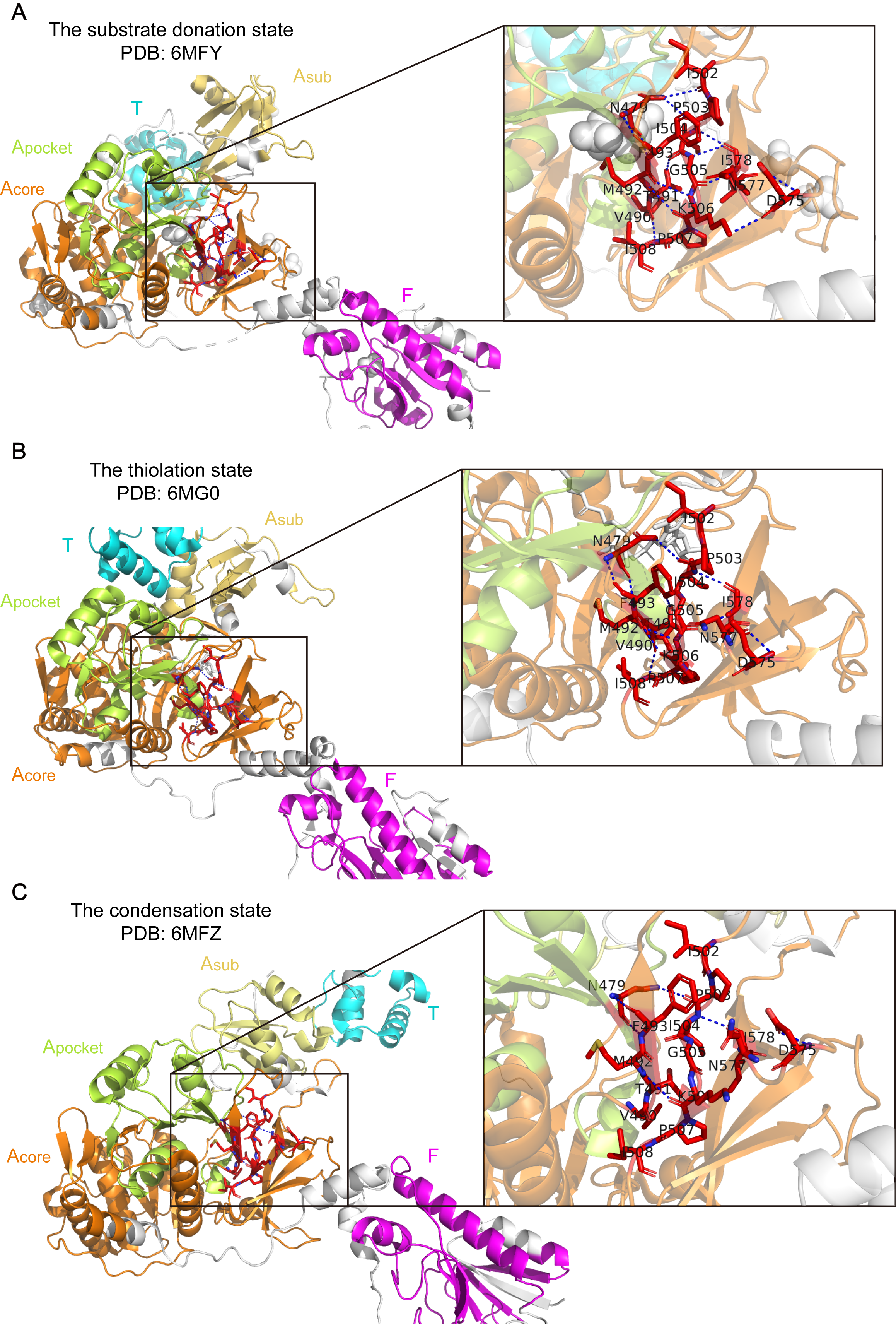

Supplement: S21 Fig — Related to Fig 3B–3D. The view zoomed in toward the region near the G-motif emphasized by red sticks. Hydrogen bonds were shown in the blue dashed-line. Different domains marked by different colors (F: formylation domain, colored by magenta; T: thiolation domain, colored by cyan; A: adenylation domain, Acore (orange) covers A1-A8 of A domain, Asub (yellow) covers A9-A10, Apocket (yellow green) covers A3-A6 of A domain). (PNG) [file pcbi.1011100.s021.png]

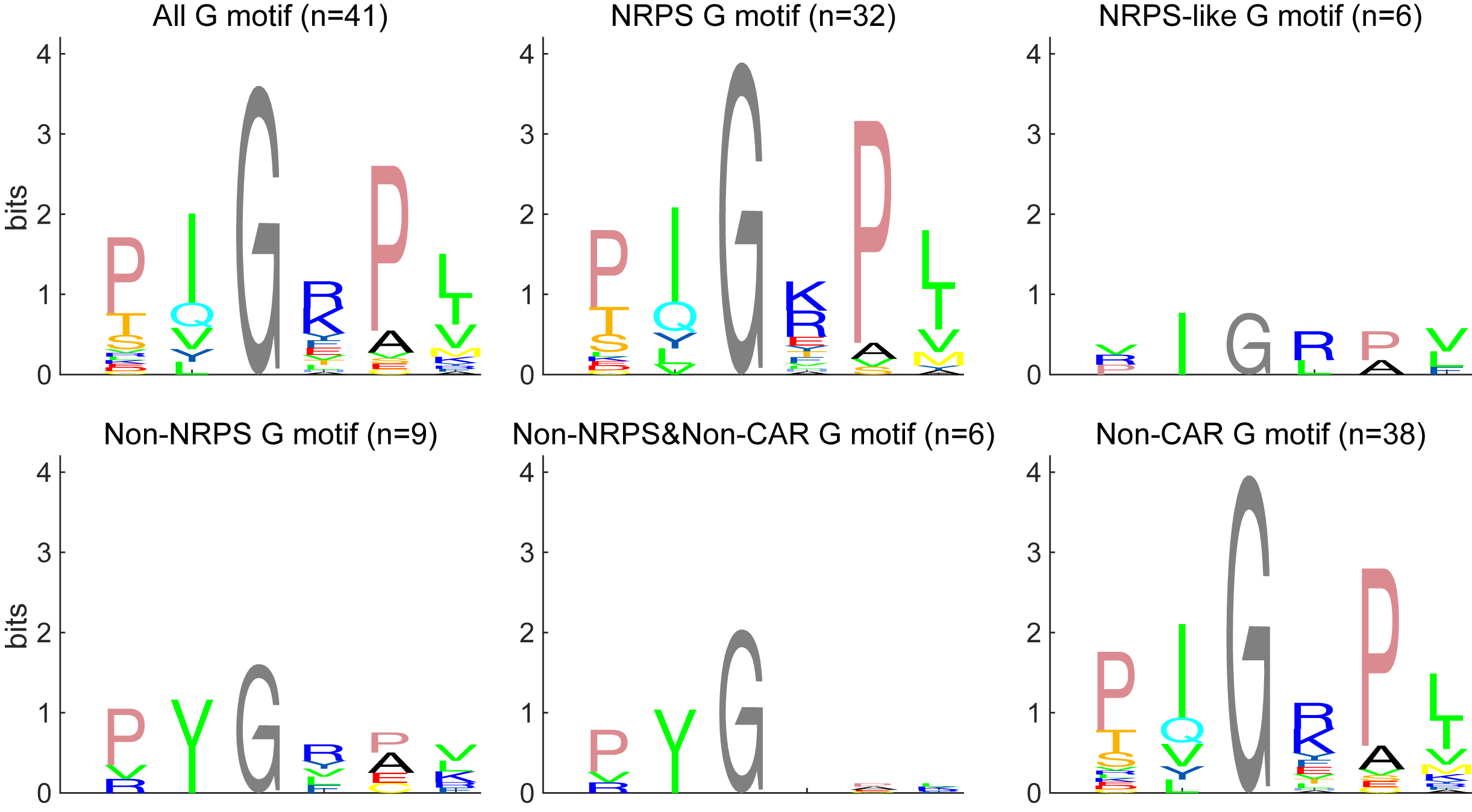

Supplement: S22 Fig — Non-NRPS means A domains from these proteins which aren’t NRPS. Non-CAR means A domains from these proteins which aren’t CAR. Non-NRPS&Non-CAR means A domains from these proteins which aren’t NRPS or CAR. (PNG) [file pcbi.1011100.s022.png]

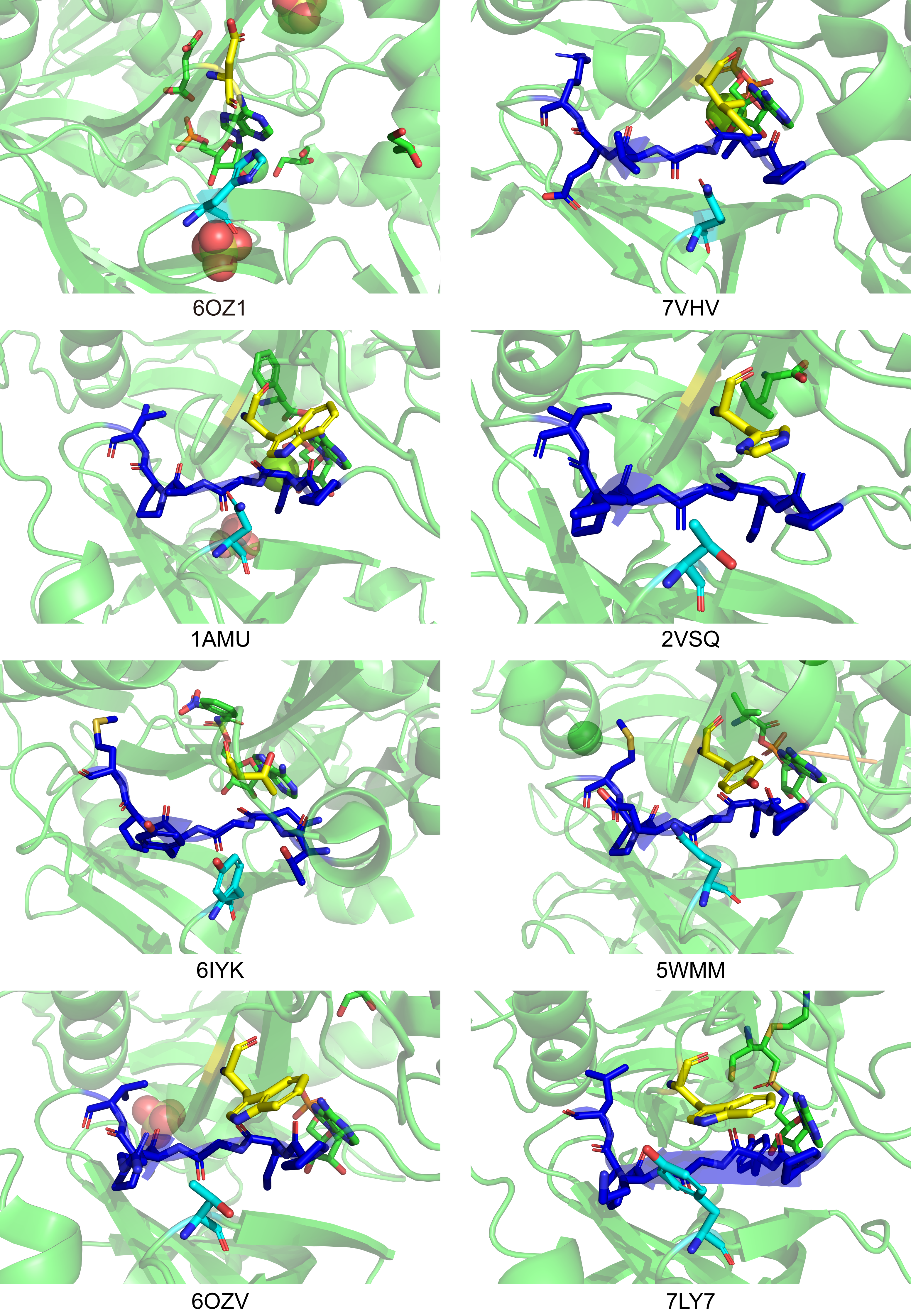

Supplement: S23 Fig — From the first to the 8-th figure, the known structures are CAR protein (PDB: 6OZ1), DltA (PDB: 7VHV), and NRPS (PDB: 1AMU, 2VSQ, 6IYK, 5WMM, 6OZV, and 7LY7). Residues in G-motif were marked by blue. The equivalent N397 and S491 were marked by yellow and cyan. The ligand molecule was marked in green. The PDB IDs of proteins are shown below. Only the CAR protein doesn’t contain the G-motif, but it still has equivalents of N397 and S491. G-motif is in close proximity to the adenylate part of the ligand, suggesting a potential gatekeeper role. (PNG) [file pcbi.1011100.s023.png]

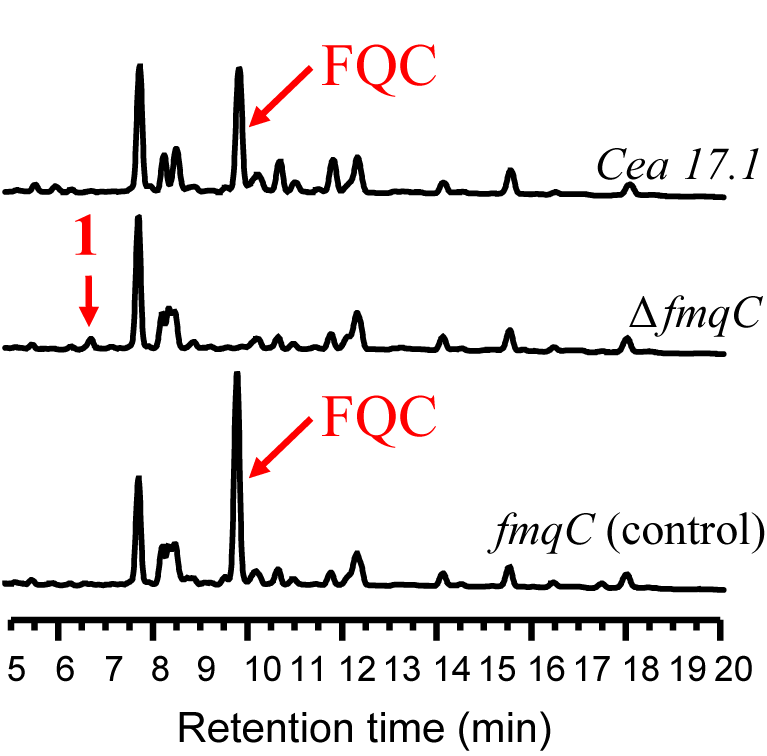

Supplement: S24 Fig — Wild type (Cea17.2, first row), ΔfmqC (second row) and the control fmqC (third row). (PNG) [file pcbi.1011100.s024.png]

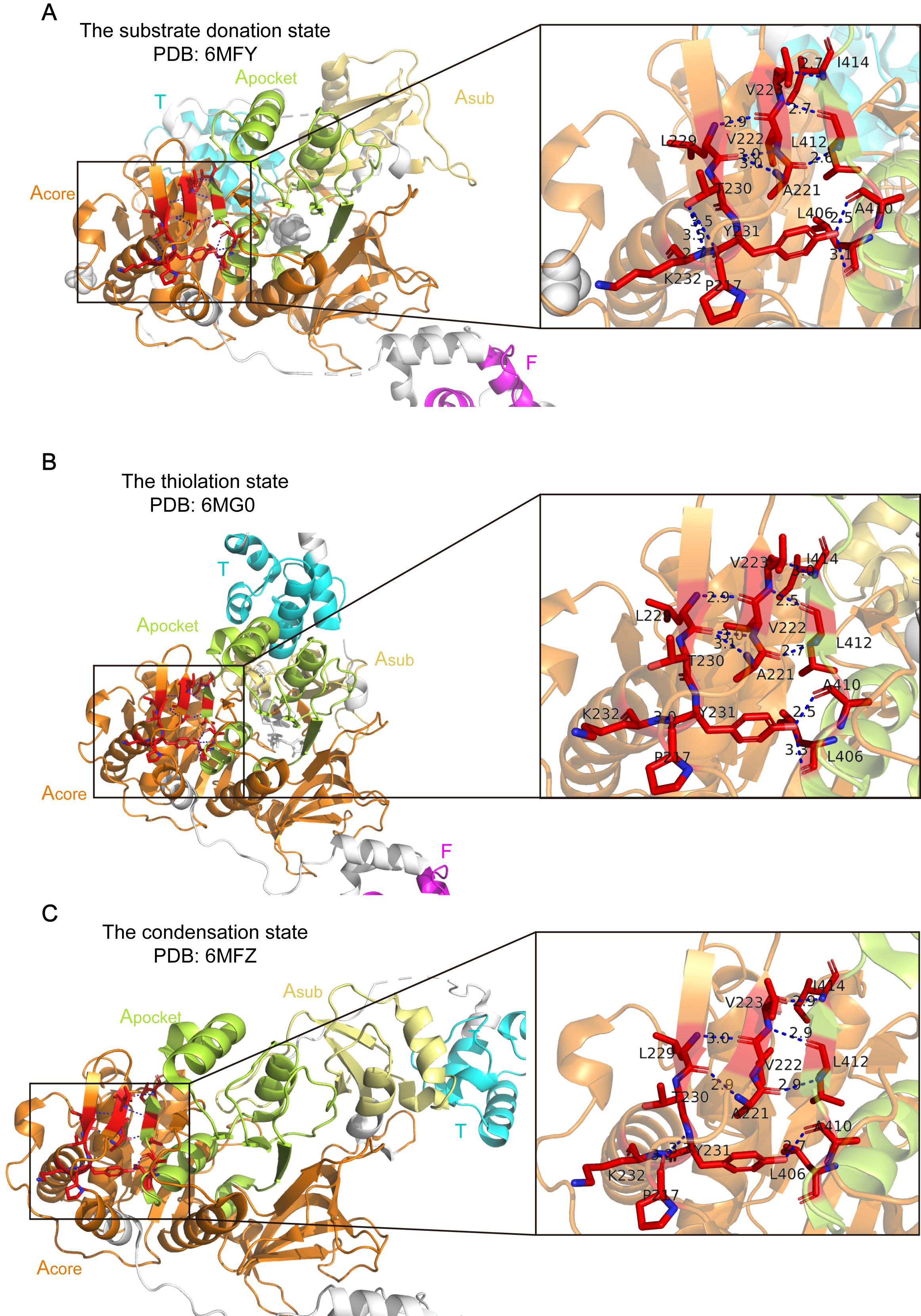

Supplement: S25 Fig — Similar to S21, but for the Aα1 motif. The view zoomed in towards the region near the Aα1 motif emphasized by red sticks. (PNG) [file pcbi.1011100.s025.png]

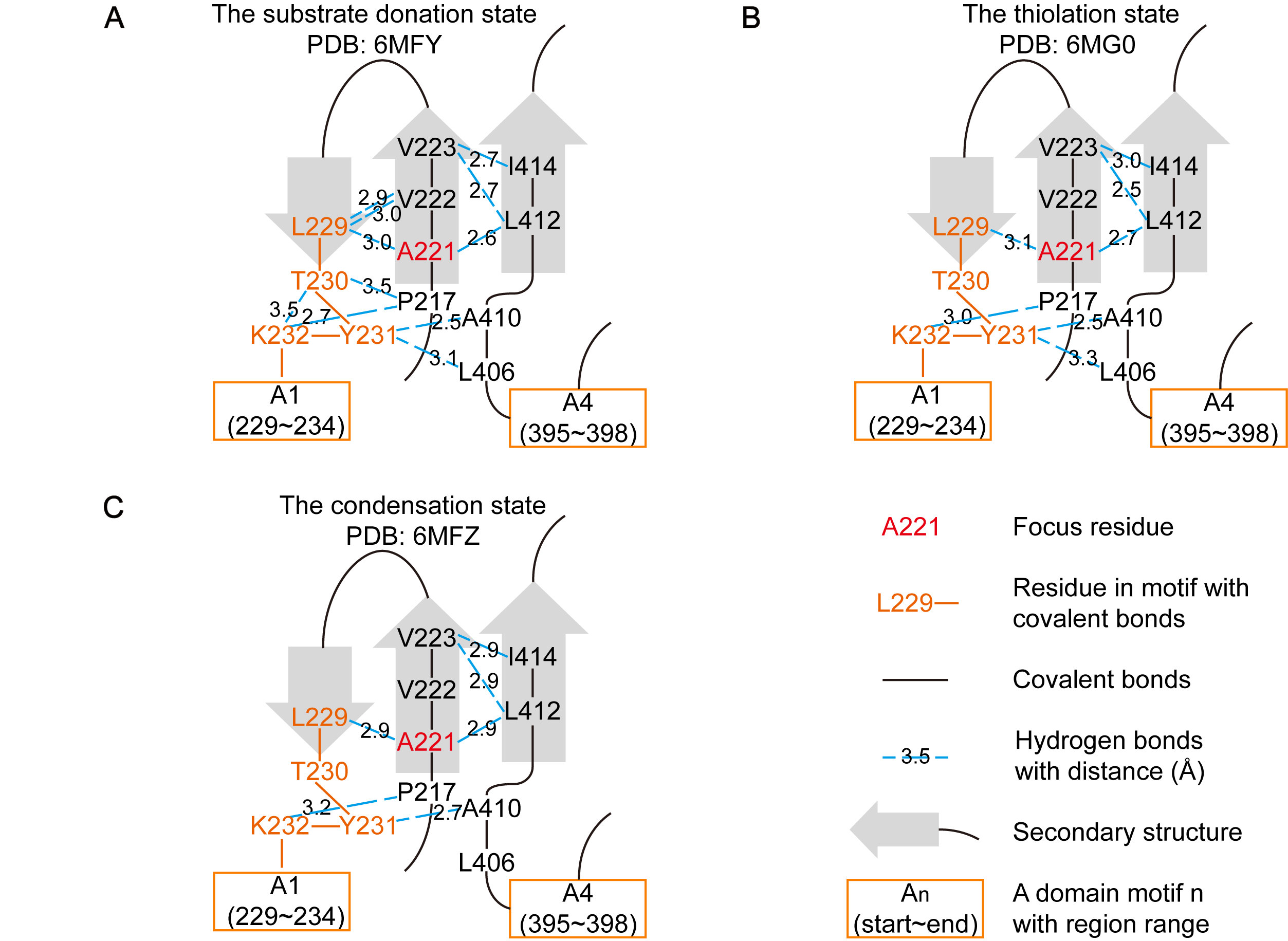

Supplement: S26 Fig — Related to S25. Similar to Fig 3B, but shows chemical interactions and secondary structures surrounding the Aα1 motif at the substrate donation state (A), the thiolation state (B) and the condensation state (C). Of note, in these residues, only T230 and Y231 use the hydroxyl group in the side chain to form hydrogen bonds. Other hydrogen bonds, on the other hand, are formed by the common α-carboxyl group and α-amino group in the main chains. (PNG) [file pcbi.1011100.s026.png]

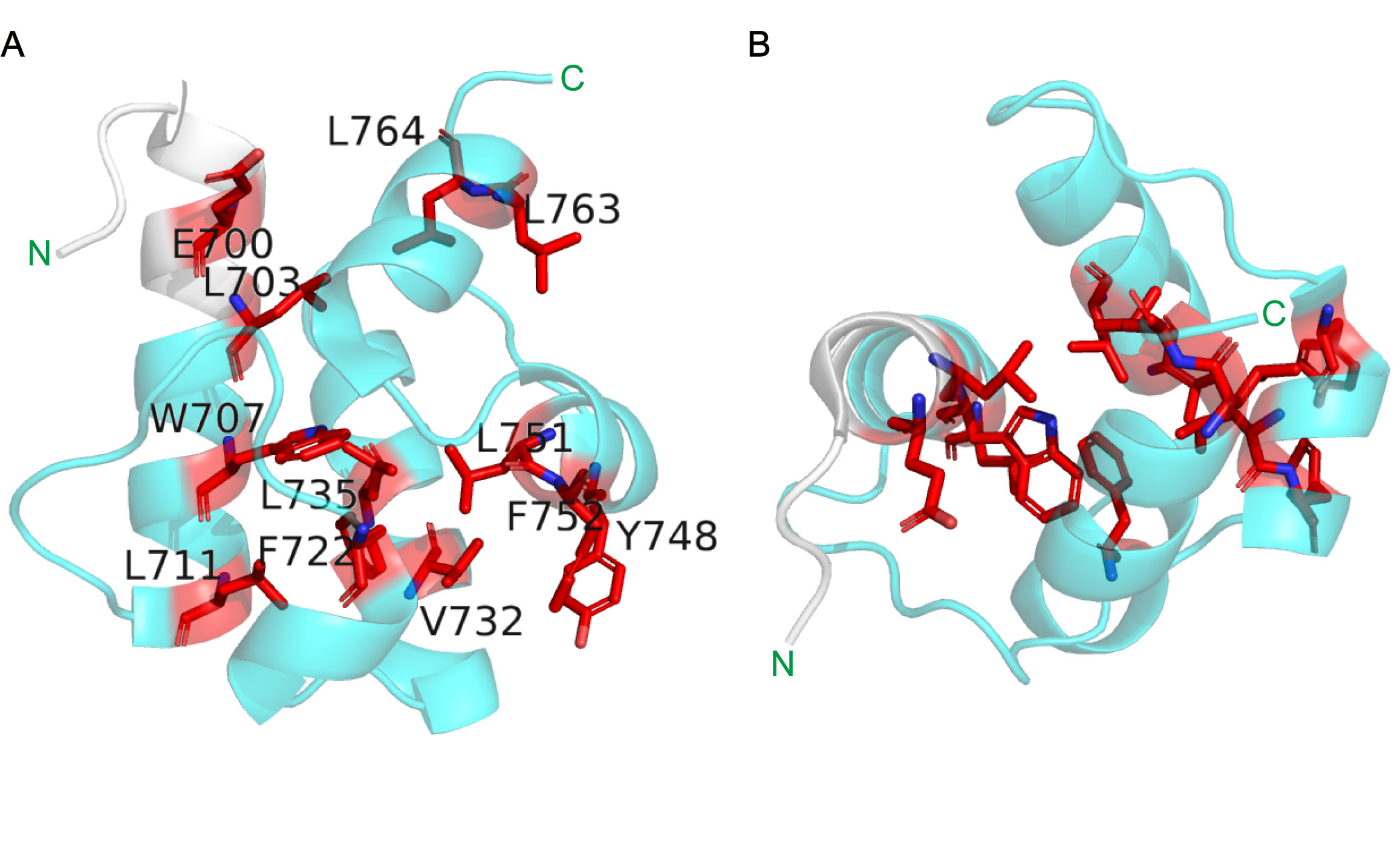

Supplement: S27 Fig — The structure is obtained from LgrA in the condensation state (PDB: 6MFZ). Cyan color shows the T domain defined by Pfam. A small white region isn’t covered by Pfam, although it is visually one part of the first helix of T domain. N-terminal and C-terminal were marked by green texts. Conserved resides were marked by red sticks with their one letter labels shown. Reside labels in the top view were hidden for visual clearness. A. Side view of T domain. B. Top view of T domain. (PNG) [file pcbi.1011100.s027.png]

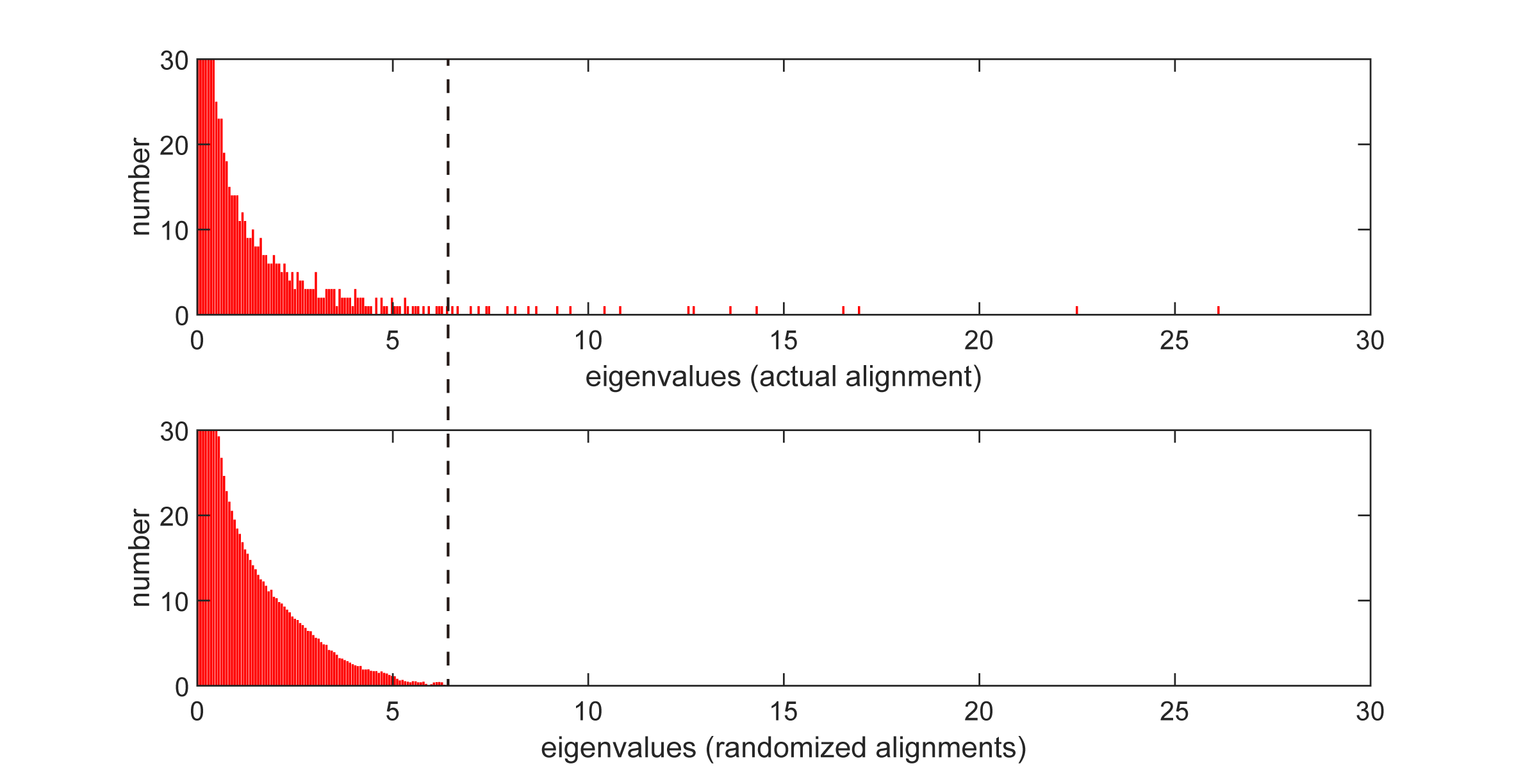

Supplement: S28 Fig — Eigenvalue spectra for the SCA matrix corresponding to the 1,161 C+A+T modules (top panel) and for 100 trials of randomizing sequences alignment (bottom panel). The randomization process scrambles the order of amino acids in each alignment column independently, which did not change amino acid frequencies at positions. The black dashed-line marked the maximum of eigenvalues from randomized alignments. This analysis shows that only a small part of the spectrum (26 out of 2560 total eigenvalues) is significant given sample size. (PNG) [file pcbi.1011100.s028.png]

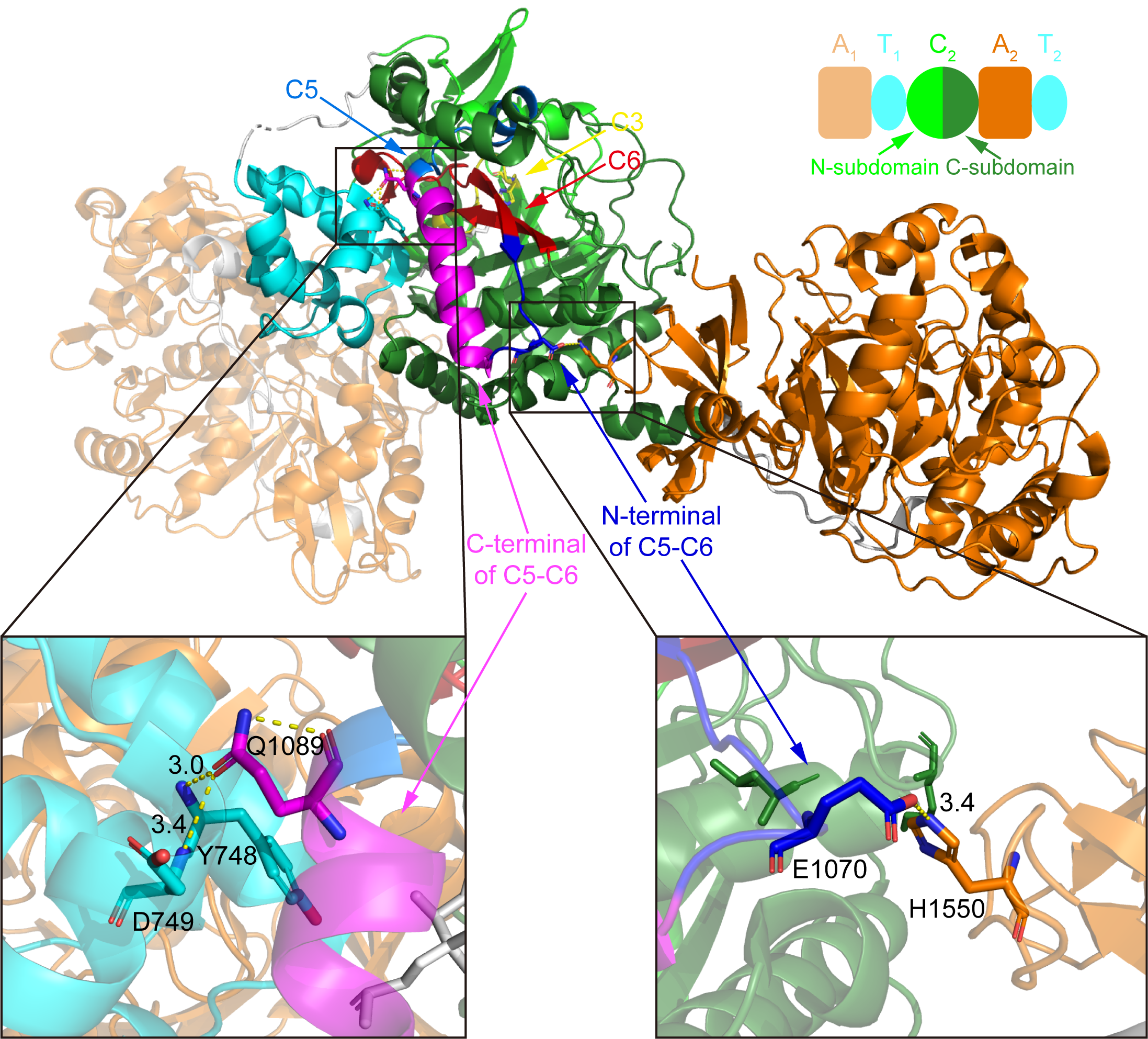

Supplement: S29 Fig — The formylation domain in the first module (F1) of LgrA is hidden for visual clearness. The colors of each domain are noted at the top of the figure. The C domain is split into the N-terminal subdomain (N-subdomain, covering C1-C4) and the C-terminal subdomain (C-subdomain, covering C5-C7), referring to previous research (PMID: 23756159). The active site histidine (the second histidine in C3 motif HHxxxD), the residues in C5-C6 intermotif interacting with T domain or A domain, and the residues in T domain and A domain interacted by these residues are shown as stick format. Hydrogen bonds were shown in yellow dashed-line with distances by black. (PNG) [file pcbi.1011100.s029.png]

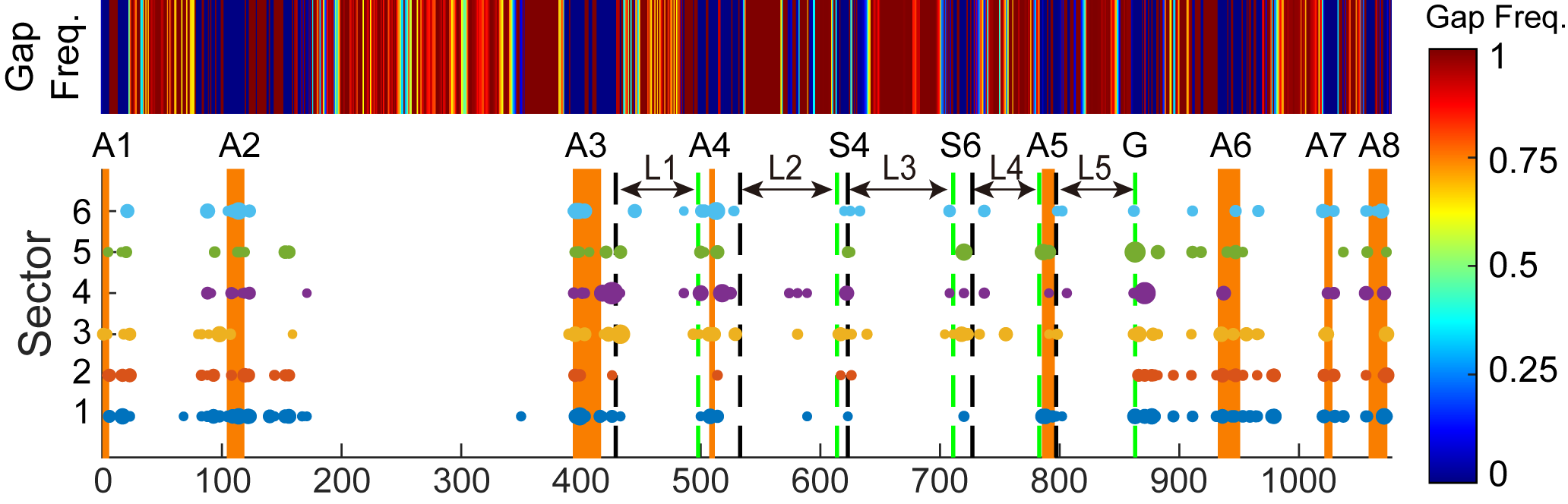

Supplement: S31 Fig — Related to Fig 6A, upper panel shows the gap frequency in the MSA of 2,636 A domains. Some residues in the substrate-related sectors were found in the highly variable loop regions (L1-L5). These regions contain high numbers of gaps in MSA, and are usually loops in the structure. (PNG) [file pcbi.1011100.s031.png]

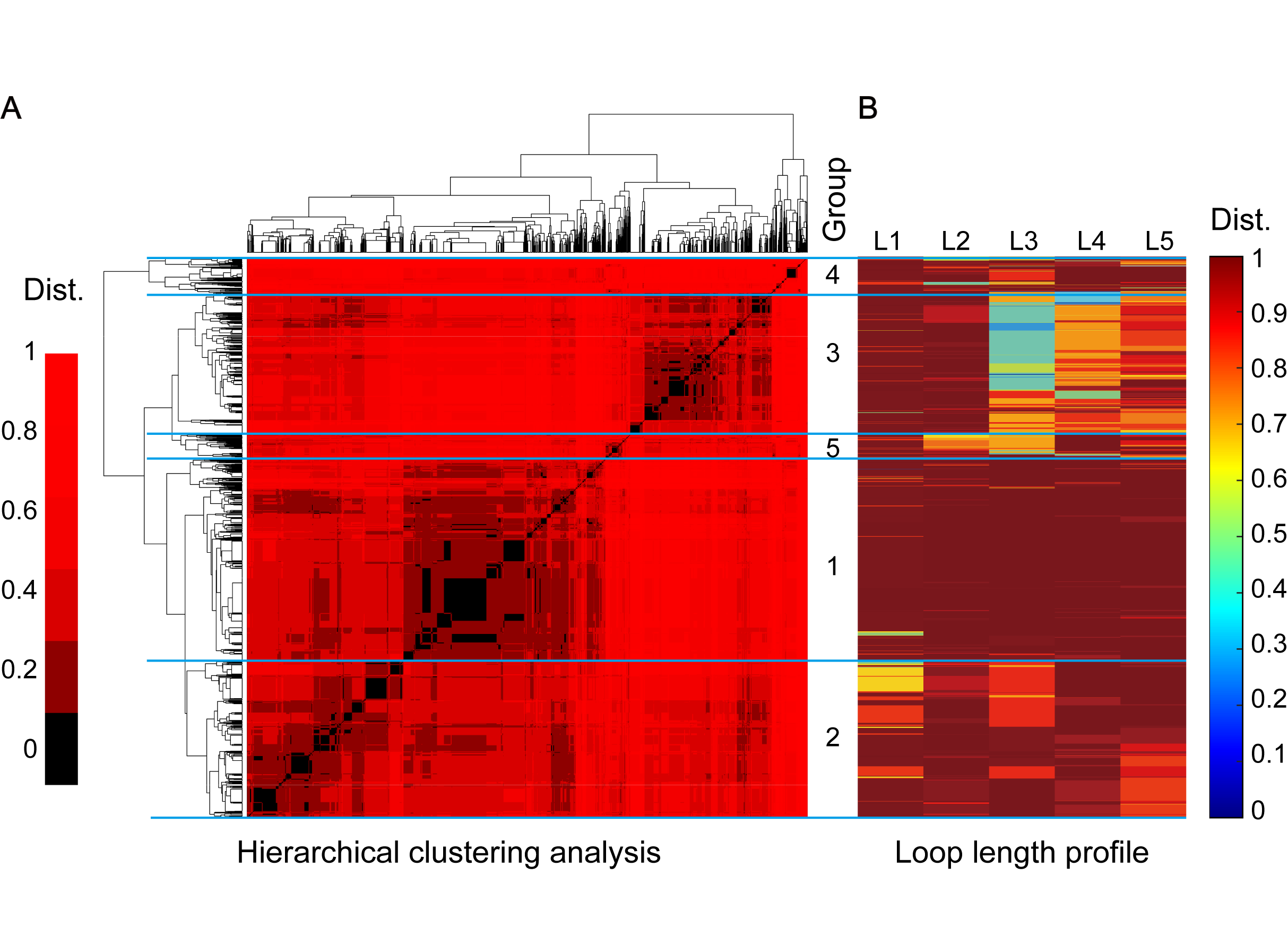

Supplement: S32 Fig — A. Hierarchical clustering of the A domains based on the Euclidean distances of their lengths in five loops. A domains were categorized into five groups based on their loop-length vectors. For visual clearness, in calculation, the Euclidean distances which are more than 12 are set as 12 before normalizing. B. Loop length profiles of five groups shown in A. More details in Method. (PNG) [file pcbi.1011100.s032.png]

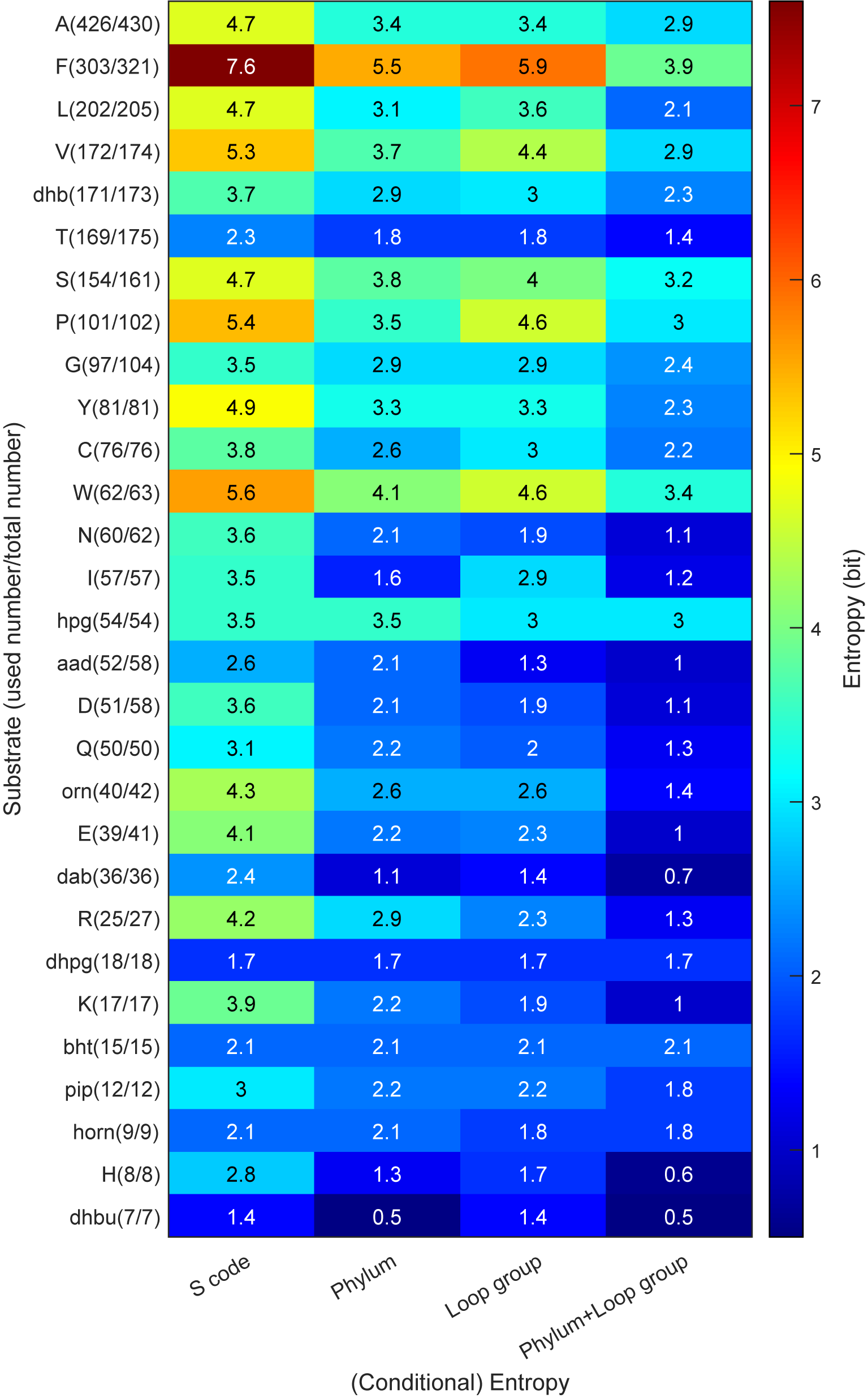

Supplement: S33 Fig — The first column shows the entropy of the specificity-conferring code for different substrates. The proteinogenic amino acids are named according to the standard amino acid one-letter code. Abbreviations of non-proteinogenic amino acid substrate: aad = 2-amino-adipic-acid, bht = beta-hydroxy-tyrosine, dab = diaminobutyric acid, dhb = 2,3-dihydroxy-benzoic acid, dhbu = 2,3-dehydroaminobutyric acid, dhpg = 3,5-dihydroxy-phenyl-glycin, horn = hydroxy-L-ornithine, hpg = 4-hydoxy-phenyl-glycine, orn = ornithine and pip = pipecolic acid. Only A domains from 5 main phylum are used to calculate entropy (2564/2623 = 97.8% sequences). The sequence number of each substrate was marked in the bracket after substrate names on the y labels (the first is the number used in the calculation of entropy, and the second is the total number of this substrate in our datasets). Second to fourth columns show the conditional entropy of the specificity-conferring code given information about the phylum, the loop group, and the phylum with the loop group, respectively. Information from the phylum and the loop group could both reduce the uncertainty of the specificity-conferring code, and they together could further reduce the uncertainty. (PNG) [file pcbi.1011100.s033.png]

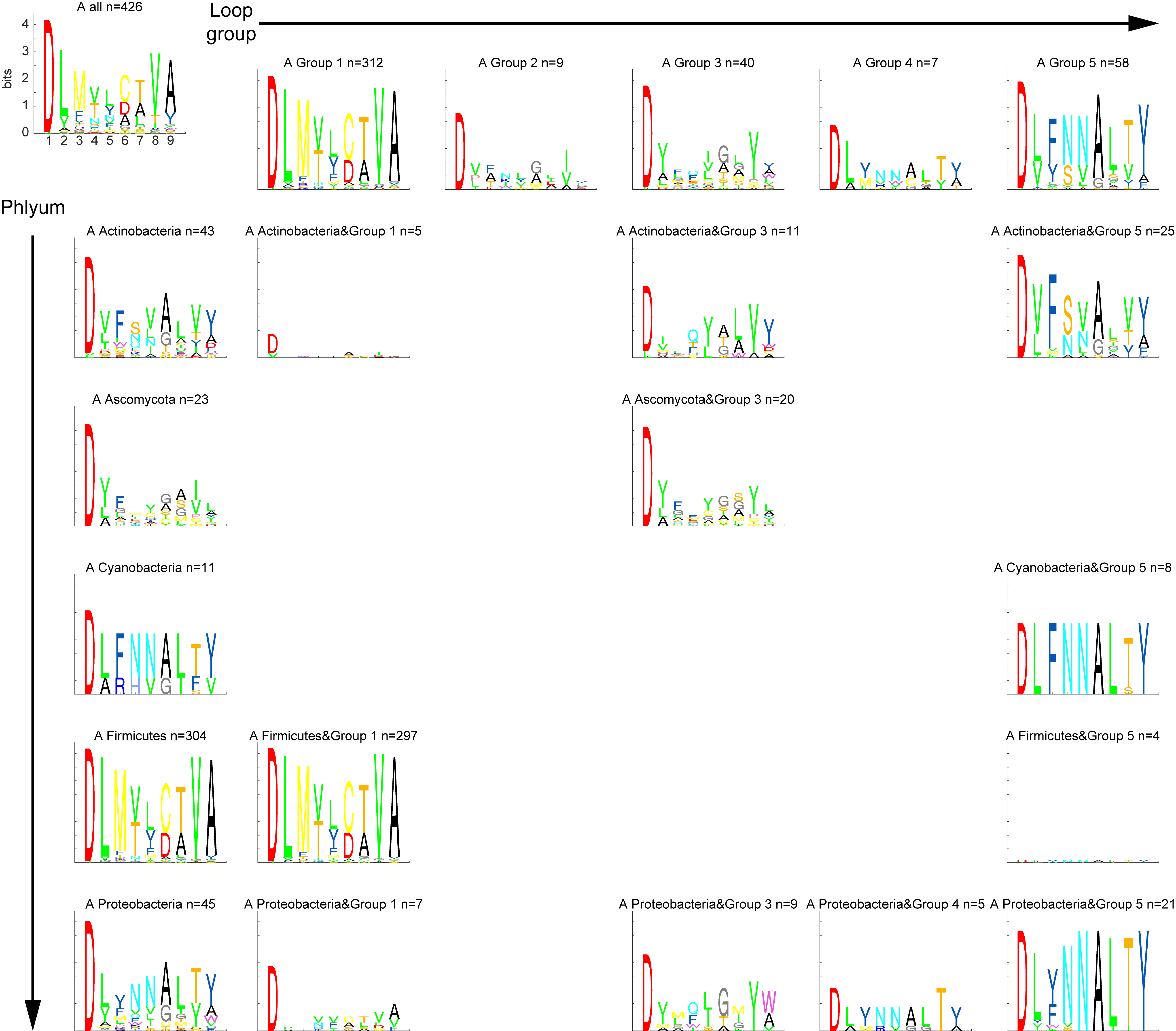

Supplement: S34 Fig — 9 of 10 the specificity-conferring code are displayed. The last one is conserved lysine (K) in the A10 motif. It wasn’t shown because our A domain sequences only cover A1-A8. Sequence logo will not be plotted, if the number of sequences is less than 3. Substrate abbreviation: A = alanine. (PNG) [file pcbi.1011100.s034.png]

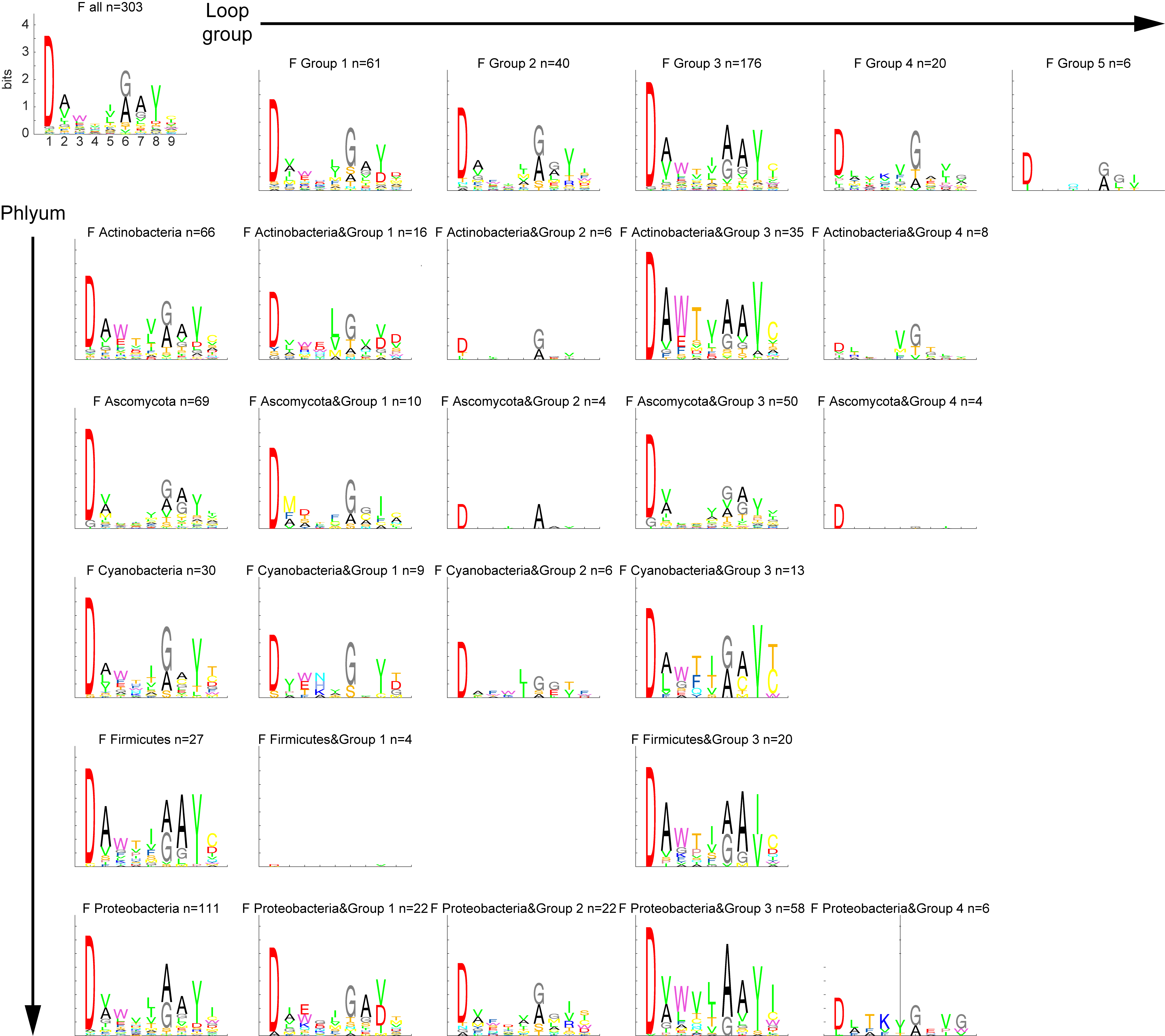

Supplement: S35 Fig — Similar to S34, but for substrate phenylalanine (F). (PNG) [file pcbi.1011100.s035.png]

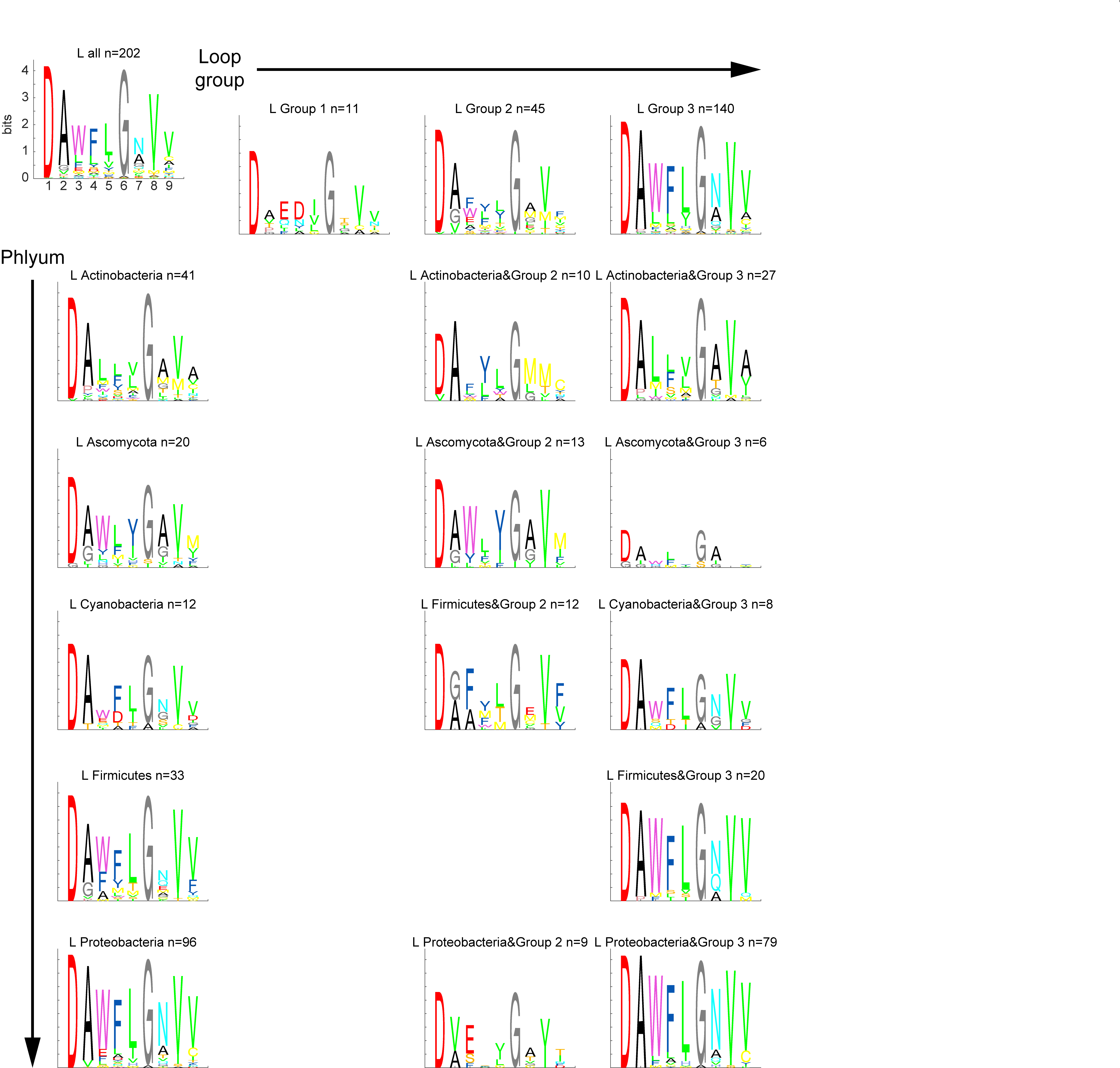

Supplement: S36 Fig — Similar to S34, but for substrate leucine (L). (PNG) [file pcbi.1011100.s036.png]

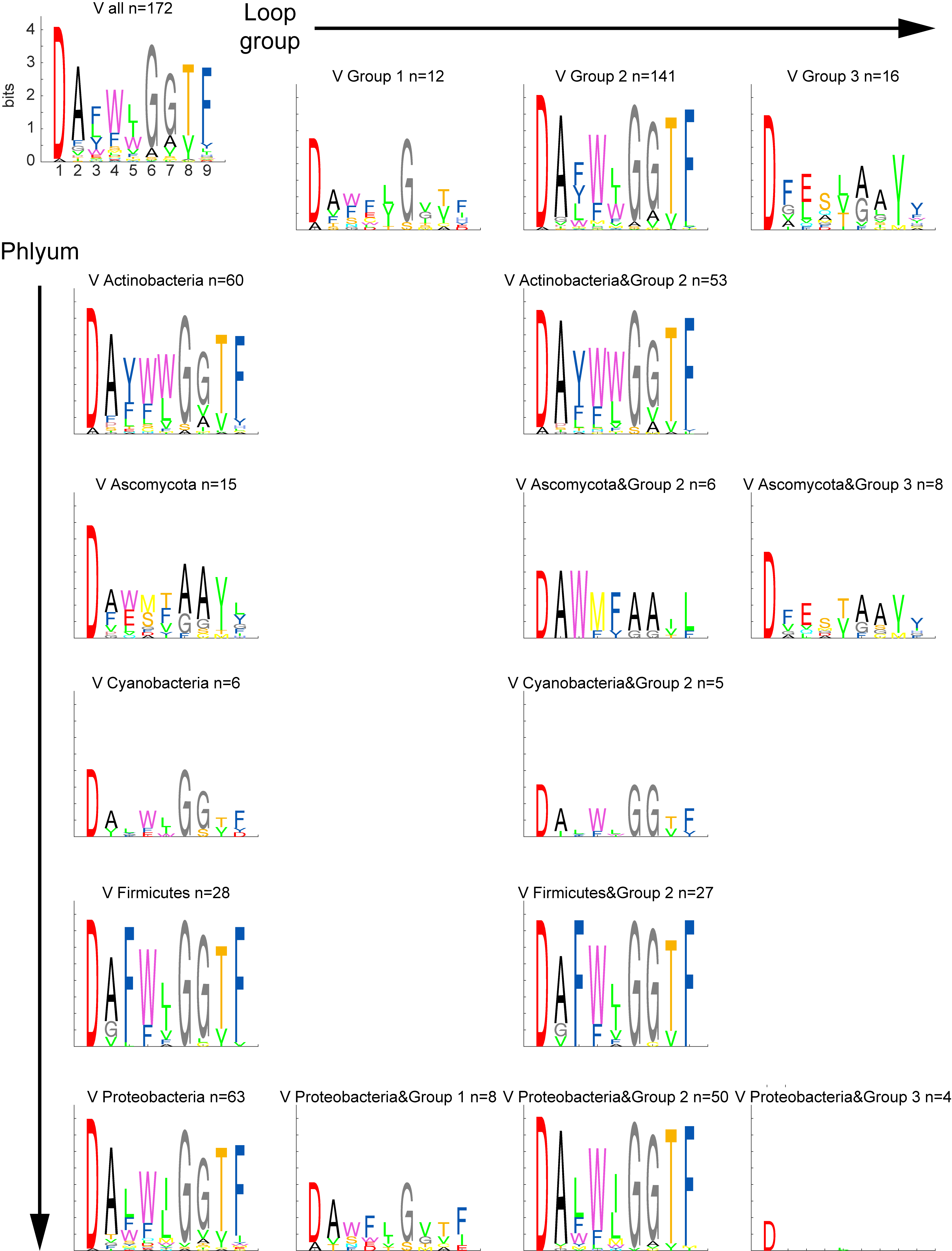

Supplement: S37 Fig — Similar to S34, but for substrate valine (V). (PNG) [file pcbi.1011100.s037.png]

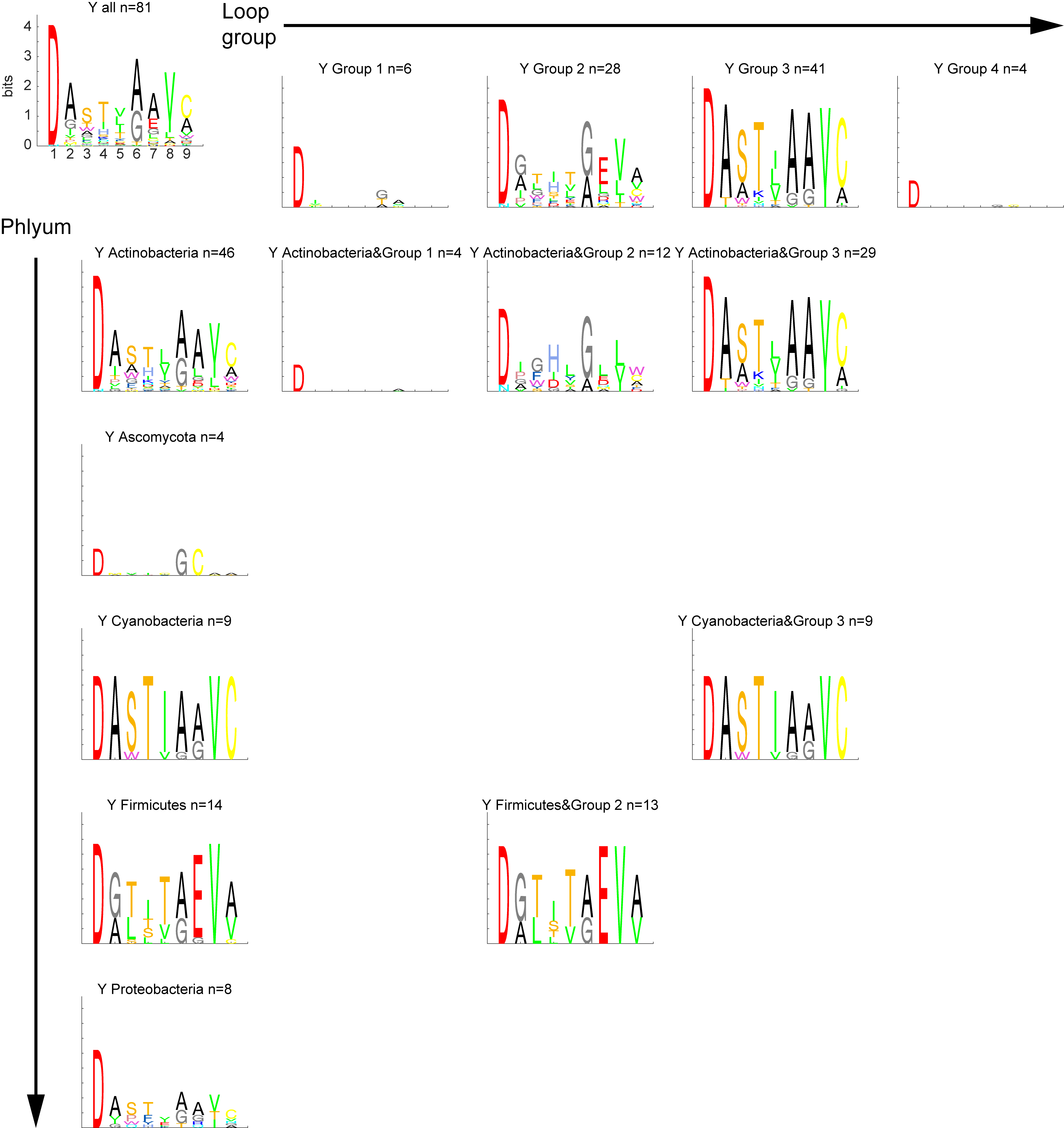

Supplement: S38 Fig — Similar to S34, but for substrate tyrosine (Y). (PNG) [file pcbi.1011100.s038.png]

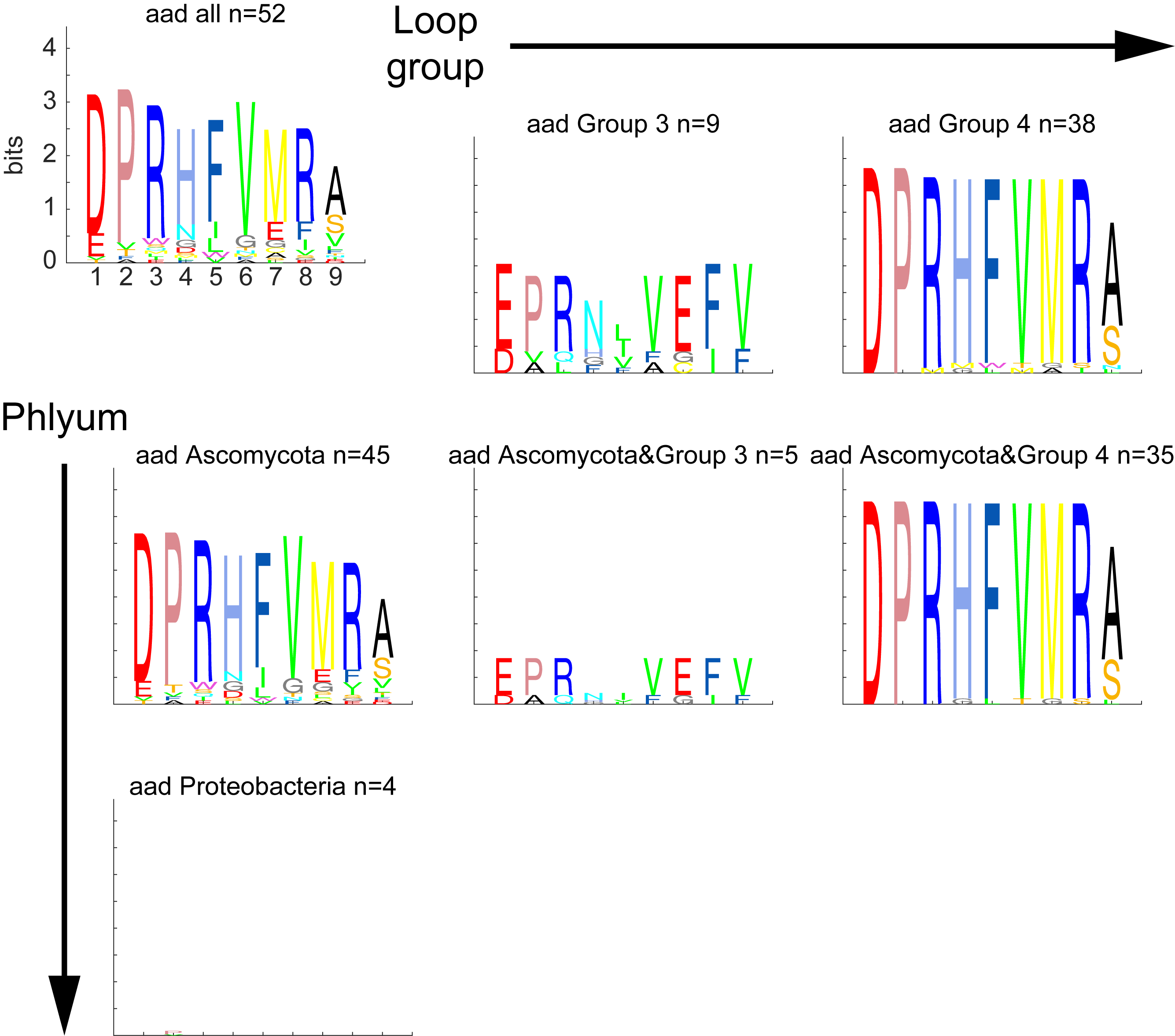

Supplement: S39 Fig — Similar to S34, but for substrate 2-amino-adipic-acid (aad). (PNG) [file pcbi.1011100.s039.png]

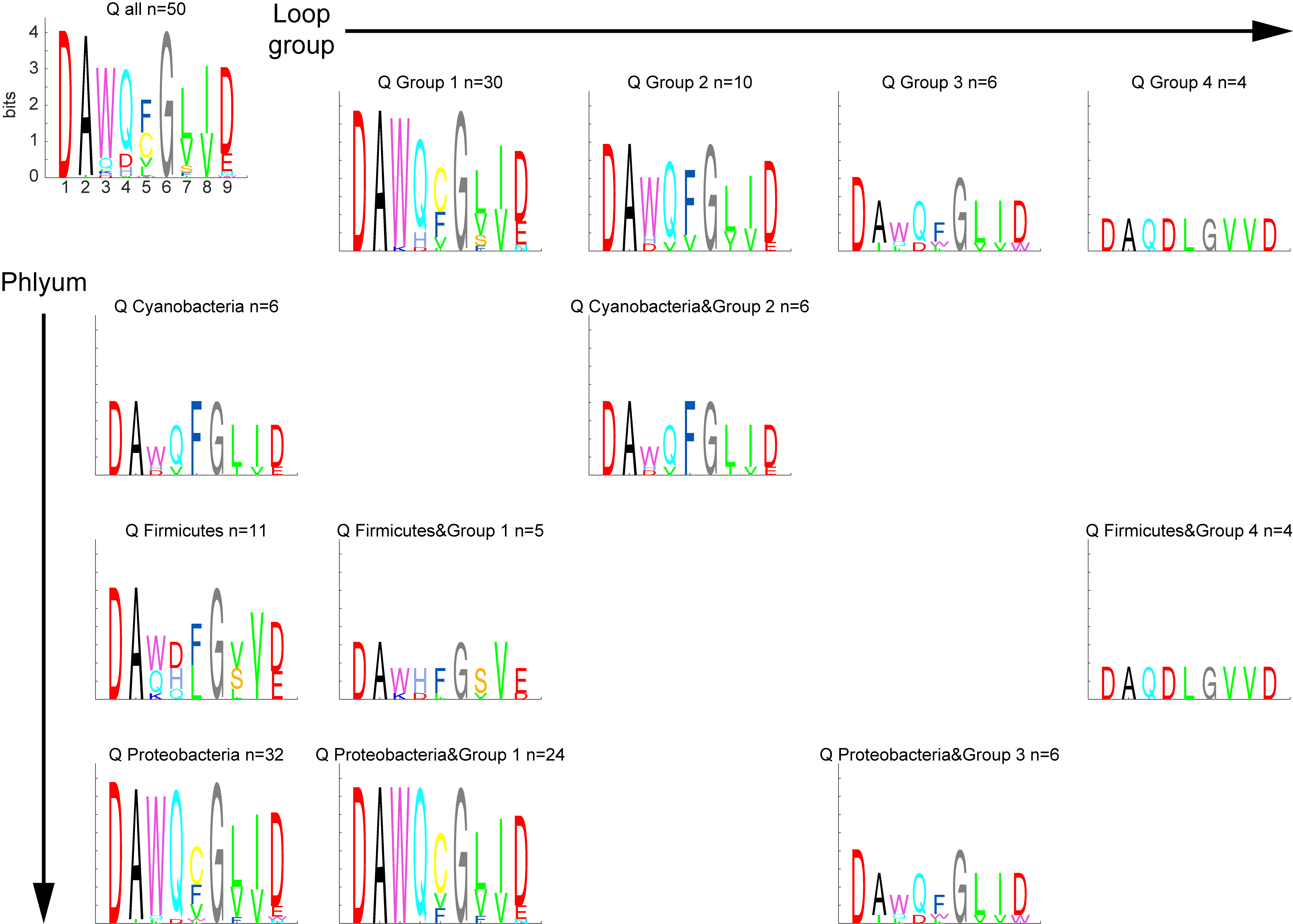

Supplement: S40 Fig — Similar to S34, but for substrate glutamine (Q). (PNG) [file pcbi.1011100.s040.png]

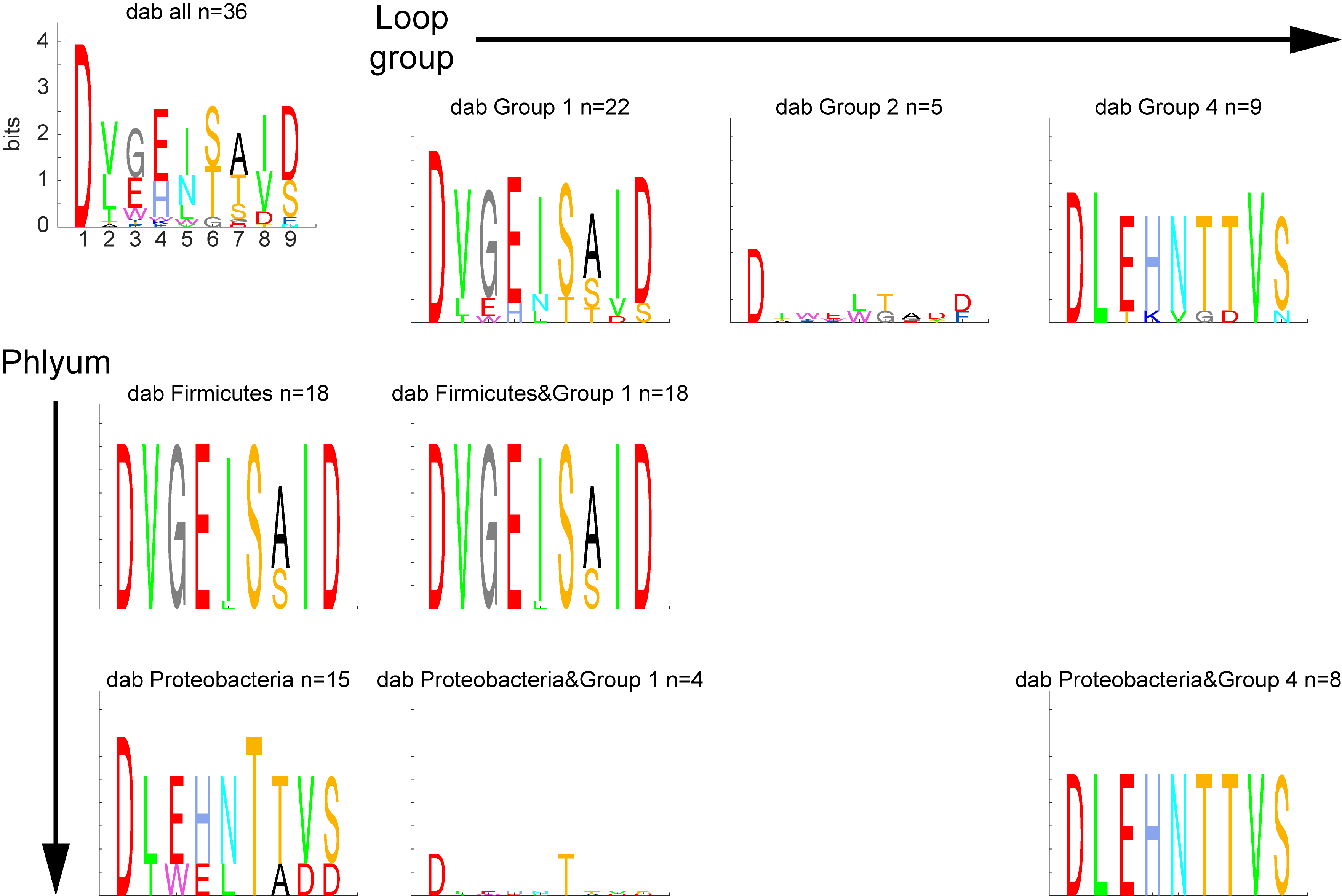

Supplement: S41 Fig — Similar to S34, but for substrate diaminobutyric acid (dab). (PNG) [file pcbi.1011100.s041.png]

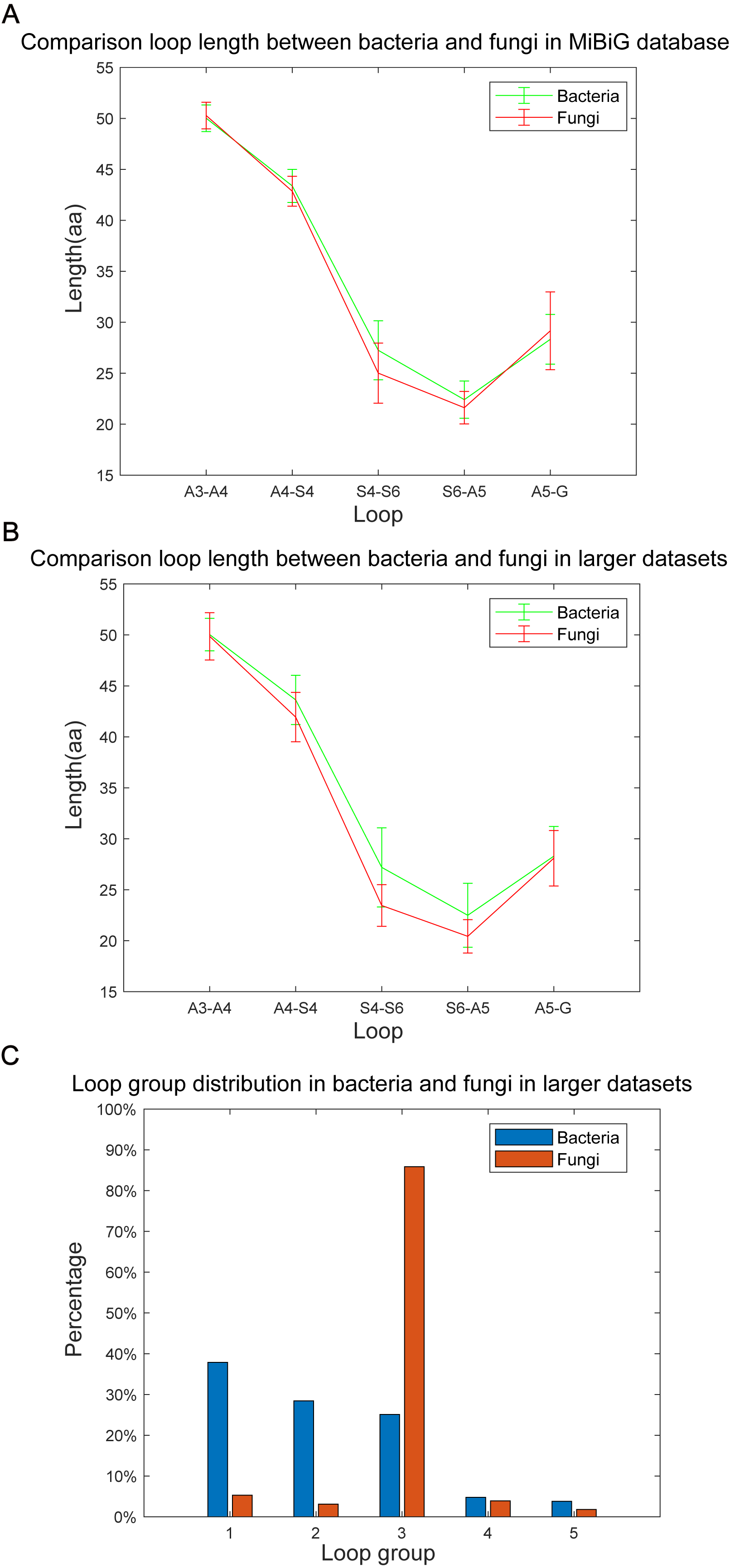

Supplement: S42 Fig — A. Comparison loop length between bacteria and fungi in the MiBiG database. The numbers of A domains are 2,370 and 215 for bacteria and fungi, respectively. B. Comparison loop length between bacteria and fungi in the larger dataset. The numbers of A domains are 61,494 and 4,484 for bacteria and fungi, respectively. C. Loop group distribution in bacteria and fungi in the larger datasets. The numbers of A domains are the same as B. Their loop groups are predicted as the closest one loop group in the MiBiG database by calculating Euclidean distance. A small amount of data (<5%) is not counted because they are the same distance from multiple loop groups. (PNG) [file pcbi.1011100.s042.png]

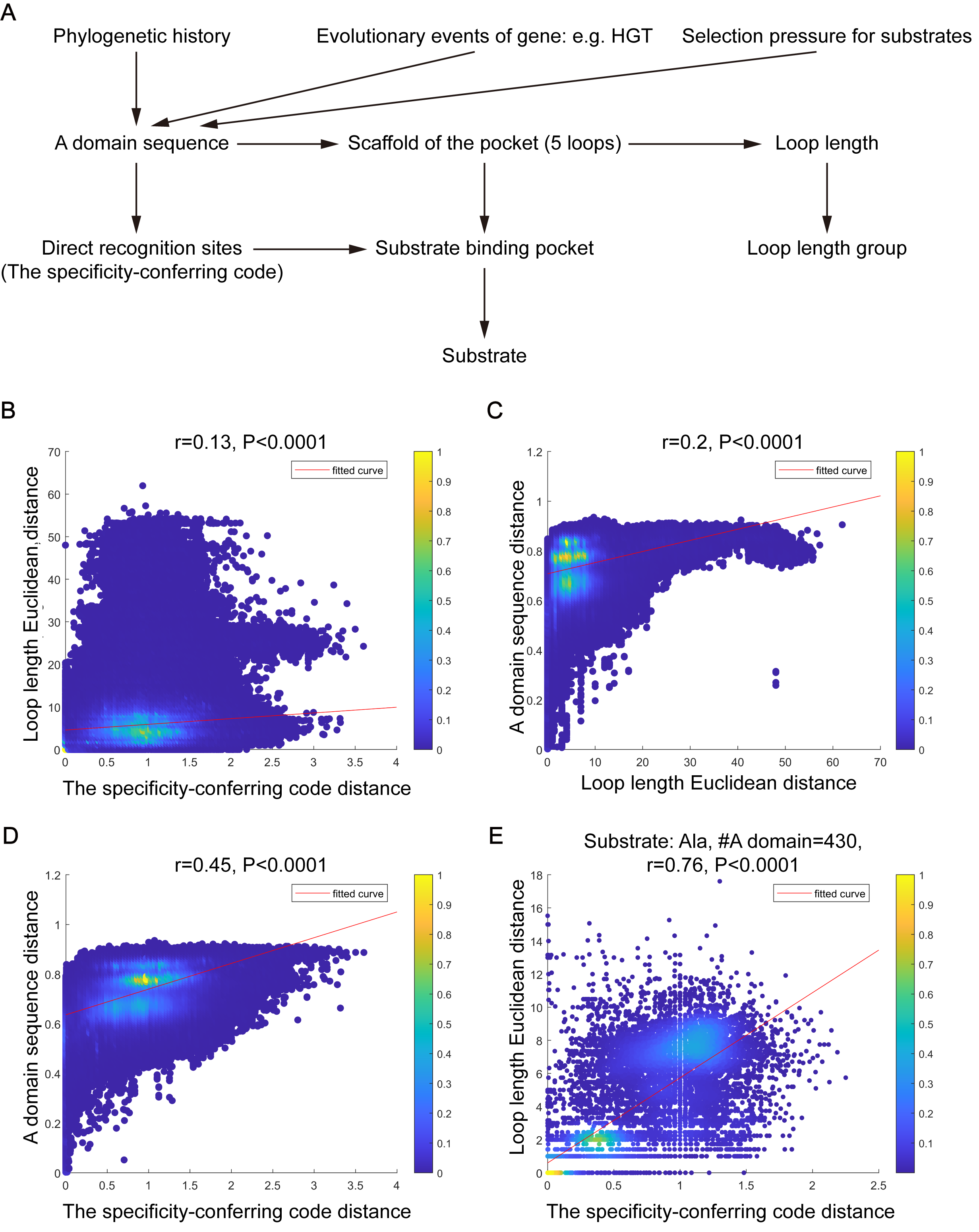

Supplement: S43 Fig — The specificity-conferring code distance used is alignment-score distance. A domain sequence distance used is p-distance. The loop length distance used is Euclidean distance. r is Pearson correlation coefficient. A. Causal diagram of A domain substrate specificity. B. Relationship between the specificity-conferring code distance and Loop length Euclidean distance. C. Relationship between Loop length Euclidean distance and A domain sequence distance. D. Relationship between the specificity-conferring code distance and A domain sequence distance. E. Relationship between the specificity-conferring code distance and Loop length Euclidean distance for A domains with substrate Ala. Similar to B, but for 430 A domains activating Ala as substrate. (PNG) [file pcbi.1011100.s043.png]

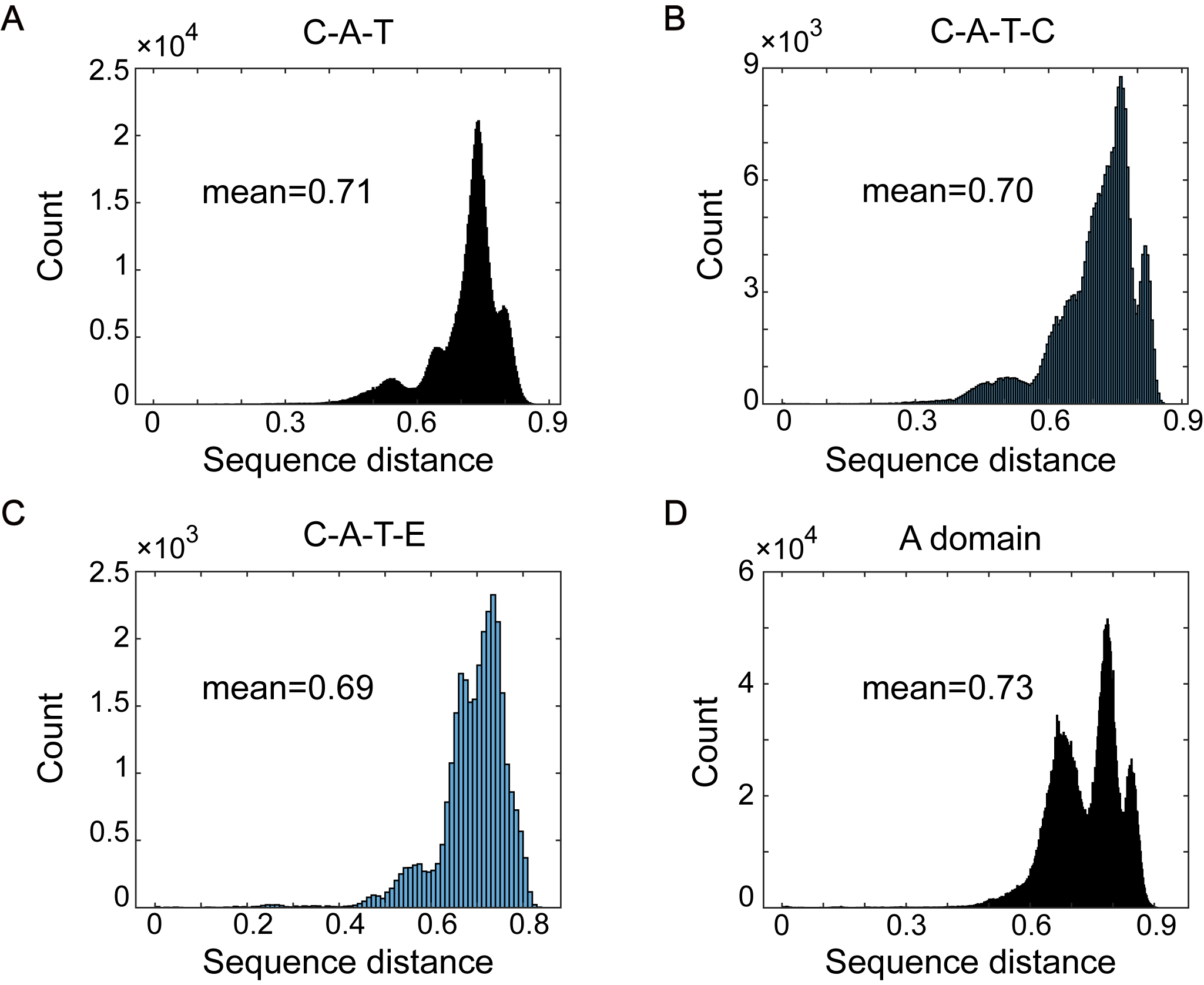

Supplement: S44 Fig — A. Pairwise distance distribution of 1,161 C+A+T NRPS sequences. The calculation was based on the p-distance, representing the fraction of amino acid being different after global alignment. B. Same as that in A, but for 685 C+A+T+C NRPS sequences. C. Same as that in A, but for 245 C+A+T+E NRPS sequences. D. Same as that in A, but for 2,636 A domain sequences. (PNG) [file pcbi.1011100.s044.png]

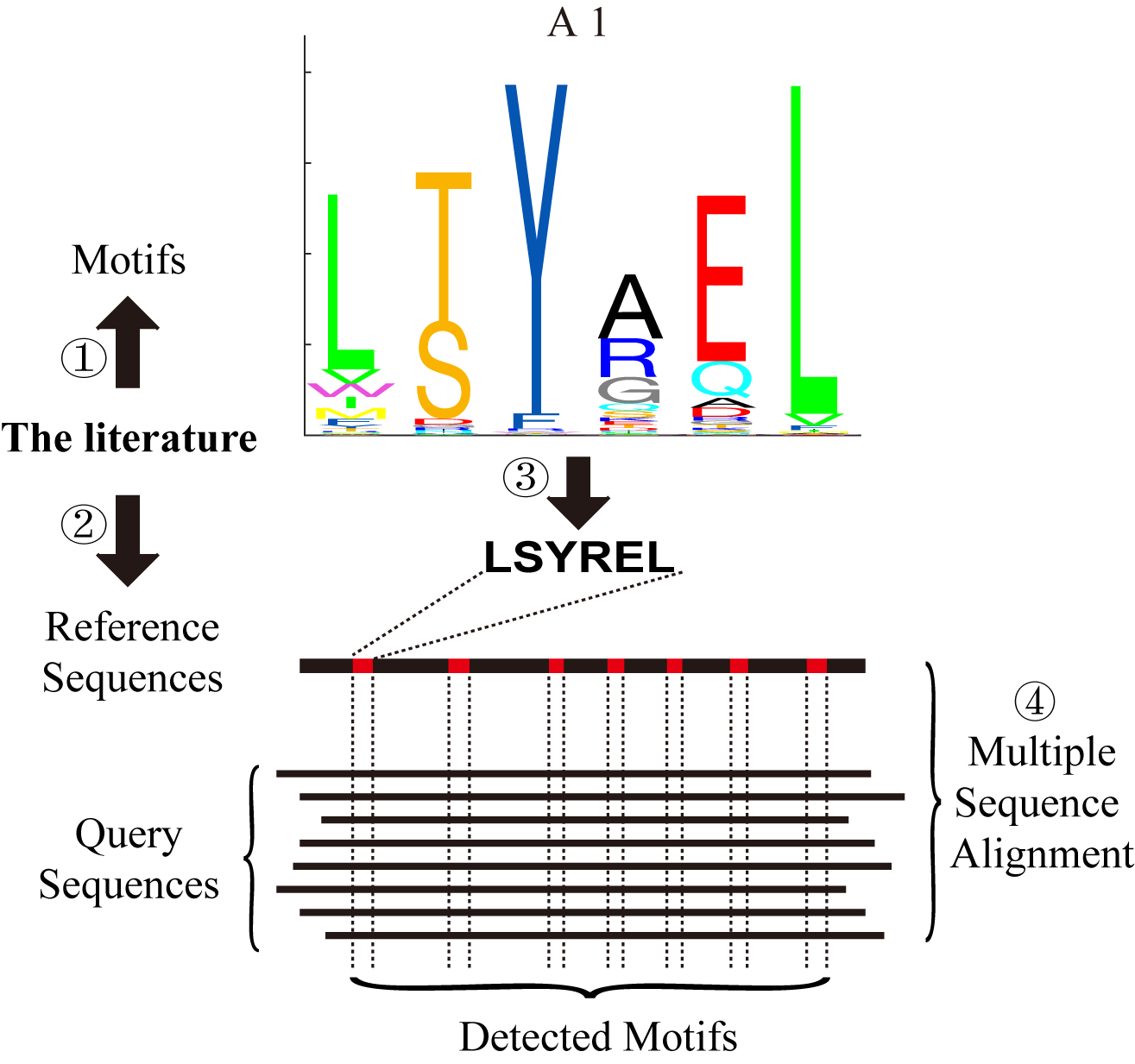

Supplement: S45 Fig — Illustration of the steps in locating known core motifs to query NRPS sequences. First, we curated known core motifs and reference sequences from the literature. Then known motifs on reference sequences were mapped according to previous research, with their locations recorded. Finally, multiple sequence alignment was performed between reference sequences and query sequences. Locations of core motifs in query sequences were inferred by the aligned reference sequences. (PNG) [file pcbi.1011100.s045.png]

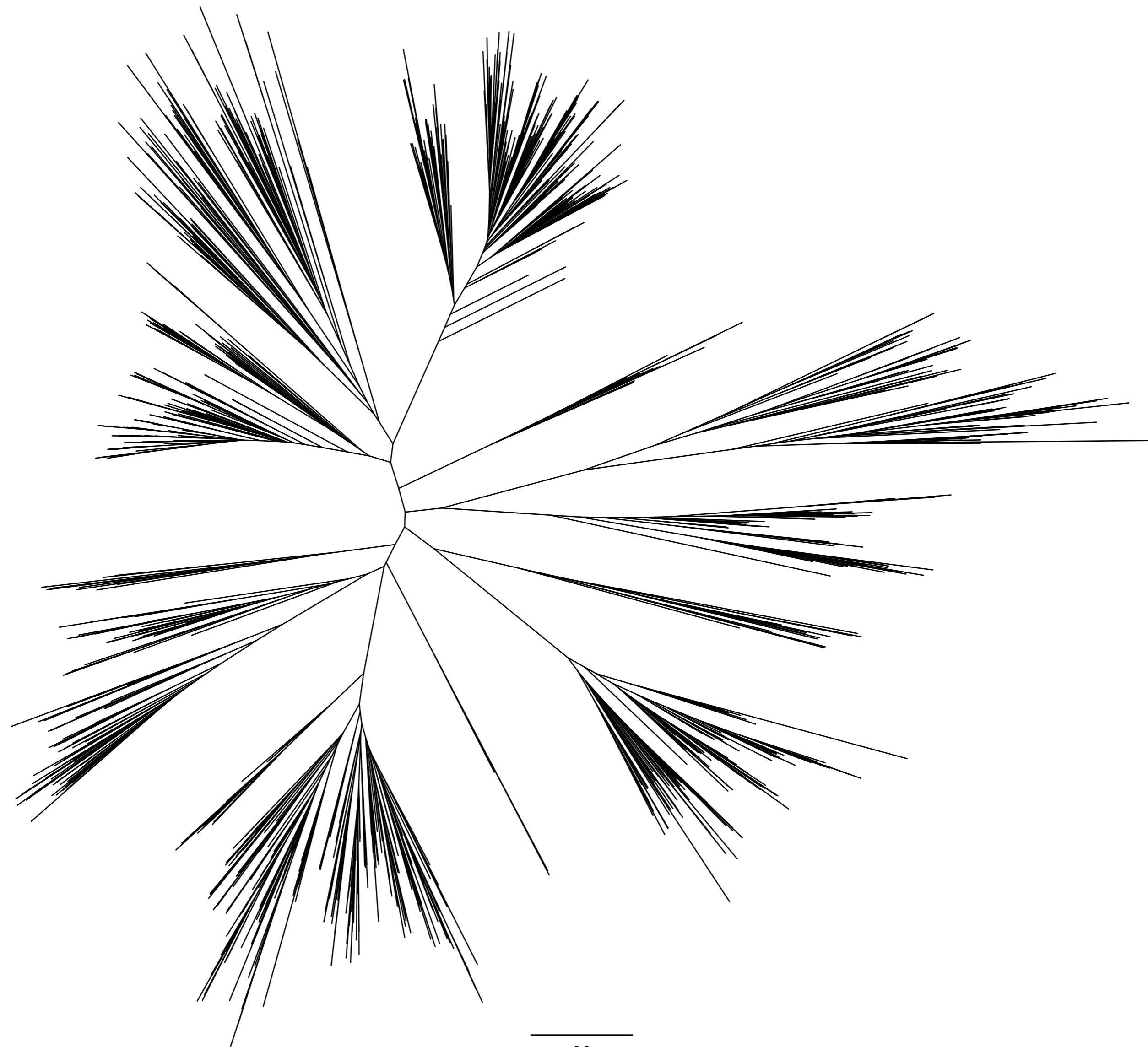

Supplement: S3 File — (ZIP) [file pcbi.1011100.s058.zip › Phylogenetic tree by IQ-TREE/C_all_trim_out6_MSA_trim.fasta.treefile.pdf]
