# Supplementary material for: Knowledge-guided data mining on the standardized architecture of NRPS: Subtypes, novel motifs, and sequence entanglements
Source: PLoS Comput Biol. 2023 May 15;19(5):e1011100. doi: 10.1371/journal.pcbi.1011100 (PMC10212144; doi:10.1371/journal.pcbi.1011100)
Supplement: S30 Fig — Similar to Fig 3A, we also analyzed 685 C+A+T+C (A) and 245 C+A+T+E (B) module NRPS sequences by SCA. Although sequence numbers are less than C+A+T composition NRPS (1,161), they also could provide insights for NRPS reengineering. Except known cutting points, the SCA result of C+A+T+E NRPS sequences indicated the junction between A and T domains may be a potential cutting point. Of note, cutting point proposed by Mootz et al.[1]. was for C+A+T+C in original research. (DOCX) [file pcbi.1011100.s030.docx]

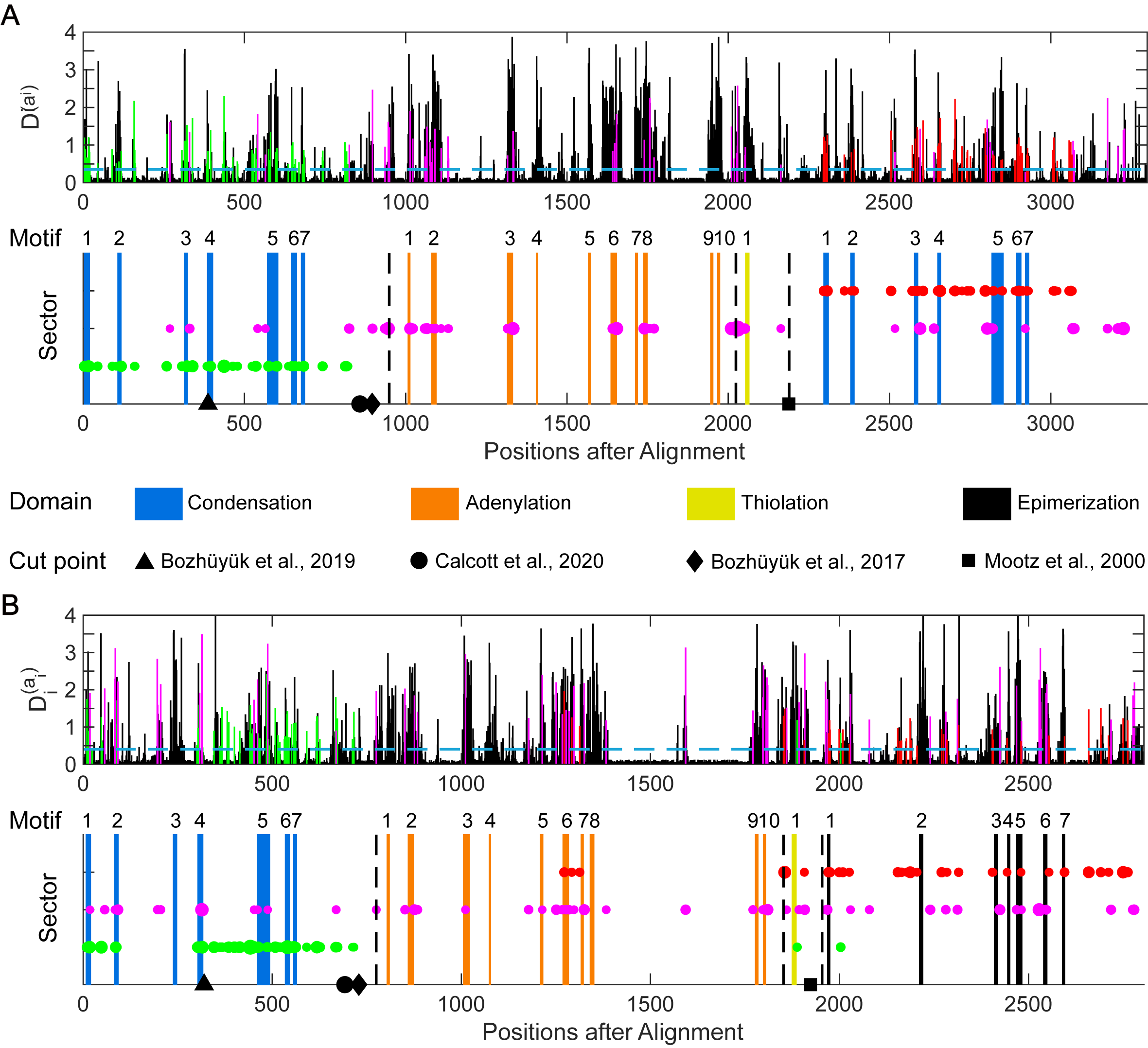


**S30 Fig. SCA analysis for C+A+T+C and C+A+T+E four domains NRPS sequences**

Similar to Figure 3A, we also analyzed 685 C+A+T+C (**A**) and 245 C+A+T+E (**B**) module NRPS sequences by SCA. Although sequence numbers are less than C+A+T composition NRPS (1,161), they also could provide insights for NRPS reengineering. Except known cutting points, the SCA result of C+A+T+E NRPS sequences indicated the junction between A and T domains may be a potential cutting point. Of note, cutting point proposed by Mootz et al.[1]. was for C+A+T+C in original research.

## Reference

1. Mootz HD, Schwarzer D, Marahiel MA. Construction of hybrid peptide synthetases by module and domain fusions. Proceedings of the National Academy of Sciences. 2000;97(11):5848-53. doi: doi:10.1073/pnas.100075897.
