# Supplementary material for: Knowledge-guided data mining on the standardized architecture of NRPS: Subtypes, novel motifs, and sequence entanglements
Source: PLoS Comput Biol. 2023 May 15;19(5):e1011100. doi: 10.1371/journal.pcbi.1011100 (PMC10212144; doi:10.1371/journal.pcbi.1011100)
Supplement: S1 Text — Table A, Table B, Table C. Table A. Product yields in different strains by HPLC/MS analysis. Table B. Fungal plasmids and strains used in this study. Table C. PCR primers used in this study. (DOCX) [file pcbi.1011100.s061.docx]

## Supplemental materials

## Knowledge-guided data mining on the standardized architecture of NRPS: subtypes, novel motifs, and sequence entanglements

Ruolin He^1^, Jinyu Zhang^2,3^, Yuanzhe Shao^4^, Shaohua Gu^1,4^, Chen Song^1,4^, Long Qian^1^, Wen-Bing Yin^2,3,*^, and Zhiyuan Li^1,4,*^

^1^ Center for Quantitative Biology, Academy for Advanced Interdisciplinary Studies, Peking University, Beijing, 100871, China

^2^ State Key Laboratory of Mycology, Institute of Microbiology, Chinese Academy of Sciences, Beijing 100101, PR China

^3^ Savaid Medical School, University of Chinese Academy of Sciences, Beijing, 100049, PR China

^4^ Peking-Tsinghua Center for Life Sciences, Academy for Advanced Interdisciplinary Studies, Peking University, Beijing, 100871, China

## The legend of Supplementary Materials

**S1 Text** contains **Table A, Table B**, **Table C**, and. See attached Excel files for **S1-S10 Table**, attached figures for **S1-S45 Fig**, and attached compressed files for **S1-S5 File**.

**Table:**

**Table A.** Product yields in different strains by HPLC/MS analysis

**Table B.** Fungal plasmids and strains used in this study

**Table C.** PCR primers used in this study

**S1 Table.** Source of C domain subtype reference sequences

**S2 Table.** Subtype prediction by NRPS Motif Finder and annotation by antiSMASH v5 for C domains in MiBiG

**S3 Table.** Subtype prediction by NRPS Motif Finder and annotation by antiSMASH v6 for C domains in 16,820 bacterial genomes

**S4 Table.** Subtype prediction by NRPS Motif Finder and annotation by antiSMASH v6 for C domains in 2,505 fungal genomes

**S5 Table.** Amino acid composition and conservation in three potential motifs

**S6 Table.** All available structures of AMP-binding-domain-containing proteins from the PDB database

**S7 Table.** Structural effects of point mutations in G-motif G409 of FmqC estimated by Missense3D

**S8 Table.** 2,636 A domains sequences information

**S9 Table.** Bacterial and fungus genome information used in this study

**S10 Table.** The definition of conserved motifs in NRPS domains and the positions in reference sequences.

**Figure:**

**S1 Fig.** Sequence logo of the seven C motifs among the multialignment of 1758 C domains (first row), and among six subtypes of C domains (second row) in MiBiG

**S2 Fig.** Sequence logo of the ten A motifs among the multialignment of 1859 A domains in MiBiG

**S3 Fig.** Sequence logo of T1 motif and the length distribution of the T1-C1 region in MiBiG

**S4 Fig.** Sequence logo of the seven E motifs among the multialignment of 310 E domains in MiBiG

**S5 Fig.** Sequence logo of the TE1 motifs among the multialignment of 280 TE domains in MiBiG

**S6 Fig.** Length distributions of C, A, and T domains, in MIBIG and in Pfam seeds

**S7 Fig.** Sequence logo of the twelve A motifs and two T motifs among 95,582 A domains and 86,688 T domains in bacteria

**S8 Fig.** Sequence logo of the seven E motifs among 14,502 E domains in bacteria

**S9 Fig.** Sequence logo of the TE1 motifs among 23,590 TE domains in bacteria

**S10 Fig.** Sequence logo of the twelve A motifs and two T motifs among 40,458 A domains and 26,651 T domains in fungi

**S11 Fig.** Sequence logo of the seven E motifs among 3,982 E domains in fungi

**S12 Fig.** Sequence logo of the TE1 motifs among 4,008 TE domains in fungi

**S13 Fig.** Comparison of NRPS A and T domain architecture between bacteria and fungi

**S14 Fig.** Comparison of NRPS E domain architecture between bacteria and fungi

**S15 Fig.** Sequence logo of the seven C motifs among the multialignment of 2,572 C domains (first row), and among 15 subtypes of C domains (second row) in MiBiG

**S16 Fig.** Sequence logo of the seven C motifs among 77,152 C domains (first row), and among 13 subtypes of C domains (second row) in bacteria

**S17 Fig.** Sequence logo of the seven C motifs among 34,269 C domains (first row), and among 11 subtypes of C domains (second row) in fungi

**S18 Fig.** Comparison of NRPS C domain architecture between bacteria and fungi

S19 Fig Sequence logo of highly conserved positions near known motifs

**S20 Fig.** The mutual information between residues in the A5-A6 and A domain substrate specificity

**S21 Fig.** The G-motif in different function states of LgrA structure

**S22 Fig.** The sequence logo of G-motif in A domains with known structures and FmqC

**S23 Fig.** The equivalents of N397, G409 (G-motif), and S491 in FmqC mapped to known structures

**S24 Fig.** LC-MS analysis in the strain construction

**S25 Fig.** Aα1 motif in different states of LgrA structure

**S26 Fig.** The interaction near Aα1 motif in different states of LgrA structure

**S27 Fig.** Conserved residues of T domain in the condensation state

**S28 Fig.** Eigenvalue spectra for the SCA matrix of C+A+T modules MSA and random MSA

**S29 Fig.** The interaction of C5-C6 with other domains in LgrA structure

**S30 Fig.** SCA analysis for C+A+T+C and C+A+T+E four domains NRPS sequences

**S31 Fig.** The gap frequency in the MSA of 2,636 A domains

**S32 Fig.** Clustering and groups of five loops

**S33 Fig.** The entropy and conditional entropy of the specificity-conferring code given different constraints.

**S34 Fig.** The sequence logo of the specificity-conferring code for substrate alanine in the dimension of phylum and loop group

**S35 Fig.** The sequence logo of the specificity-conferring code for substrate phenylalanine in the dimension of phylum and loop group

**S36 Fig.** The sequence logo of the specificity-conferring code for substrate leucine in the dimension of phylum and loop group

**S37 Fig.** The sequence logo of the specificity-conferring code for substrate valine in the dimension of phylum and loop group

**S38 Fig.** The sequence logo of the specificity-conferring code for substrate tyrosine in the dimension of phylum and loop group

**S39 Fig.** The sequence logo of the specificity-conferring code for substrate 2-amino-adipic-acid in the dimension of phylum and loop group

**S40 Fig.** The sequence logo of the specificity-conferring code for substrate glutamine in the dimension of phylum and loop group

**S41 Fig.** The sequence logo of the specificity-conferring code for substrate diaminobutyric acid in the dimension of phylum and loop group

**S42 Fig.** Loop length and group distributions in bacteria and fungi

**S43 Fig.** Causal analysis of A domain substrate specificity

**S44 Fig.** Protein sequence pairwise distance distribution

**S45 Fig.** Workflow of detecting conserved motifs in NRPS domain

**Other supplementary** **materials:**

**S1 File** C domain subtype reference HMM files

**S2 File.** C domain subtype reference sequences (MSA)

**S3 File.** The result file of the phylogenetic tree of C domain and E domain by IQ-TREE

**S4 File.** NRPS Motif Finder online version code (Python)

**S5** **File.** NRPS Motif Finder Matlab version code

### Table A

**Table A.** Product yields in different strains by HPLC/MS analysis

| Strains | Label | FQC relative concentration* | Compound 1 relative concentration* |
| --- | --- | --- | --- |
| TYJY81.1  TYJY81.2  TYJY81.3  TYJY82.1  TYJY82.2  TYJY82.3  TYJY83.1  TYJY83.2  TYJY83.3  TYJY84.1  TYJY84.2 | Δ*fmqC*  Δ*fmqC*  Δ*fmqC*  *fmqC* (control)  *fmqC* (control)  *fmqC* (control)  G409A  G409A  G409A  G409R  G409R | 0  0  0  199.258  230.414  270.880  225.824  221.679  203.156  198.312  245.373 | 39.826  39.861  40.401  0  0  0  0  0  0  0  0 |
| TYJY84.3  TYJY85.1  TYJY85.2 | G409R  G409D  G409D | 195.700  27.931  37.183 | 0  28.286  23.218 |
| TYJY85.3 | G409D | 31.764 | 23.974 |
| TYJY86.1  TYJY86.2 | G409P  G409P | 6.403  6.214 | 30.642  36.839 |
| TYJY86.3 | G409P | 8.460 | 41.718 |
| TYJY87.1 | G409W | 13.351 | 39.269 |
| TYJY87.2 | G409W | 13.653 | 40.303 |
| TYJY87.3 | G409W | 13.613 | 26.148 |
| TYJY88.1  TYJY88.2 | G409Y  G409Y | 18.441  13.979 | 32.551  25.974 |

*Concentration related to the compound in the 18 minutes in LC-MS result (Figure 4E).

### Table B

**Table B.** Fungal plasmids and strains used in this study

| Strains | Description | Reference |
| --- | --- | --- |
| Cea 17.1 | *Δku80* | [1] |
| Cea 17.2 | *Δku80*, *pyrG1* | [1] |
| TYJY81 | Δ*fmqC*⸬*hyg* | This study |
| TYJY82 | *fmqC*⸬*pyrG* | This study |
| TYJY83 | *FmqC409^gly->ala^*⸬*AfpyrG*, *pyrG1* | This study |
| TYJY84 | *FmqC409^gly->arg^*⸬*AfpyrG*, *pyrG1* | This study |
| TYJY85 | *FmqC409^gly->asp^*⸬*AfpyrG*, *pyrG1* | This study |
| TYJY86 | *FmqC409^gly->pro^*⸬*AfpyrG*, *pyrG1* | This study |
| TYJY87 | *FmqC409^gly->trp^*⸬*AfpyrG*, *pyrG1* | This study |
| TYJY88 | *FmqC409^gly->tyr^*⸬*AfpyrG*, *pyrG1* | This study |
| pYH-wA-pyrG | *URA3, WA flanking, AfpyrG, Amp* | [2] |
| pYJY25 | *FmqC*⸬*AfpyrG* in pYH-wA-pyrG | This study |
| pYJY26 | *FmqC409^gly->ala^*⸬*AfpyrG* in pYH-wA-pyrG | This study |
| pYJY27 | *FmqC409^gly->arg^*⸬*AfpyrG* in pYH-wA-pyrG | This study |
| pYJY28 | *FmqC409^gly->asp^*⸬*AfpyrG* in pYH-wA-pyrG | This study |
| pYJY29 | *FmqC409^gly->pro^*⸬*AfpyrG* in pYH-wA-pyrG | This study |
| pYJY30 | *FmqC409^gly->trp^*⸬*AfpyrG* in pYH-wA-pyrG | This study |
| pYJY31 | *FmqC409^gly->tyr^*⸬*AfpyrG* in pYH-wA-pyrG | This study |

TXX = original transformant, pXX = plasmid

### Table C

**Table C.** PCR primers used in this study.

| Primer | Oligonucleotide sequences (5’-3’) | Uses |
| --- | --- | --- |
| Hyg-FOR | cattccaatcgataccgtcgac | *G418* amplification |
| Hyg-REV | gtggataaccgtattaccgcc |  |
| fmqC-5F-F | cacttcaagtctgccaaagcg | *fmqC* 5′ flanks amplification |
| fmqC-5F-R | caatatcagttaacgtcgacggtatcgattggaatgtcgatcaagtgtcgttgtctccg |  |
| fmqC-3F-F | atcagctcactcaaaggcggtaatacggttatccacgaagggacctgagataggtttgt | *fmqC* 3′ flanks amplification |
| fmqC-3F-R | aacctggcaatcatgacgat |  |
| fmqC-NEST-F | tcagcacacccttaccgaag | *fmqC* deletion cassette amplification |
| fmqC-NEST-R | acaaatccttgcgctgttct |  |
| fmqC-RT-F | gttctgcacctgtgcactc | *fmqC* transformant screening |
| fmqC-RT-R | ggtgcatcggcttcatcttc |  |
| pyrG-F | gagagttattctgtgtctgacgaaa | *pyrG* amplification |
| pyrG-R | attctgtctgagaggaggcac |  |
| fmqC-WA-F | ctccttctcctgatcgataggtatcctgcatgtgtgcg | construct mutant plasmids |
| fmqC-pyrG-R | cacagaataactctcagtgcgatatatttgtgctgct |  |
| pyrG-3F-F | cctctcagacagaataattccgtttctttcctgcgg | construct mutant plasmids |
| 3F-WA-R | gtgattcgcgtcatgcggccgccaatcaacactgctctaccgac |  |
| fmqC-G to A-F | gatcgcattccccgtcagcgtggtaccctgggtagtgg | FmqC409^gly->ala^ |
| fmqC-G to A-R | cgctgacggggaatgcgatcttgctcggatgactc |  |
| fmqC-G to R-F | gatcagattccccgtcagcgtggtaccctgggtagtgg | FmqC409^gly->arg^ |
| fmqC-G to R-R | cgctgacggggaatctgatcttgctcggatgactc |  |
| fmqC-G to D-F | gatcgacttccccgtcagcgtggtaccctgggtagtgg | FmqC409^gly->asp^ |
| fmqC-G to D-R | cgctgacggggaagtcgatcttgctcggatgactc |  |
| fmqC-G to P-F | gatcccattccccgtcagcgtggtaccctgggtagtgg | FmqC409^gly->pro^ |
| fmqC-G to P-R | cgctgacggggaatgggatcttgctcggatgactc |  |
| fmqC-G to W-F | gatctggttccccgtcagcgtggtaccctgggtagtgg | FmqC409^gly->trp^ |
| fmqC-G to W-R | cgctgacggggaaccagatcttgctcggatgactc |  |
| fmqC-G to Y-F | gatctacttccccgtcagcgtggtaccctgggtagtgg | FmqC409^gly->tyr^ |
| fmqC-G to Y-R | cgctgacggggaagtagatcttgctcggatgactc |  |

## Reference

1. d'Enfert C. Selection of multiple disruption events in Aspergillus fumigatus using the orotidine-5′-decarboxylase gene, pyrG, as a unique transformation marker. Current Genetics. 1996;30(1):76-82. doi: 10.1007/s002940050103.

2. Yin W-B, Chooi YH, Smith AR, Cacho RA, Hu Y, White TC, et al. Discovery of Cryptic Polyketide Metabolites from Dermatophytes Using Heterologous Expression in Aspergillus nidulans. ACS Synthetic Biology. 2013;2(11):629-34. doi: 10.1021/sb400048b.
