# Supplementary material for: Knowledge-guided data mining on the standardized architecture of NRPS: Subtypes, novel motifs, and sequence entanglements
Source: PLoS Comput Biol. 2023 May 15;19(5):e1011100. doi: 10.1371/journal.pcbi.1011100 (PMC10212144; doi:10.1371/journal.pcbi.1011100)
Supplement: Attachment — Submitted filename: review-ploscomp.docx [file pcbi.1011100.s062.docx]

The authors here present work focusing on NPRSs, a well-studied family of enzymes useful in particular for protein engineering and reengineering largely due to its modular structure. With the rapid expansion of sequencing techniques and subsequent sequencing data, the amount of available information on NPRSs has increased dramatically; however, with the increase in sequencing data has come an increase in annotation standards and variety making meta-level analysis leveraging widely-used techniques such as MSA difficult to implement within this enzymatic family. The authors provide their take on a standard architecture in which conserved core motifs within various domains are used as lynchpins for constructing a “coordinate system” for NRPSs. The authors then utilize this novel system for statistical and functional analyses including the identification of specific, conserved interdomain positions, interesting analysis motif and intermotif MI with respect to subdomain type, SCA and correlation analysis for residues within and between domains and motifs and the potential ability to distinguish between bacterial and fungal NPRSs (and more). Overall, the authors provide compelling evidence for the usage of this newly defined NRPSs categorization and this work should provide a great fit  for PLOS Computational Biology after addressing the concerns discussed below.

(1) One major concern is the missing link or the consensus among different analysis: It would be great if authors try to find general link between different analysis that provides solid design.

(2) The figure 1B ( MI analysis) very interesting, with high degree of MI for A domain subtypes in 10-S A4-A5 region as expected. As a suggestion, I would appreciate any additional commentary on theC5-C6 inter motif region where the MI for C domain subtypes is nearly as high on average as C6 and higher than C7. Interestingly, residues of this region again appear in Figure 4 as containing some green and red (C-motif and A-motif spanning sectors).

(3) In section 2.2 the authors identify a conserved motif between the C and A domains and falls near the low MI region of the far-right orange in Figure 1A bottom panel. This motif then has low MI with C domain subtypes, but also forms bonds with A4. Were any of these positions found to be within the magenta or red sectors from SCA? If not, can the authors provide a comment why one might find conserved positions consistently forming H-bonds with A motif absent within the A-spanning or A-heavy sectors?

(4) Figure 4C; I would be interested in additional discussion with this. Particularly, it seems that there are several residues in the green sector that correlate strongly with the red sector – by inspection, it appears that some of these “span the gap” to correlate with the red sector positions for which magenta correlations are noticeably absent.

(5) In the second paragraph of section 4.2 the authors state they compare loop amino acid (AA) length with Euclidean distance between A domains and then state that loop-based distance is connected with similarity of sequence alignments. The only way this statement makes sense is if the authors are suggesting AA length is directly correlated with sequence alignment; if this is the case, the authors should state this. Also, the authors state this loop-based distance is connected with the domain A phylogeny (Fig 5B); this does not appear to be the case at all based upon what can be discerned from Figure 5B outside of the Firmicutes-Loop group 1 relationship.

(6) The statement at the end of section 5 (line 590) “In summary, bacterial and fungal NRPS show significant difference in the length of intermotif. It makes it possible to distinguish between bacterial and fungal NRPS in our standardized architecture.” seems too strong. While the authors provide evidence that there are some differences in intermotif length between bacteria and fungi, it does not appear that this will be sufficient to distinguish a given NPRS as belonging to either bacteria or fungi.

As also other minor points

(7) Figure 5 – I found the color-coded keys for this figure difficult to interpret as keys for substrate type are used in B and C are placed adjacent to A, the loop-group key is only used in B and is placed next to A and the phylum group, used in all A B and C is placed next to C but is necessary to correctly read A.

(8) The sentence “The sector containing positions densely covering the A domain pocket region appears till the sixth biggest mode (Fig 4A, red dots)” lines 441-443 is unclear – perhaps a typo in the usage of the word “till”?

(9) Line 333 sentence “conserved suggests causes for the conservation is beyond forming hydrogen.” should end in “bonds.”

(10) Violin plots in the second panel in Figure 1 is very small, hard to understand needs revision.

(11) Figure 3 B it is stated in caption “The mutated tryptophan380 was marked in purple” . It should be not purple but magenta
