# Supplementary material for: Knowledge-guided data mining on the standardized architecture of NRPS: Subtypes, novel motifs, and sequence entanglements
Source: PLoS Comput Biol. 2023 May 15;19(5):e1011100. doi: 10.1371/journal.pcbi.1011100 (PMC10212144; doi:10.1371/journal.pcbi.1011100)
Supplement: Attachment — Submitted filename: RESPONSE_HRL20230122_02_08.docx [file pcbi.1011100.s063.docx]

Dear Editors and Reviewers:

We are pleased to submit our revised manuscript entitled "Knowledge-guided data mining on the standardized architecture of NRPS: subtypes, novel motifs, and sequence entanglements." The previous title was "Non-ribosomal peptide synthetase domain boundary identification and new motifs discovery based on motif-intermotifs standardized architecture." The revision includes two Word files for the clean and uncleaned main text revisions with the prefix of “main_draft_standardNRPS_HRL_2023_02_08”, a Word file for SI information “SI_draft_standardNRPS_HRL_2023_0207” containing Figure S1-S45, Table S8 and Table S12-S13, ten Excel files including Table S1-Table S7 and Table S9-S11, five compressed files including codes, sequences, phylogenetic tree and HMM files. The content of five compressed files is described in the SI Word file.

We would like to express our gratitude for the valuable feedback and suggestions provided by the editors and reviewers, which have greatly assisted us in enhancing the manuscript and uncovering new insights.

The detailed responses to each reviewer are listed here. Our responses are in blue text:

## Reviewer #1:

Present manuscript from Zhang et al. deals with a very important question in the field of NRPS engineering, namely the identification of domain boundaries and, in addition, discovered new un-described motifs, i.e. within the NRPS adenylation domains (G-motif). The manuscript provides a very comprehensive and detailed overview of the motif and inter-motif architecture of NRPSs and gives a long overdue update on conserved motifs within NRPSs. Additionally, all results are embedded and discussed in the current state-of-the-art and the most important literature.

Although I personally do like this work a lot, because it gives a lot of insights on how to (potentially) improve future engineering campaigns, I also could imagine that this publication is very difficult to comprehend for non-experts in the field. Nevertheless, the NRPS engineering community, including me, will highly appreciate and value this work.

We are grateful for the reviewer's insightful comments and appreciate the opportunity to clarify our approach. We have revised the manuscript to better emphasize the background and scientific challenges in order to make the content accessible to a wider audience, including non-specialists in the field.

Some minor comments:

The first group using A subdomain swaps for NRPS engineering was the Piel group and not Kries et al.

And the Micklefield lab only was able to efficiently introduce (swap) subdomains from highly related origins with very similar and identical specificities.If I recall this correctly the greatest introduced chemical change was Ser/Thr. Even subdomains with the same specificities from different origins yielded low titers.

We thank the reviewer for this comment, and have made revisions accordingly. Subdomains defined by the Piel group and Kries et al. almost overlap in their positions (Additional Figure 1. In the Discussion section, we mentioned the subdomain swap strategy from Kries et al. first, because their cutting point exactly was G-motif. Moreover, they stated in their work that their cutting point was selected from structure insight (flavodoxin-like subdomain), while we proposed this cutting point from sequence analysis. Consistency between these two distinctive methodologies was exciting for us, so we emphasized the results from the Kries group. Nevertheless, as the reviewer stated, the first A subdomain swap work indeed came from Piel group in 2013 (<https://doi.org/10.1039/C2SC21722H>). In the revised version, we included the work of the Piel group in the Discussion section.

**Additional Figure 1. The comparison between subdomain swap strategies from the Piel group and Kries et al.**

Figure modified from Review about NRPS re-engineering (PMID: 26699732)

In the original paper of Kries et al., substrate specificity was successfully changed from Phe to Val, two amino acids having considerably different chemical properties. So, we suspect that the strategy of using the G-motif as the cutting point has the potential to significantly change the chemical properties of the substrate. And in Micklefield’s work, as stated by the reviewer, they indeed didn’t introduce too much chemical change, as their main purpose was to prove that the CRISPR-Cas9 gene editing system is applicable to the rapid engineering of NRPS. We mentioned Micklefield’s work as it is relatively recent (2021 November) and also used the same cutting point as Kries et al., i.e., the G-motif.

However, the current manuscript contains many grammatical errors, especially the confusion of singular and plural, missing words and some typos. Even Therefore, the manuscript would benefit from correction by a native speaker.

We are grateful to the reviewer for bringing this to our attention. We have taken the opportunity to carefully review the grammar and language of the manuscript and have sought professional assistance to improve it.

## Reviewer #2:

This manuscript represents an attempt to address an important problem in the biosynthesis of non-ribosomally produced peptide natural products. The models that we have to identify and define proteins and domains often do not reflect recent structural and biochemical data, and lack of clarity regarding domain boundaries and regarding the conserved regions involved in important domain-domain interactions make NRPS engineering efforts challenging. Additionally, the complex relationship between the well-established Stachelhaus specificity codes and substrates is frustrating: why do multiple codes result in the binding of one substrate? Why are some codes less specific? In this manuscript, the authors examine NRPS modules (particularly the C and A domains) in depth, attempting to tie specific sequence motifs and loop lengths to altered substrate binding behavior. They also use a set of conserved motifs (as well as sector-based conservation analyses) to better understand domain boundaries. However, based on the data presented, I am not sure the authors have been able to provide enough support for the relevance and broader applicability of the trends that they observe.

We appreciate the reviewer's thoughtful comments. We have taken great care to address all of the questions, suggestions, and comments, and have made substantial revisions to enhance the relevance and practicality of our work.

Major points of concern:

1. The proposal that a conserved A-domain glycine provides vital flexibility is plausible, and certainly replacement by bulkier amino acids might decrease that flexibility, as suggested in line 343. But the introduction of a bulky, charged residue (G409R) apparently has negligible effect on the production of fumiquinazoline C in the test system; it is not clear how this is reconciled with the authors' model, since the presence of a glycine or even a small uncharged amino acid is apparently not actually "critical" (line 368)? (Given the error bars in Fig. 3F, I'm not sure I'd even confidently say that glycine replacement "always" decreases yield as described on line 654.) Similarly, only two small amino acids were tried. One appeared to have no effect on activity (G409A) and the other (G409P) replaced the most conformationally flexible amino acid (Gly) with the least conformationally flexible amino acid (Pro). While proline replacement did hurt activity, it's not at all clear that this would be true for other smaller amino acids that don't introduce a tertiary backbone amine (Thr, Ser, Cys, Val). The conservation of N397 is also not discussed; if it is not highly conserved, is the proposed steric clash a problem that really can be expected to apply across NRPS domains? Additionally, the authors use a Phyre2 model of FmqC. Even AlphaFold2 urges caution regarding to sidechain conformation, and I haven't seen evidence that Phyre2 outperforms AlphaFold2 in that regard unless there is a close homolog available for modeling (if there is, it is not discussed). What do the equivalent Gly409 and N397 positions in known crystal structures of A domains look like? Several are even referenced in this paper, and quite a few more are available: https://www.acsu.buffalo.edu/~amgulick/NRPSChart.html (In this context, it's also odd that several A-domain subdomains are listed in the SI - fig. S9 and S11 - but never discussed in the context of how the identified motifs in this manuscript might connect to their boundaries.)

We thank the reviewer for these comments.

First, regarding the question of why we did not try other smaller amino acids that don't introduce a tertiary backbone amine, we selected them to cover combinations of structural effects (Table S7 in the revision), as determined by the Missense3D tool developed by Professor Michael Sternberg's group (http://missense3d.bc.ic.ac.uk/missense3d/). The structural effects we considered included replacement of buried glycine, introduction of clashes, changes in exposure, introduction of buried charges, introduction of buried proline, and violations of disallowed phi/psi angles (PMID: 30995449). Our previous lack of explanation for the mutation experiment design in the initial version of the paper has been addressed in this revision, at line 617-618.

In addition, to rule out the possibility of errors in fumiquinazoline pathway experiments, we repeated the mass spectral measurement three times for each mutant (Additional Figure 2). The result is highly similar to prior measurements: There is no significant difference between WT and G409A or G409R, whereas all other mutants significantly reduced FQC production.

**Additional Figure 2. The repeated point mutation experiments**

Given that the experiment is reasonably valid, we also pondered why mutations such as G409A and G409R appear to have little effect. To gain further insights, we conducted additional computational analysis. Our updated dataset, which was expanded based on the reviewer's suggestion, confirmed the high level of conservation of glycine in the G-motif across 95,582 A domains in bacteria (Figure S7 in the revision) and 40,458 A domains in fungi (Figure S10 in the revision). Typically, a high degree of conservation indicates strong selection pressure. For further confirmation, we checked the exceptions: 13 A domains in the MiBiG database that do not contain the conserved glycine in G-motif. Our findings showed that these exceptions occur in either single-A domain pathways or the last module in a pathway. This observation suggests the selection pressure is relatively reduced when A domain is the only or the last one in the pathway, which might be attributed to that they don’t need to pass the substrate to the next module and therefore don’t need to go through large conformational changes.

Therefore, we suspected that our prior observation that G409A and G409R in *fmqC* have a negligible effect may be attributed to the fact that they are in a single-A domain pathway. To validate this, we performed point mutation experiments (replacing Gly with Ser, Val, Ala, Arg, and Asp) in the three-module pathway of fmqA (Figure 4C in the revision). The results showed that mutations of the Gly residue had the biggest impact on product yield when they occurred in the first module of multi-module NRPS. On average, the effects of mutations in the last module were the least, consistent with the computational analysis of exceptions in MiBiG. The introduction of a bulky and charged residue (G to R) significantly reduced the yield of the product, which makes sense given the high conservation of glycine with the smallest side chain among proteinogenic amino acids. This result suggests that the importance of glycine in the G-motif is position-dependent in the A domain, possibly related to the dynamic process of NRPS producing the product, such as shuttling the substrate between different modules. While it has been reported that NRPS undergoes significant conformational changes during biosynthesis and handoff between modules, as demonstrated by full dimodule crystal structures of LgrA, the dynamics of NRPS proteins with more than two modules are still unknown. Further studies of the G-motif may shed light on this mechanism. Limited to our time, the 15 point mutation experiments only finished once without biological replicates (Additional Figure 3). More information about the G-motif mutations was not included in this article due to its length, and we plan to delve deeper into the mechanism of the G-motif in future projects. So far, the results have been able to support the importance of flexible glycine in G-motif for NRPS product yield.

**Additional Figure 3. Mutations of the G-motif in a 3-module** **pathway fmqA supports the function of the conserved domain in the biosynthesis of fumiquinazoline F**

**A.** The *fmq* gene cluster responsible for the production of FQF. Ant: non-proteinogenic amino acid anthranilate. C* represents a truncated and presumably inactive C domain. C_T_ represents a terminal condensation-like domain that catalyzes the macrocyclization reaction.

**B.** The biosynthetic pathway for FQF.

**C.** LC-MS analysis of the control: No *fmqA* (first row), *fmqA* (WT, second row) and the 15 point mutation strains.

**D.** Yield of FQF in different strain types.

**E.** Normalized yield of FQF in different mutation strains by FQF yield in WT.

Regarding the question of whether close homologs are available for modeling, Phyre2 identified high-score PDB templates as 5U89, 6MFZ, and 6N8E for predicting the FmqC structure, with alignment coverages of 93%, 94%, 87%, and identities of 25%, 23%, 21%, respectively. According to the reviewer’s suggestion, we added the discussion on homologs used for modeling in the Method Section.

Regarding the question of which tool is more appropriate for predicting the FmqC structure, Phyre2 or AlphaFold, we compared the results of both algorithms and found that they are highly similar. At the time we obtained the prediction result from Phyre2 (in December 2021), the AlphaFold Protein Structure Database paper had just been published (in November 2021) and the ColabFold web server using AlphaFold2 had not yet been launched (published in May 2022, PMID: 35637307). At that time, we lacked confidence in the reliability of the AlphaFold database and instead used the more conventional Phyre2 method as recommended by Missense. However, since then, the AlphaFold Protein Structure Database has been widely validated and the predicted structure of FmqC can now be accessed on the database (<https://alphafold.ebi.ac.uk/entry/Q4WLW8>). The predicted structure by SWISS-MODEL can also be accessed online. (<https://swissmodel.expasy.org/repository/uniprot/Q4WLW8?csm=FAA8BE224F567D84>), which used PDB ID 6MFZ for homology modeling. We carefully compared the predicted structures by Phyre2, AlphaFold2, and SWISS-MODEL (Additional Figure 4). They are essentially identical in the A core domain (motif A1-A8) where the G-motif is located. With the exception that the side chain orientation of F409 predicted by AlphaFold2 differs slightly from the other two predictions, the structures of the G-motif and N397 are consistent. Taken together, we believe that the algorithm choice does not significantly affect our predictions.

**Additional Figure 4. Comparison of FmqC structures predicted by Phyre2, AlphaFold2 and SWISS-MODEL**

Regarding the conservation of N397, we included related discussions in the main text. N397 was F493 in the LgrA protein, as described on lines 600 and 947 in the main text. And its degree of conservation was summarized in Table S5 in this revision (cells D7 and E7 of Sheet 1). In our MiBiG CAT module MSA, position 1929 corresponds to N397 in FmqC (Table S5 in this revision). Its degree of conservation is indeed moderate, with an AACon score of 6 (in the range of 0–10) and a ConSurf score of 6b (in the range of 1-9, "b" represents buried residue). Nevertheless, the majority of amino acids appearing in this position have bulky side chains, such as Trp/Tyr/Phe. The average size of amino acids in this position is 5.1 (details refer to the new Sheet2 of Table S5 in this revision). Considering the size of Asn (N) is 5 (<https://www.genome.jp/entry/aaindex:DAWD720101> ), we think Asn can roughly represent the amino acids at position 397 in terms of size.

Regarding the reviewer’s suggestion for checking the equivalent Gly409 and N397 positions in known structures of A domains, we downloaded and analyzed all 39 AMP-binding-domain-containing proteins from the PDB database (95 structures with different conformations or ligands in PDB database, Table S6 in the revision). Of these 39 proteins, 30 are from NRPS pathways (selecting 20 different substrates), 6 are from NRPS-like pathways (selecting 5 different substrates, with carboxylate reductase is the carboxylate, regarded as one kind of substrate here), and 3 are from D-Ala-ligases (DltA) pathways (selecting substrate D-Ala). Of these 30 A domain proteins from NRPS pathways, 13 contain a single A domain, and 19 contain multiple domains. And StsA has both two types. Another overlap case is EntE (single A domain) and fusion protein EntE-EntB (A+T domain). The structures of LgrA protein used in our study (Figure 3B-3D and Figure S21 in the revision) are the only structures containing two continuous modules (F+A+T+C+A+T). Therefore, we think that LgrA structures would provide us with the most accurate insight about the dynamics of NRPS when multi-module interactions are involved.

We appreciate the reviewer's suggestion to verify the homology of the FmqC protein structure. To confirm the sequence homology between the FmqC protein and other PDB A domains, we created a multiple sequence alignment (MSA) and a phylogenetic tree based on the A core domain sequence (which covers A1-A8, as shown in Additional Figure 5). The results indicated that the most similar A domain to FmqC was SidN (PDB ID: 3ITE), which is reasonable since SidN is the only fungal NRPS found in the PDB protein database. It's worth noting that the sequences were clustered based on both substrate and phylogeny (as shown in Additional Figure 5 and Table S6 in the revised manuscript, "tree" sheet), consistent with the results in Section 4.

**Additional Figure 5. Phylogenetic tree of the A core domain sequence (A1-A8) from proteins with known structures and FmqC.**

The same substrate that occurred more than once was marked with its designated color. These singular occurrences were documented in Table S6 in the revision. The query sequence FmqC was highlighted by the red box.

Out of the 39 proteins analyzed, the NRPS-like carboxylate reductases (CARs) do not possess the G-motif, indicating a unique evolutionary pathway despite having an AMP-binding domain (see Table S6 in the revision, "overview" sheet). On the other hand, the G-motif is present in all 30 NRPS A domains and 3 NRPS-like A domains, excluding CARs (refer to Figure S22 in the revision).

Regarding the question about the equivalent Gly409 and N397 positions in known crystal structures of A domains, we first noticed that in the LgrA structure, in addition to N397, another site, S491, may play a similar role as N397 (Figure S23 in the revision). That is, the large amino acid in position 409 frequently collides with amino acids in N397 and S491. Amino acid composition at the 491 position was shown in the new Sheet3 of Table S5 in the revision (N577 in LgrA, see Figure 3B-3D and Figure S21 in the revision; N2090 in MiBiG CAT module MSA, see Table S5 in the revision). The most prevalent amino acids in this position are Asn/Thr/Glu, which all have moderate size of side chain. Then, we carefully checked every equivalent G409 and N397 positions in known structures of A domains from PDB, and similar phenomena were observed (Table S6 in the revision).All proteins have equivalent N397 and S491 except the NRPS-like protein poly-e-Lys synthetase (Pls-A, PDB: 7WEW), which lacks an equivalent S491. Even for CAR proteins, they don’t have the G-motif but still retain the equivalent N397 and S491 as gatekeepers (Figure S23 in the revision, PDB: 6OZ1). We also noted that some equivalent N397 and S491 in known structures are not perfectly aligned with N397 and S491 of LgrA in multiple sequence alignment (1~2 AA offset, Table S6 in the revision). These are A domains in single-domain organizations or with large (aromatic) substrates such as Phe/Tyr/DHB/SA. Because of the small protein size, the requirement for flexibility in A domains in single domain structures may be reduced. For large (aromatic) substrates, it may need more space in the A domain pocket, which stretches structures near the G-motif. In summary, among these known structures, Gly in the G-motif (G409) is always adjacent to the side chains of two residues (N397 and S491) in all Non-CAR A domain structure, which may explain why this position has a strong preference of Gly. Another insight from these known structures is that the G-motif is in close proximity to the adenylate part of the ligand (Figure S23 in the revision), suggesting a potential gatekeeper role. We added related discussions in line 590-595.

In response to the reviewer’s question about why Figure S9 (Figure S21 in the revision) and S11 (Figure S25 in the revision) were not discussed in the context of possible connections between the identified motifs and their boundaries, Figure S9 (the predicted structure) is actually a supplement to Figure 2B-2D (Figure 3B-3D in the revision, the simplified cartoon). The visualization from the predicted structure is not very intuitive, so we utilized a simplified cartoon in the main text (Figure 2B-D). Figure S11 (Figure S25 in the revision) was discussed in Section 2.2. Regarding the boundary identification, we didn’t discuss too much about the “Aα1” motif (Figure S25-S26 in the revision) as well as the “Tα1” motif (Figure S27 in the revision) due to the length limitation of the article. We think the Aα1 and Tα1 motifs can be defined as the “boundary” of the full A domain, because these two motifs are located at the beginning of the A and of T domains respectively. In the prior version, the “boundary” discussed in this manuscript focuses on re-engineering. We had revised the text to state the definition of “boundary” more clearly in Section 3.

2. The domain architecture of FmqC appears to be ATC, while all the analysis in sections 2.1 and 3 focuses explicitly on systems with CAT(-/C/E) architecture. Are differences in domain architecture expected to affect the applicability of any of the results? If not, does limiting the initial analysis to CAT(-/C/E) module sets and not other domain structures artificially affect the analyses by providing a more limited dataset? Similarly, new domains (Cmod, or the interface domain involved in substrate beta-hydroxylation) and additional tailoring domains (methyltransferase, hydroxylase) are not discussed. Since many NRPS systems are more complicated, it would be helpful for the authors to state more clearly whether or not they expect these results to be more broadly applicable, and whether or not similar analyses will need to be made independently for many domain architectures.

We thank the reviewer for these questions and suggestions. Regarding the question about that the domain architecture of FmqC is ATC while analysis is for CAT(-/C/E) architecture, in Figure 3C of the prior version, the domain architecture of FmqC was shown as ATC for simplicity. By the updated NRPS Motif Finder, we confirmed the subtype of this C domain is C_T_ domain, a type of common termination domain in fungal NRPS (PMID: 22902615, PMID: 33941694). We revised that figure to make it more rigorous. We think using the FmqC pathway to explore the function of the conserved Gly in the G-motif is valid, because it also has the conserved Gly in the G-motif of its A domain. Moreover, the conservation of the G motif was evaluated by analyzing sequences of A domains instead of a whole module (Figure 1A). Therefore, we believe results related to the G motif are relatively universal to both C+A+T and A+T+C organizations. Details of the G motif were presented in detail in Figure 3 in the revision, together with the Aα1 and Tα1, aiming to show the relative position of the three motifs.

In addition, despite that we didn’t analyze A+T+C specifically, we can get related insights from the C+A+T+C results in Section 3 (Figure S30 in the revision). The first C domain of C+A+T+C is relatively independent from the other domains, as shown in the green sector in Figure S30 in the revision.

As mentioned by the reviewer, C domains have multiple subtypes, most related to substrate chirality. In generating Figure 1,3 and 5-6 in our revised manuscript, all C domain subtypes were aligned together in order to acquire an overall landscape of NRPS architecture. The statistics of C domain subtypes in the dataset we used were summarized in Figure S1 in the revision. The dataset included Dual, LCL, DCL, Starter, and Heterocyclization subtypes that commonly appeared in the original records in the MiBiG v2 database. Among them, LCL subtype is the most frequent, followed by Dual E/C and DCL subtypes. These three subtypes account for 86.12% of the C domains we used. The number of other C domain subtype sequences is relatively small. In addition to these common subtypes, in this database our updated NRPS Motif Finder identified 19 subtypes of C domains. We further confirm predictions in rare subtypes such as modAA, I, bL, PS by literature. Therefore, our result is universe, especially for common C domain subtypes in bacteria.

However, the number of other additional tailoring domains is very small. There were only 118 methyltransferase domain sequences and 25 hydroxylase domain sequences in this database. If the number of sequences is too small, no meaningful conclusions can be drawn from sequence analysis. These additional tailoring domains are indeed important, yet their computational analysis was restricted by limited sequence numbers. In following investigations, we plan to analyze these domains and architectures once there are enough sequences to reach statistical significance.

3. The cysteine example in Fig. 5C is intriguing, but it seems like it would be quite useful to have equivalent information for all Stachelhaus codes & substrates. It's the kind of information that might help other researchers better understand the relationships between their NRPS A domains of interest and the reasons that they may deviate from established Stachelhaus codes. Unfortunately, I don't think this data is presented anywhere, even in the SI files?

We thank the reviewer for this suggestion. As the classical references, Stachelhaus codes (the specificity-conferring code) with substrates are indeed very important information for researchers in this field. Related information details have been listed in Table S3 (Table S9 in the revision) about 2363 A domains sequences information such as Stachelhaus codes, substrates, loop length, loop group, species, MiBiG id or UniProt id, and related sequences. In our previous version, Table S3 (Table S9 in the revision) was only mentioned in the Method Section. We appreciate the insights from reviewers, and added more description in the revised main text about Table S3 (Table S9 in the revision) at line 794-795. And headers of Table S3 (Table S9 in the revision) were modified for better readability.

In addition, we added sequence logos of Stachelhaus codes for 8 substrates (4 most abundant substrates in our database and 4 representative substrates) in accordance with phylum, loop group, or both (Figure S34-41 in the revision). We hope this visualization is more intuitive for readers than Table S3 (Table S9 in the revision). Figure S34-41 are related to Figure S19 (Figure S33 in the revision), which indicates information from the phylum and the loop group both reduce the uncertainty of Stachelhaus codes, and they together could further reduce the uncertainty. Also included in the supplementary materials are the original files for these eight supplementary figures and other substrates.

4. While interesting, it is unclear what the differences in average "intermotif" length actually mean in terms of changes to substrate identification in fungal strains. This section isn't really connected to the previous sections, and would really benefit from some discussion about how and why we might expect some of these differences to specifically affect our ability to predict substrates. Even in datasets where the AAD-associated sequences are removed, median and standard deviation seem more informative than the mean: the differences in bacterial and fungal intermotif length in A1-A2 and A7-A8 may be statistically significant in a narrow p<0.05 sense, but it's unclear that they're of any use when considering the architecture of individual proteins, since the vast majority of fungal and bacterial strains appear to have the same intermotif length range, even if there are population-level differences.

We thank the reviewers for this comment. NRPS from bacteria and fungi share the same evolutionary ancestry but diverge in several characteristics (a nice review refers to PMID: 25156669). For example, in the pathway architecture, fungi NRPS ends usually with CT domain while bacteria NRPS usually ends with TE domain (PMID: 33941694). In the A domain pocket, fungi and bacteria also exhibit differences (PMID: 19923209). Fungal NRPS (most are siderophore-encoding) does not follow the typical NRPS co-linearity rule as that in bacteria, but instead iteratively uses A domains (PMID: 28534477, PMID: 34094397). We believe quantifying their differences may add to the mechanistic understanding of how NRPS functions.

However, it’s still unknown which parts of the NRPS sequence play key roles in such differences. In our motif-and-intermotif architecture of NRPS, we tried to answer this question from a statistical perspective. Following the reviewer’s comment, we validated our result on a larger dataset (dataset described in details in the answer to the last question of the reviewer). In 83,489 bacterial and 34,269 fungal C domains, we counted the C domain subtype distribution and found bacteria and fungus have significantly different preferences in C domain subtype, which makes it possible to determine whether the producer of NRPS in the metagenome fragment is bacteria or fungi. In the landscape of intermotif length distribution, our results locate potential differences between bacterial and fungal NRPS. We found the significant difference occurred in the length of interdomain (intermotif A10-Talpha1 and T1-C1). Although the distinctions between two kingdoms are still to be explored in detail, we provide some initial insights for further research.

Thank review’s comment for article structure. We will reorganize the article structure, remove the original last section, and add a new Section 1.3 for comparing the similarity and difference between bacterial and fungal NRPS.

Regarding how loop lengths in the A domain may assist substrate prediction, we noticed that the length of the A3-A4 and A5-G motif in the five loops influences the mapping from S-code to substrate specificity, in a similar way in both bacteria and fungi (Figure 6A-6B). Future substrate prediction algorithms that weight the loop length differences between query and reference A domains may improve in accuracy. However, length distributions of the other three loops differ significantly between bacteria and fungi (Figure 6A-6B). It is consistent with the A domain pocket of fungi differing from bacteria (PMID: 19923209). And in our larger datasets, most of the loop groups in bacteria are in groups 1/2/3 (37.8%, 28.4%, and 25.1%), while the dominant group in fungi is group 3 (85.9%). Taken together, intermotif length may influence substrate specificity by influencing 5 loops length group.

Finally, current algorithms for substrate prediction are highly dependent on prior knowledge. In these supervised learning-based algorithms, substrate prediction is akin to looking up a table of sequence signatures versus substrate. Our results in the manuscript show that substrate specificity and phylogenetic history are intertwined, meaning that this lookup should take into account Stachelhaus codes, phylum, and loop group versus substrate. However, for a specific phylum, there may not be enough data for accurate substrate prediction. Experienced research groups often have a wealth of unique data for specific NRPS classes, allowing for more accurate substrate predictions based on the insights in our manuscript. We believe that providing a substrate prediction method, rather than a prediction result, is more beneficial. If needed, Table S9 in the revision provides all the information about the A domains we collected, including known substrate specificity. We may consider adding a substrate prediction function to our online platform if there is sufficient demand from users

5. Two final broad points: first, this work might be stronger if results were validated against larger datasets. MiBIG consists of well-studied biosynthetic pathways, implicitly providing a potential source of bias: understudied and unculturable strains and difficult-to-characterize compounds and the biosynthetic diversity they represent are not present. This may bias analyses. Is similar motif conservation observed across bacterial CAT modules from, say, IMG? Across all bacterial C and A domains in IMG? Whether the answer is yes or no, it will help other researchers understand how widely applicable the conclusions in this manuscript are.

We thank the reviewer for these comments and questions. We chose the MiBiG database at the start of this project because we thought these were well-studied and alive biosynthetic pathways with known substrates. In the IMG database, most NRPS BGCs are annotated by antiSMASH with predicted substrates, without experimental validation. Therefore, we can’t calculate mutual information (MI) between residues in the C domain and its chirality subtypes, as well as MI between residues and A domain substrate specificity (Figure 1A-1B, fourth panel). In addition, in our prior study of various microbial NPRS systems, we observed that dead pathways, which are not expressed in the cell and frequently have disrupted domain structures in their sequences, are commonly present (cite the cEK science paper). We suspected that a larger database lacking experimentally validated live pathways might be interfered by these dead and disrupted gene clusters.

Nevertheless, as the reviewer suggested, after acquiring sequence signatures from MiBiG, it’s necessary to validate these motifs in a larger dataset. IMG-ABC (The Integrated Microbial Genomes - Atlas of Biosynthetic gene Clusters) database is the most comprehensive publicly available repository of predicted and experimentally verified BGCs. In this database, there are 70,277 BGCs predicted by antiSMASH v5 (up to 2022/11/19). However, the latest antiSMASH has been updated to version 6 and version 7 in beta, and it’s difficult to download and process large data from IMG-ABC in its own format. Therefore, we decided to mine BGC in known genomes from scratch using antiSMASH v6. We downloaded all complete genomes of bacteria (30,984), all assemble level genomes of fungi (3,672), and all assemble level genomes of archaea (6,093) from NCBI. After removing genomes with 99.6% similarity, there are 16,820 bacterial, 2,505 fungal, and 5,658 archaeal genomes (up to 2022/10/23. This dataset will be described in detail in another manuscript that will be online soon). Then BGCs in these genomes were annotated by antiSMASH v6. In this dataset, we found 99,575 BGCs (19,460 NRPS and 6,484 NRPS-like) in 15,296 bacteria genomes for 7,974 species and 57,531 BGCs (13,938 NRPS and 13,063 NRPS-like) in 2,331 fungi genomes for 1,799 species (Table S10 in the revision, "Overview" Sheet). In these larger datasets, we checked the conservation of 26 known sequence motifs (10 for A domain, 7 for C domain, 7 for E domain, 1 for T domain and 1 for TE domain), as well as the 3 novel motifs discovered in this manuscript. The results are summarized in Figure S7-S12 and Figure S15-S16 in the revision.

To avoid using dead NRPS BGCs, we filtered possible inactive domains by motif length because most active domains share a similar motif length. Motifs from bacteria and fungi NRPS are largely similar, with one exception in the E6 motif: it is RX(V/I/L)PXXGXG(Y/F)G in bacteria and RX(V/I/L)PXXGXXYF in fungi (Additional Figure 6, Figure S8 and Figure S10 in the revision). According to previous research, the consensus sequence of E6 was PxxGxGYG (PMID: 10021423), only consistent with the E6 sequence logo we observed in bacteria. It’s reasonable because the E domain sequences were mostly from bacteria at the time when the sequence motif was suggested. For the first time, NRPS motif consensus sequences from bacteria and fungi were identified in our updated dataset. These differences may also be applied to NRPS fragments from metagenomes, helping researchers to discriminate short contigs from bacteria or fungi.

To summarize, our results have been validated using larger datasets and are found to have wide applicability. The added insights from these larger datasets have been fully described in sections 1.3-1.5 of the revised manuscript.

**Additional Figure 6. E6 motif sequence logo in bacteria and fungi.**

**A.** E6 motif from 14,502 bacteria E domains

**B.** E6 motif from 3,982 fungi E domains

Second, the authors should consider how researchers working with NRPS pathways might be able to apply this data. What would actually be necessary for someone to read this paper and apply the insights to their own NRPS system? The authors don't actually discuss their NRPS Motif Finder tool in the main text or in the SI. Can users search for new sequences or only search for or browse previously evaluated sequences? Which of the analyses discussed will be performed, if it accepts new sequences? Are there expected limitations (will it not handle sequences with alternate domain architectures or additional domains)? A reader for this paper would not know, as it is currently written, and the data in the paper alone don't quite get the reader to the point of being able to apply the results to their system.

We thank the reviewer for these suggestions and questions.

The standardization of NRPS sequences has multiple applications for NRPS researchers, such as predicting A domain substrate, guiding re-engineering, and distinguishing bacterial and fungal NRPS. We added corresponding descriptions of the online platform and discussions on the application scenarios to Section 5 and the Discussion.

Regarding the data and code availability, the NRPS Motif Finder online version is based on Python, and the result could be downloaded in the format of Excel for further analysis. It takes any query amino acid sequences in FASTA format and can be interacted with by clicking on the website result page for more information. For researchers with basic programming skills, we recommend the NRPS Motif Finder Matlab version.

Regarding the uses of NRPS Motif Finder platform, it can be used to locate conserved motifs from any query sequence (and return “no domain detected” when no domain or motif can be detected). We currently use this tool for data standardization and sequence-to-function mapping in our and collaborators' projects, such as predicting substrate by A3-A6 in fungal peptaibol (<https://www.biorxiv.org/content/10.1101/2022.06.05.494846v1.full>) collaborated with Professor Wen-Bing Yin from the Institute of Microbiology in Chinese Academy of Sciences, defining the functional units in cEK NRPS collaborated with Professor Mohamed S. Donia at Princeton University (manuscript in submission), as well as predicting substrates and analyzing recombination events in *Pseudomonas* pyoverdines (NRPS siderophore) in our lab (manuscript in preparation).

In addition, precisely locating conserved motifs can help with re-engineering. NRPS Motif Finder could provide the position of the G-motif for re-engineering A domain substrate specificity by A subdomain substitution. NRPS Motif Finder now can also predict C domain subtype. It now can distinguish 18 subtypes of C domain, providing by far the most comprehensive C domain subtype classification tool. As a comparison, antiSMASH v6 can distinguish LCL, DCL, Cglyc, Dual, Starter, Cyc, and X subtypes, and NaPDoS v2 can distinguish LCL, DCL, Dual, Starter, Cyc, modAA, and Hybrid subtypes. These are subtypes dominant in bacteria but not in fungi. To our best knowledge, NRPS Motif Finder is the first online tool capable of predicting fungus C domain subtypes. And due to different C domain subtype preferences between bacteria and fungi, it further could be applied in distinguishing NPRS of bacterial or fungal origin from highly fragmented environmental sequences data and potential horizontal gene transfer (HGT) Other potential functions include providing the 5 loop lengths and loop group in A domains. These functions have been updated in the Matlab version of the NRPS Motif Finder.

The main limitation of NRPS Motif Finder is that it only supports main domains in NRPS: C/A/T/E/TE domain. Other additional tailoring domains such as methyltransferase or hydroxylase domain will be recognized as interdomain between main domains. It may be improved in future update.

We had put these descriptions of NRPS Motif Finder in the Section 5 and discussion in line 1015-1021 in the revision.

Minor issues:

1. Terminology - "Stachelhaus (specificity) code" is the standard terminology. I'm not sure that introducing a new term not really used elsewhere in the vast NRPS literature ("10-S") increases clarity.

We apologize for any confusion caused by the terminology used in our original manuscript. We found "Stachelhaus code" and "the specificity-conferring code" both are common terminology for “10-S codes”. In revision, we replaced “10-S codes” by "the specificity-conferring code" for clarity in the text.

2. Details are occasionally lacking in the methods: when sequences with high identity were removed (line 733), how were sets of high identity sequences clustered, and what cutoffs were used to choose a representative one? Where were the 2636 A domain sequences collected from (line 737), and how (they appear to be a mixture of UniProt and MiBIG IDs - are the UniProt sequences via SeMPI?) How and why were the different sector identification parameters defined for CAT, CATE, and CATC NRPS modules (line 813-820)? Similar issues are present throughout this section. In general, there should be enough detail to reproduce methods, and that detail is sometimes absent.

Due to data quantity constraints, we only removed sequences with high identities and analyzed the remaining sequences.

2636 A domain sequences were collected from SeMPI 2.0 (<https://doi.org/10.3390/metabo11010013> ). There are 2868 A domains in the supplemental material of this paper. And these sequences were originally a mix of UniProt and MiBiG. We removed sequences which had deleted in MiBiG database or UniProt. Finally, there are 2636 A domain sequences used in our study.

For sector identification in SCA, as mentioned in the main text (line 705-707), it is the convention to remove the dominant first mode, because it shows phylogenetic association. Two sectors of the second mode with opposite eigenvalues are important in analysis, as the major coevolution features of sequences. Identification of the other sectors is largely empirical (if needed): a sector needs to be discussed if it exhibits different sequence features from the previous two sectors, and has a relatively high eigenvalue.

3. I'd consider using letter or Roman numeral labels rather than color names for the sectors in Fig. 4A and 4C (and in associated discussion, in lines 441-447, and 469). Red/magenta are not very visually distinct, and red and green as adjacent colors will be a problem for people with colorblindness.

Thanks reviewer for comments. We used Roman numeral label in revision.

4. The colors of sectors 1-6 in Fig. 5A match the phylum colors, but my interpretation of section 4.1 and the caption was not that the sectors directly reflect phylum. I'd strongly suggest an alternate color scheme (or an uncolored but labeled diagram). Additionally, color keys needed to understand Fig. 5B are on the opposite end of the figure, and they look like they are associated with Figs. 5A/C. I'd try to redesign this figure for clarity.

We apologize for this confusion. We redesigned this figure for clarity.

5. "Light green" or "yellow green" would likely be a more widely recognized color term than "limon" (line 480, SI)

We thank the reviewer for the comment. We replaced “limon” by “yellow green”

6. Finally, there are numerous small errors (aspartic acid referred to as positively charged (Figure 2), lysine and arginine described as negatively charged (Figure 2, Table 1), typos like UniPort (line 737). An additional readthrough to catch and correct this sort of error would be advisable.

Thanks. We revised these problems.

## Reviewer #3:

The authors here present work focusing on NPRSs, a well-studied family of enzymes useful in particular for protein engineering and reengineering largely due to its modular structure. With the rapid expansion of sequencing techniques and subsequent sequencing data, the amount of available information on NPRSs has increased dramatically; however, with the increase in sequencing data has come an increase in annotation standards and variety making meta-level analysis leveraging widely-used techniques such as MSA difficult to implement within this enzymatic family. The authors provide their take on a standard architecture in which conserved core motifs within various domains are used as lynchpins for constructing a “coordinate system” for NRPSs. The authors then utilize this novel system for statistical and functional analyses including the identification of specific, conserved interdomain positions, interesting analysis motif and intermotif MI with respect to subdomain type, SCA and correlation analysis for residues within and between domains and motifs and the potential ability to distinguish between bacterial and fungal NPRSs (and more). Overall, the authors provide compelling evidence for the usage of this newly defined NRPSs categorization and this work should provide a great fit for PLOS Computational Biology after addressing the concerns discussed below.

(1) One major concern is the missing link or the consensus among different analysis: It would be great if authors try to find general link between different analysis that provides solid design.

We thank the reviewers for pointing out the need for improvement in the manuscript organization. In this revision, we removed the original last section, and added a new section 1.3-1.5 for comparing the similarity and difference between bacteria and fungi NRPS in a larger dataset.

The new article outline is

1.1-1.2. The standardized motif-and-intermotif architecture proposed.

Then, we introduced the applications of the architecture in sequence statistics, including:

1.3-1.4. C domain subtype classification,

1.5. A comparison of NRPS domains between bacteria and fungi in a larger dataset, and

2. The discovery of new motifs.

Then we moved to analysis of sequence couplings, including:

3. The use of SCA for NRPS reengineering boundary identification, and

4. Decoding the entanglement between substrate specificity and phylogenetic history in the A domain.

We hope this revised logic provides a clearer and more comprehensive overview of the research.

(2) The figure 1B (MI analysis) very interesting, with high degree of MI for A domain subtypes in 10-S A4-A5 region as expected. As a suggestion, I would appreciate any additional commentary on the C5-C6 inter motif region where the MI for C domain subtypes is nearly as high on average as C6 and higher than C7. Interestingly, residues of this region again appear in Figure 4 as containing some green and red (C-motif and A-motif spanning sectors).

We thank the reviewer for this comment. In brief, the C4, C6, and C7 motifs are not highly conserved. They diverge according to the subtypes of C domains (Figure S1 in the revision). Particularly, the C5-C6 intermotif is involved in the T_n_:C_n+1_ interaction that is related to substrate chirality.

In detail, careful inspection of related regions in a multi-module NRPS structure is required to answer the reviewer’s question. We checked the C5-C6 intermotif in the structure of LgrA, the only structure that contains multi-module NRPS in multiple conformations so far characterized by Reimer et al. (F+A+T+C+A+T, F is the formylation domain, PMID: 31699907, Figure 3B-3D in the revision). We noticed that the C5-C6 intermotif mainly consisted of an α helix (α9), and it’s located in the interface between the donor T domain and the recipient C domain of the next module (T_n_:C_n+1_). In the work of Reimer et al., the authors indicated that the phosphopantetheine (ppant) arm of the T_n_ domain needs to pass the channel formed by α4 and α9 (that is, the C-terminal of the C5-C6 motif) of C_n+1_ domain to perform its function. They also noticed some coevolution positions between the T_n_ and C_n+1_ domains by direct coupling analysis (DCA, another coevolution analysis method focusing on site-to-site covariation), and constructed 4 mutation experiments accordingly (A1008, R1051, P1086 and L1088). They found that three out of the four mutations significantly impaired the function of the C domain and decreased tripeptide production. Two mutations (P1086 and L1088) locate in the C5-C6 motif (α9, C5-C6 motif ranges from 1065 to 1089), and one locates in the C5 motif (R1051, C5 ranges from 1035 to 1064). Therefore, T_n_ and C_n+1_ are functionally linked by the C5-C6 motif in the T_n_:C_n+1_ interface.

In summary, the T_n_ and C_n+1_ domains are both involved in substrate chirality, and the C_n+1_ domain has been suggested to interact with the T_n_ domain by the C5-C6 intermotif. This involvement of chirality also explains the multiple peaks of mutual information (MI) in the C5-C6 intermotif.

Also, in terms of coevolving sectors, it’s reasonable that some positions (798, 809) in the C-terminal of the C5-C6 intermotif are in the green sector, which predominantly locates in the C domain. Meanwhile, the N-terminal of the C5-C6 intermotif is adjacent to the G-A6 intermotif in the crystal structure, which is part of the A domain substrate binding pocket (A3-A6, Figure S29 in the revision). Therefore, possible coevolution between the C5-C6 intermotif and the A domain pocket could occur (mostly covered by the red sector), which may explain why there is a position (788) in the C5-C6 region falling in the red sector (the A domain pocket-related sector).

Additional discussions on this region has been added to line 263-273 and 728-733.

(3) In section 2.2 the authors identify a conserved motif between the C and A domains and falls near the low MI region of the far-right orange in Figure 1A bottom panel. This motif then has low MI with C domain subtypes, but also forms bonds with A4. Were any of these positions found to be within the magenta or red sectors from SCA? If not, can the authors provide a comment why one might find conserved positions consistently forming H-bonds with A motif absent within the A-spanning or A-heavy sectors?

We thank the reviewer for this comment. In the multiple sequence alignment shown in Figure 3A in the revision and Additional Figure 7, we checked the positions of the Aα1 motif locating between the C and A domains, and found the position 1180 in the Aα1 motif was indeed in one A-heavy sector (the top 5-th sector (>.05), λ (eigenvalue)=22) and two A-spanning sectors (the top 4-th sector (<-.05), λ=26, and the top 7-th sector (<-.05), λ=17). In addition, position 1248 in the A1 motif interacts with the Aα1 motif, and it is also in this top 5-th A-heaving sector. Four other positions (1697, 1698, 1700 and 1702) after the A4 motif interact with the Aα1 motif, and they are also in these two A-spanning sectors (the top 4-th(<-.05), λ=26 and the top 7-th(<-.05), λ=17).

**Additional Figure 7. Top2-8 sectors in C+A+T module.**

However, none of the positions in Aα1 motif are involved in the magenta nor the red sectors in Figure 5A in the revision. Eigenvalue λ of a SCA mode reflects its significance. (Figure S28 in the revision). In this study, the maximum is 166, representing phylogenetic association instead of functional coupling. Next are the green (the top 2nd(>.05), λ=63) and magenta (the top 2nd(<-.05), λ=63) sectors. And the red sector is the top mode covering A domain pocket (the top 6-th (<-.05), λ=17). More details refer to Section 3 and method at line 1143-1156.

In summary, the Aα1 motif is not involved in the green, red, or magenta sectors, which is consistent with the fact that it has little information about chirality, and is not involved in substrate specify. It does locate in an A-heavy sector and an A-spanning sector, but these two sectors have relatively low eigen values. It suggests that Aα1 motif related modes are not among the major modes of the NRPS coevolution. However, it doesn’t mean it’s unimportant. Highly conserved sites are generally not involved in coevolution, as they seldom show variability. Also, it has been reported that Aα1 is a natural recombination site in Myxobacteria (PMID: 28128551) and applied in synthetic biology for diverse NRPS products (PMID: 30319751).

We added short descriptions about the modes of Aα1 motif in line 674-676.

(4) Figure 4C; I would be interested in additional discussion with this. Particularly, it seems that there are several residues in the green sector that correlate strongly with the red sector – by inspection, it appears that some of these “span the gap” to correlate with the red sector positions for which magenta correlations are noticeably absent.

As mentioned in the previous question, the green and magenta sectors are the top2 co-evolution mode of the C+A+T module, formed by opposite directions of the same eigenvector (>.05 for the green sector and -.05 for the magenta sector), so positions in the two sectors do not overlap. Meanwhile, the red sector is the 6-th mode, making it possible to intersect with green or magenta sectors. In detail, there are 5 positions (residue 85, 559, 718, 941, 942 in the MSA of Figure 5 in the revision) shared between green and red sectors, and 6 positions (1164, 1351, 1358, 1605, 1631, 1635, 1965) shared between magenta and red sectors. And in the green sector, most positions are highly correlated. Therefore, overlapped positions with strong correlation were observed between the green and red sectors, which contributes to the “several residues in the green sector that correlate strongly with the red sector” phenomenon observed by the reviewer. The correlation between positions in the magenta sector is relatively weaker. Therefore, no strong correlation between magenta and red sectors can be observed, even between the six positions. Also, the strict “no overlapping residues between green and magenta” contributes to the “span the gap” phenomenon, as whenever there is a red-green correlation, the red-magenta correlation needs to be absent.

It’s noted that the 5 positions shared by green and red sectors are all in the C domain, and the 6 positions shared between magenta and red sectors are all in the A domain. And on average, the red sector is more correlated with the magenta sector. It makes sense because the magenta sector spans the entire C+A+T module and the red sector is mostly found in the A domain. However, it’s noteworthy why the green sector (C-heavy sector) and the red sector (A domain pocket related sector) shared 5 positions (85, 559, 718, 941, 942). It may suggest a connection between C domain and A domain substrate selectivity, as discussed in previous research (PMID: 27105282 and 32055321).

Additional discussions were added to line 717-728.

(5) In the second paragraph of section 4.2 the authors state they compare loop amino acid (AA) length with Euclidean distance between A domains and then state that loop-based distance is connected with similarity of sequence alignments. The only way this statement makes sense is if the authors are suggesting AA length is directly correlated with sequence alignment; if this is the case, the authors should state this. Also, the authors state this loop-based distance is connected with the domain A phylogeny (Figure 5B); this does not appear to be the case at all based upon what can be discerned from Figure 5B outside of the Firmicutes-Loop group 1 relationship.

We thank the reviewer for this comment. In our analysis, loop lengths are relatively independent with the multiple sequence alignment (MSA), as the starts and ends of loops are highly conserved motifs and Stachelhaus codes. These highly conserved sequence pieces help to anchor the alignment, so the length of the variable regions between motifs (i.e., the loop length) does not influence the alignment much (Figure S43 in the revision). However, in variable regions, discrepancies in AA length do indeed correlate with larger sequence distances. We’ve added more clarification in line 842-847.

In addition, we added a causal diagram to elaborate factors influencing the sequences of A domains (Figure S43A in the revision, more about causal diagram for causal inference can be seen in PMID: 9888278): A domain sequences are shaped by phylogenetic history (what species the A domain comes from), evolutionary events of genes (e.g. horizontal gene transfer, gene duplication), and selection pressure for substrates (such as chemical requirement for biological activity). A domain sequence determines Stachelhaus codes and pocket scaffolds (5 loops, Figure S43B-S43C in the revision). Finally, A domain substrate binding domain, characterized by Stachelhaus codes and pocket scaffold, determines the substrate. Therefore, loops are not directly coupled with Stachelhaus codes (Figure S43B in the revision). Instead, they are connected by substrates (pocket, Figure S43D in the revision). In our manuscript, we used the loop length group to represent the scaffold information, which is a reasonable simplification. And we are trying to use sequence information about loops to improve A domain substrate prediction, as loop groups could offer insights in reducing the variability of the Stachelhaus codes (Figure S34-S41 in the revision).

As shown in causal diagram, loop distance is connected with A domain phylogeny, but the connection is weak due to other influencing factors (Figure S43C in the revision). There is degeneracy in observations (Figure S34-S41 in the revision) due to redundancy between information provided by Stachelhaus codes and loops, similar to synonymous codons in genetic codes. It’s why some Stachelhaus codes can hardly be used across phyla (Figure 6C in the revision).

In conclusion, our result emphasized the significance of loop length for A domain substrate prediction, which should be taken into account in future A domain substrate predictions.

(6) The statement at the end of section 5 (line 590) “In summary, bacterial and fungal NRPS show significant difference in the length of intermotif. It makes it possible to distinguish between bacterial and fungal NRPS in our standardized architecture.” seems too strong. While the authors provide evidence that there are some differences in intermotif length between bacteria and fungi, it does not appear that this will be sufficient to distinguish a given NPRS as belonging to either bacteria or fungi.

We thank the reviewer for this comment. We validate our result on a larger dataset in Section 1.3. In these 16,820 bacterial and 2,505 fungal non-redundancy genomes, we checked whether a significant difference occurred in the length of some interdomain regions (intermotifs A10-Talpha1 and T1-C1, Figure S13 and S18 in the revision). It could be applied to distinguish between bacteria and fungi. Moreover, we found another intuitive approach. We found bacteria and fungus NRPS have significant different preferences in C domain subtype: bacterial C domains mainly consist of SgcC5 (28.97%), LCL (19.42%), DCL (12.32%), Starter (11.45%), Cyc (6.73%), Dual (5.85%) and Cglyc (5.75%), while most fungal C domains are FUM14 (30.12%), CT-A (13.89%), CT-DCL (13.77%), CT (10.98%) and Hybrid (6.90%). So we could also infer, just by its C domain subtype, whether the producer of a NRPS fragment in the metagenome sequencing is bacteria or fungi.

As also other minor points

(7) Figure 5 – I found the color-coded keys for this figure difficult to interpret as keys for substrate type are used in B and C are placed adjacent to A, the loop-group key is only used in B and is placed next to A and the phylum group, used in all A B and C is placed next to C but is necessary to correctly read A.

We apologize for this confusion. In the prior version, the color codes of sectors 1-6 in Figure 5A (Figure 6A in the revision) overlapped with the phylum colors, but actually didn’t reflect phylum. We redesigned this figure for clarity.

(8) The sentence “The sector containing positions densely covering the A domain pocket region appears till the sixth biggest mode (Figure 4A, red dots)” lines 441-443 is unclear – perhaps a typo in the usage of the word “till”?

Thanks. We revised this sentence into “The sector densely covering the A domain pocket region appears in the sixth biggest mode (Figure 5A, labeled as IV(-) sector with red dots)” at line 718-720.

(9) Line 333 sentence “conserved suggests causes for the conservation is beyond forming hydrogen.” should end in “bonds.”

Thanks. This sentence was revised.

(10) Violin plots in the second panel in Figure 1 is very small, hard to understand needs revision.

Thanks. We revised this figure with a bigger violin plot.

(11) Figure 3 B it is stated in caption “The mutated tryptophan380 was marked in purple” . It should be not purple but magenta

Thanks. We revised this error.
