# Supplementary material for: Knowledge-guided data mining on the standardized architecture of NRPS: Subtypes, novel motifs, and sequence entanglements
Source: PLoS Comput Biol. 2023 May 15;19(5):e1011100. doi: 10.1371/journal.pcbi.1011100 (PMC10212144; doi:10.1371/journal.pcbi.1011100)
Supplement: Attachment — Submitted filename: revision3-lzy.docx [file pcbi.1011100.s064.docx]

Dear Editors and Reviewers:

We are pleased to submit our revised manuscript entitled "Knowledge-guided data mining on the standardized architecture of NRPS: subtypes, novel motifs, and sequence entanglements." The revision includes two Word files for the clean and uncleaned main text revisions with the prefix "main_draft_standardNRPS_HRL_2023_04_02", a Word file for SI information "SI_draft_standardNRPS_HRL_2023_0322" containing Figure S1-S45, Table S8 and Table S12-S13, ten Excel files including Table S1-Table S7 and Table S9-S11, five compressed files including codes, sequences, phylogenetic tree, and HMM files. The content of five compressed files is described in the SI Word file. We would like to thank the editors and reviewers for their insightful comments and recommendations, which have helped us immensely in improving the work.

The detailed responses to each reviewer are listed here. Our responses are in blue text:

***********************

Reviewer's Responses to Questions

**Comments to the Authors:
Please note here if the review is uploaded as an attachment.**

Reviewer #1: Zhang et al. provide a very comprehensive overview of conserved NRPS domain motifs and inter motif sequences. However, compared to the initial manuscript the revised version did not substantially improve. Nevertheless, I value the authors work, because I am still convinced that it provides a well overdue update on NRPSs.

We are appreciative of the insightful insights provided by the reviewer. We have thoroughly edited the manuscript to correct any grammatical and language errors.

Prior to publication the following points must be solved:

Major comments:
- Fig. 3 & 4 are the same.
- Caption of Fig. 3 doesn't match or vice versa

We apologize for this mistake, and updated the correct Figure 3 in the revision.

Minor comments:
- The manuscript would still benefit from correction by a native speaker - there are still too many mistakes. Some parts obviously improved but others did not.

We thank the reviewer for the suggestion. We carefully inspected the complete text's grammar and language with the help of the Grammarly premium version, and corrected any errors we found.

Reviewer #2: The restructuring and addition of additional background have substantially improved the clarity and accessibility of the paper. It's also very reassuring to see that some of the extended analyses on larger datasets (or analyses using alternate methods, e.g. Phyre2 vs. SWISS-MODEL vs. AlphaFold2) have validated (and even strengthened) some of the authors' conclusions! In its revised form, I think the manuscript is significantly more compelling, and it will be much easier for other groups to apply its conclusions to their attempts to investigate and/or engineer NRPS systems. However, a few issues still remain in this draft.

We appreciate the reviewer's valuable comments. We are happy that this manuscript has been significantly improved from the reviewer's thoughtful suggestions last time. And remaining issues have been solved in this revision. Again, we greatly appreciate the detailed comments of the reviewers, which makes this manuscript more beneficial for the scientific community.

Major issues:
1. Regarding the G-motif: the preliminary data regarding the effect of module position in multimodule pathways are quite intriguing! However, if those data are not included in this manuscript, it unfortunately remains premature to describe the G-motif as "critical" or that the flexibility of glycine at that position is "essential" (line 853) - despite the high conservation of that residue, what's going on is clearly more complex than an absolute reliance on a glycine at that position for activity. Even the preliminary data in in Additional Figure 3 don't show that a glycine at any position is actually vital for enzyme activity (even in Module 1, other small amino acids are tolerated with a moderate hit to activity). Additionally, statements in the paper should describe the data presented in the paper, and in this context, given the data that are in the paper (specifically the G409A and G409R results in Fig. 4), I do not think it is appropriate to say "Our mutation experiment supported the critical role of the conserved glycine in the G-motif, and indicated that the flexibility of glycine is important in this position" (less flexible residues, including those with different chemical properties, retained high activity.) Similarly, it's premature to say that "Our results implied that the flexibility of glycine in the G-motif is essential for conformational change during A domain functions, therefore conformational restrictions in the G-motif impede A domain function." It's quite plausible that that is part of what's happening (and part of the reason for the different results in multimodule systems), but the data actually in this paper (diminished natural product production for a set of variants in a single module in a single NRPS system) do not provide direct evidence for the broader mechanism of G-motif function. More conservative and less definitive statements regarding the G-motif remain appropriate.

We thank the reviewer for these comments. We have changed the statement about the G-motif to a more conservative tone, like “potentially important roles” at line 648, and removed “indicated that the flexibility of glycine is important in this position” at line 648.

2. Some details remain missing in the methods section. E.g. a maximum likelihood tree for the condensation domain is shown in Fig. 2, but the alignment and parameters (and even the tool) used to generate it are not actually described. Structural modeling and alignment are mentioned in the text and in the SI but not in the methods (and it's unclear how the "simulated mutation" was performed, and what methods were used to explore tryptophan sidechain conformations in this simulation). It's fine to cite previous papers where appropriate, but all methods do need to be described in the methods section, either in experimental detail or as citations.

We thank the reviewer for these questions about the methods. We rewrote the part of methods to clarify the related process, from line 1145 to 1162.

3. Figure 3 appears to be the wrong figure entirely (it seems to be a duplicate of figure 4, and the captions don't match the content at all.)

We apologize for this mistake and updated the correct Figure 3 in the revision.

Minor issues:
1. Many paragraphs are quite long. Splitting them at natural points (e.g. in line 76, the paragraph switches from describing NRPS modules to discussing engineering efforts) would improve readability.

We thank the reviewer for the suggestion. We split multiple long paragraphs to improve readability.

2. The captions in Fig. 1 should clarify colors for all violin plots. It looks like C must be grey, A must be blue, T is yellow, maybe E is grey (hard to see at printed scale)?, TE is red? But this is not stated except for the first violin plot.

We thank the reviewer for these questions. We added the statement about violin plot colors in the captions of Figure 1.

3. In Fig. 2: I'd recommend using red and blue rather than red and green for bacteria vs. fungi for colorblind readers. The black tickmarks under each position in the logos overlap the letters in ways that might impede legibility. It's unclear what "Three NRPS organizations do not have known examples" means - is it that these NRPS layouts are observed in BGCs in genomes but none of the products that they produce have yet been structurally characterized? (In context, it's a little unclear, since it could also be interpreted as "these layouts could theoretically exist but have not been observed in genomes")

We thank the reviewer for these questions and suggestions. “Three NRPS organizations do not have known examples” means that the function of “KnownClusterBlast” in antiSMASH cannot locate an experimentally characterized BGC for these organizations. However, two days after we submitted the first revision of the manuscript, we found that a new function, “MIBiG cluster comparison” in antiSMASH v6, performed better at locating characterized BGC homologous. Therefore, we have fixed the problem of “NRPS organizations do not have known examples”.

4. In section 1.5, it might be worth noting what percentage of NRPS pathways actually contain only high-confidence C domains, i.e. how big was the dataset from which these were extracted and what percentage of the dataset is being analyzed in the end. (The conclusions risk being a little circular: the high-confidence NRPS clusters are dominated by well-studied types of siderophores because those systems are, well, well-studied.) It would help to clarify for the reader how representative these systems actually are so that they don't overapply conclusions to the larger NRPS dataset.

We thank the reviewer for the suggestion. We added the count of the whole dataset in the revision, at line 461-462.

5. Line 577-578: "We observed that two amino acids, N577 and F493 in LgrA, have a size near glycine in the G-motif (G505)." It's unclear what this is saying? Asn and Phe aren't similar in size to Gly and aren't in the G-motif specified in the prior page. Rephrase for clarity?

We thank the reviewer for these questions. It should be “location”, rather than “size.” We apologize for this mistake and have fixed it in this revision.

6. Line 688: It's confusing to find Glu grouped together with "other conserved aliphatic and aromatic amino acids" (it is neither) that form the hydrophobic core of the domain? I'd clarify the description or its potential interactions, since it's a key part of this motif.

We thank the reviewer for these questions. “other conserved aliphatic and aromatic amino acids” means the other conserved aliphatic and aromatic amino acids between E700 and L711, rather than E (Glu), is an aliphatic or aromatic amino acid. We apologize for this confusion and have clarified the description at line 709.

7. Line 1181 - more detail is appropriate here (e.g. what was the LC gradient? What was the mobile phase? Were data collected in positive or negative ion mode, and what were the other mass spec parameters?) Again, the methods do need to be available somewhere, as a citation or as part of the methods section. (If the LC method is the method described in lines 1207-1214, I'd put all that information in one place.)

We thank the reviewer for pointing it out. We merged two parts of the experimental method into one paragraph with adjustments in orders, at line 1218-1228, to introduce the definition of LC gradient and mobile phase first before using them.

8. References - format varies. (Sentence case vs. All Capitalized Words, sometimes species names and gene names are not italicized, journal names are sometimes abbreviated and sometimes not (and sometimes capitalized and sometimes not), etc.) These need to conform to the PLOS reference style for publication (see: https://journals.plos.org/ploscompbiol/s/submission-guidelines)

We thank the reviewer for pointing it out. We unified the format of references in this revision.

Typos:
There remain numerous small errors in grammar and spelling. The manuscript would benefit from a careful editing pass. I've listed some examples but this should not be assumed to be a complete list:
1. "boarders" rather than "borders" in Fig. 1

Thanks. These words have been revised in Figure 1.

2. Inconsistent description of sequence conservation - PXXGXXYF vs. PxxGxGYG (capital vs. lower case X)

Thanks. It has been fixed at line 328.

3. obtained from "literatures" rather than "the literature" in line 354.

Thanks. We have corrected it.

4. "ester- and amide- bond" rather than "ester and amide bonds" in line 363

Thanks. We have corrected it.

5. "assigns" not "assign" in line 390 - antiSMASH is probably best thought of as singular in this context.

Thanks. We have corrected it.

6. "lichenysins synthase" and "depsipeptides or coprogens synthetase" - in both cases the plurals should be on "synthetase"

Thanks. We have corrected them.

7. "top 29th in organizations" in line 472-3 - should probably be "29th most common organization"? ("top (number)" is usually used for a range of numbers, not a single one)

Thanks. We have corrected it.

8. ε, not e in "poly-e-Lys" in line 591

Thanks. We have corrected it.

9. "Both" is inappropriately capitalized in line 743 (and "work of XUC" and "work of XU" in that line and the next should be rephrased - it's contextually unclear what XUC and XU are, and I might try to more explicitly state what the product of the pathway was and the module layout, rather than just mentionining the sector affected by the cut site.)

Thanks. We have corrected it.

10. "2+" should be a superscript in line 777

Thanks. We have corrected it.

11. "dhb" in Fig. 6 vs. "Dhb" in line 853 vs. lowercase again in Fig. S33

Thanks. We have corrected all of them into dhb.

12. "NRPS undergoes" - rephrase, probably "NRPS complexes undergo substantial..."

Thanks. We have corrected it.

13. line 1062 - should be "from" not "form"

Thanks. We have corrected it.

14. line 1072 - should be "Clustal" not "Cluster"

Thanks. We have corrected it.

15. line 1173 - "Escherichia coli" should be italicized.

Thanks. We have corrected it.

16. line 1180 - is "3 mm" supposed to be a micron? (That is, is it referring to 3µm particle size?)

Yes, we have corrected it.

Note that these issues extend to SI material, e.g. "Hybird PKS-NRPS" (not "hybrid") in Table S1 or "they in fungi" in line 215 & 216 of the SI text (I think these lines are saying that the numbers of bacterial and fungal sequences differ for the A1-Tα1 and Tα1-T1 analyses for various reasons? Note that this should also be fixed in the Fig. S14 caption). Similarly in line 243, it should probably "the bL subtype" not "bL the subtype." And in Fig. S42, the plot titles should contain the word "between" not "betweem", Fig. S45 should refer to "the literature" or "publications" or "published data" or something along those lines, not "literatures", and the SI references should be correctly formatted as well.

We thank the reviewer for the detailed suggestions for typos. We have fixed all mistakes.

Reviewer #3: The authors addressed all the concerns in this revised version.
